# Valine-glutamine (VQ) motif coding genes are ancient and non-plant-specific with comprehensive expression regulation by various biotic and abiotic stresses

**DOI:** 10.1186/s12864-018-4733-7

**Published:** 2018-05-09

**Authors:** Shu-Ye Jiang, Mayalagu Sevugan, Srinivasan Ramachandran

**Affiliations:** Genome Structural Biology Group, Temasek Life Sciences Laboratory, Research Link, National University of Singapore, Singapore, 117604 Singapore

**Keywords:** *Arabidopsis thaliana*, Bacteria, Co-expression, Evolution, Fungi, Nematode, *Oryza sativa*, VQ motif

## Abstract

**Background:**

Valine-glutamine (VQ) motif containing proteins play important roles in abiotic and biotic stress responses in plants. However, little is known about the origin and evolution as well as comprehensive expression regulation of the *VQ* gene family.

**Results:**

In this study, we systematically surveyed this gene family in 50 plant genomes from algae, moss, gymnosperm and angiosperm and explored their presence in other species from animals, bacteria, fungi and viruses. No *VQs* were detected in all tested algae genomes and all genomes from moss, gymnosperm and angiosperm encode varying numbers of *VQs*. Interestingly, some of fungi, lower animals and bacteria also encode single to a few *VQs*. Thus, they are not plant-specific and should be regarded as an ancient family. Their family expansion was mainly due to segmental duplication followed by tandem duplication and mobile elements. Limited contribution of gene conversion was detected to the family evolution. Generally, *VQs* were very much conserved in their motif coding region and were under purifying selection. However, positive selection was also observed during species divergence. Many *VQs* were up- or down-regulated by various abiotic / biotic stresses and phytohormones in rice and *Arabidopsis*. They were also co-expressed with some of other stress-related genes. All of the expression data suggest a comprehensive expression regulation of the *VQ* gene family.

**Conclusions:**

We provide new insights into gene expansion, divergence, evolution and their expression regulation of this *VQ* family. *VQs* were detectable not only in plants but also in some of fungi, lower animals and bacteria, suggesting the evolutionary conservation and the ancient origin. Overall, *VQs* are non-plant-specific and play roles in abiotic / biotic responses or other biological processes through comprehensive expression regulation.

**Electronic supplementary material:**

The online version of this article (10.1186/s12864-018-4733-7) contains supplementary material, which is available to authorized users.

## Background

*Valine-glutamine (VQ)* genes (*VQs*) encode VQ motif-containing proteins (VQs), which were generally regarded as a group of plant-specific proteins with the conserved VQ motif structure FxxhVQxhTG (F, Phenylalanine; x, any amino acid; h, hydrophobic residue; V, Valine; Q, Glutamine; T, Tryptophan, G, Glycine). VQs were phylogeneticly clustered into different groups, which were dependent on analyzed genomes [1–4]. However, no classification was reported, which is based on multiple species including non-seed plants.

More than half of identified *VQs* were up- or down-regulated by abiotic stresses or phytohormones in rice [5], maize [6], and Chinese cabbage [4]. In *Arabidopsis*, rice and other plants, *VQs* were differentially regulated by not only abiotic but also biotic stresses [2, 5, 7–9]. Many *VQs* have been functionally characterized and played roles in response to various pathogens including *VQ16*, *VQ21*, *VQ22* and *VQ23* [10–15]. VQs function as regulators by interacting with other proteins. Evidence showed that many WRKY transcription factors might interact with VQs [16]. These interactions might stimulate DNA-binding or transcription-activating activity [8, 12]. Sometimes, the interaction also repressed the DNA-binding activity of a WRKY [17]. The VQ-WRKY interactions also regulated expression level of downstream genes [18]. Their interactions are sophisticated, which were regulated by not only phytohormones, abiotic or biotic stresses [3], but also additional signalling components such as kinase [13]. Besides WRKYs, SIB1 and kinases, VQs also interacted with PIF (a basic helix-loop-helix (bHLH) transcription factor) [8]. Since the report of the first VQ protein [1], genome-wide identification and characterization was carried out in several genomes including *Arabidopsis* (34 *VQs* [3]), soybean (74 *VQs* [19]), maize (61 *VQs* [6]), Chinese cabbage (57 *VQs* [4]) and grapevine (18 *VQs* [20]). However, no enough data is available for the analysis of their evolutionary histories and expansion mechanisms in this family. In this study, we identified these gene family members from 50 plant genomes, 12 of which were selected for further investigation on their selection force, evolutionary history and expansion mechanisms. We then surveyed the presence of this gene family in other species from animals, bacteria, fungi and viruses. We have also investigated the expansion patterns among species from the *Oryza* genus. We then surveyed expression profiling of *VQs* in different tissues and under various abiotic and biotic stresses as well as phytohormone treatment. Our analysis provides some new insights into the origin, evolution and comprehensive expression regulation of this gene family.

## Results

### Plant genomes encode variable sizes of *VQs*

**Fig. 1:**
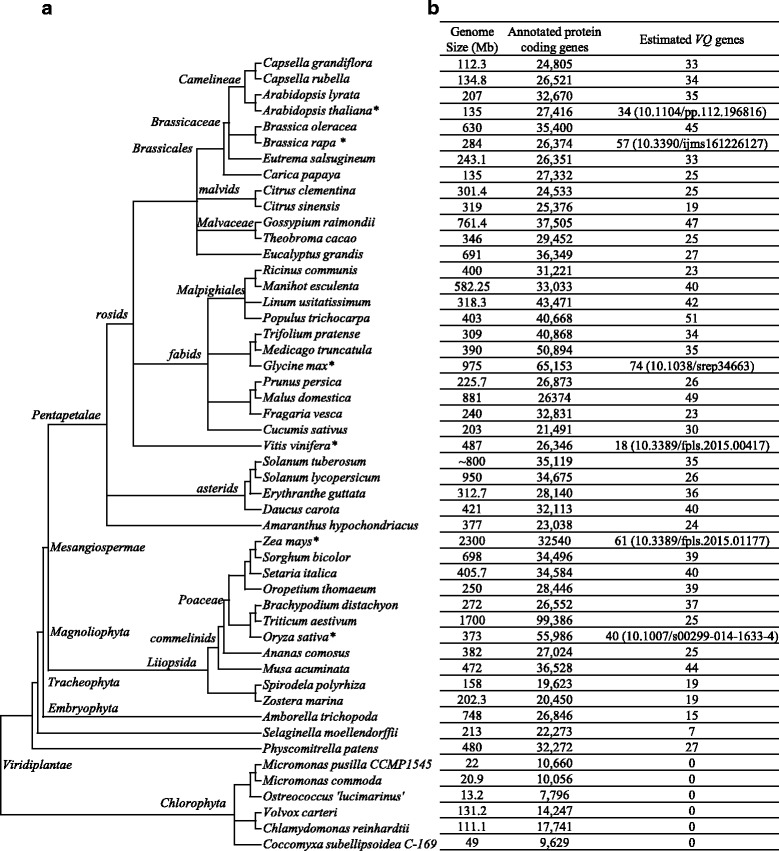
Genome-wide identification of *VQ*s in 50 species. **a** Phylogenetic tree of 50 species. The tree was constructed according to the data retrieved from the NCBI Taxonomy database (http://www.ncbi.nlm.nih.gov/taxonomy). **b** Genome size (Mb), numbers of annotated protein coding genes and numbers of identified *TPSs* in these 50 species. We identified *VQs* in each species using HMM profiling searches. We constructed the HMM profiling using the key motif sequences from the Pfam database (PF05678, https://pfam.xfam.org/). * *VQs* in *Arabidopsis thaliana*, *Brassica rapa*, *Glycine max*, *Vitis vinifera, Zea mays and Oryza sativa* were previously identified and their references were also listed

To better understand the general profile of *VQs* in the low and higher plant kingdom, we identified *VQs* in 50 completely sequenced genomes from algae, moss, gymnosperm and angiosperm by Hidden Markov Model (HMM) and BLASTP searches (Methods). All these genome sequencing data have been published and related information was listed in Additional file 1: Table S1. The searches showed that all 6 genomes from Chlorophyta encode no *VQs*, indicating the possible absence of this gene family in green algae. We then downloaded protein databases from 12 other algae genomes including *Porphyridium purpureum* (http://cyanophora.rutgers.edu/porphyridium/), *Cyanidioschyzon merolae* (http://merolae.biol.s.u-tokyo.ac.jp/), *Bigelowiella natans*, *Ectocarpus siliculosus*, *Galdieria sulphuraria*, *Gracilaria chilensis*, *Gracilariopsis lemaneiformis*, *Guillardia theta*, *Phaeodactylum tricornutum*, *Porphyra pulchra*, *Thalassiosira oceanica*, and *Thalassiosira pseudonana* from the NCBI database (http://www.ncbi.nlm.nih.gov/genome/). No VQs were detected by the HMM searches against these 12 genomes. Thus, the VQ family is not necessary for these Chlorophyta genomes. We then focused on the remaining 44 plant genomes. These genomes encode variable sizes of *VQs* ranging from 7 to 74 (*Glycine max*) (Fig. 1). The locus names, physical positions and protein sequences were listed in Additional file 2: Table S2. We also downloaded the *Marchantia polymorpha* protein sequences from the NCBI database (https://www.ncbi.nlm.nih.gov/genome/?term=Marchantia+polymorpha) and then carried out the HMM and BLASTP searches. We have detected 6 *VQs* in the liverwort genome. Thus, the *VQ* family is presented in liverwort, moss, gymnosperm and angiosperm but not in Chlorophyta.

### VQ motif coding genes were also detected in non-plant species

**Fig. 2:**
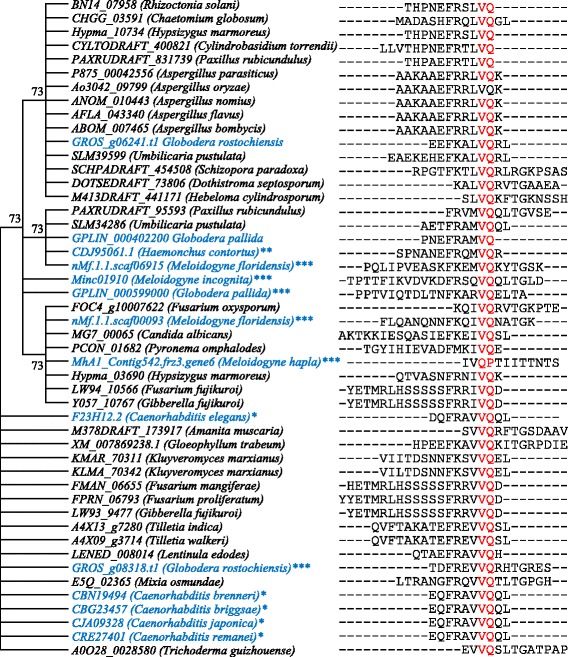
Amino acid sequence alignment and phylogenetic analysis of VQ motifs from nematode and fungus species. Motif sequences of 14 VQs from nematode (blue fonts in left column) and 34 VQs from fungi (black fonts in left column) were submitted to sequence alignment (right column) by Clustal X2 (see Methods). The aligned sequenced were used for phylogenetic tree construction by using the Maximum Likelihood method. Bootstrap values (> 50) were shown next to the branches. Conserved residues “VQ” in all species were highlighted by red fonts. The star “*”, free-living nematodes; “**”, Animal parasitic nematodes; “***”, Plant parasitic nematodes

Except for Chlorophyta, *VQs* could be detected in all tested plants. To our knowledge, no data were reported on the presence of *VQs* in non-plant genomes. We have carried out a genome-wide identification of *VQs* in 43 nematode genomes by the HMM searches. These genomes were from 43 species including 10 free-living, 10 human parasitic, 13 animal parasitic (2 for entomopathogenic) and 10 plant parasitic nematodes (Additional file 3: Table S3). No *VQs* were detected in 10 human parasitic nematodes. Totally, we have identified 14 *VQs* in 11 out of 42 genomes (Additional file 3: Table S3). Most of them contained only partial VQ motifs. These 11 genomes were from 5 free-living, 1 animal parasitic, and 5 plant parasitic species. All the 5 free-living and 1 animal parasitic nematodes encoded only one *VQ* in each species. For the plant parasitic nematodes, 2 of them encoded only one *VQ* and the remaining 3 species encoded two *VQs* in each genome. On the other hand, a total of 249 fungus species, whose genomes were completely sequences, were used for the genome-wide identification of the *VQ* family. Their annotated protein sequences were downloaded from the EnsembleGenomes database (http://ensemblgenomes.org/info/access/ftp) and were then submitted to the HMM searches. We have identified a total of 34 *VQs* from 29 fungus species and 5 of the species have 2 *VQs* in each genome. Similar to the nematode *VQs*, most of these *VQs* encoded only partial VQ motif sequences. VQ motif sequences from the nematodes and fungi were aligned by Clustal X2 (http://www.clustal.org/clustal2/) and we found that only two amino acid residues “VQ” were conserved among all VQ motifs (Fig. 2). The aligned sequences were then used for phylogenetic analysis and the constructed tree was shown in Fig. 2. In the VQ motif-based tree, not all VQs from the nematodes were clustered together and some of them were grouped with the VQs from fungi (Fig. 2). The results showed the inconsistence between the evolution of VQs and their species divergence. Besides nematodes and fungi, we have identified several putative VQs from some bacterial species but no VQs were identified from viruses. Protein database from a total of 50 bacterial genomes were submitted to the HMM searches. As a result, 8 VQs were identified from 8 bacterial species including *Aeromonas diversa*, *Enterobacter* sp. *BWH64*, *Lactobacillus camelliae*, *Lactobacillus manihotivorans*, *Lactobacillus paracasei*, *Leptospira terpstrae*, *Phaeospirillum molischianum* and *Weissella oryzae*. Their NCBI accession numbers, VQ motif positions and their sequences were listed in Additional file 4: Table S4. Thus, our data showed that VQs could be detected not only in plants but also in nematodes, fungi and bacteria.

### The *VQ* family in plants could be clustered into 5 distinct groups

**Fig. 3:**
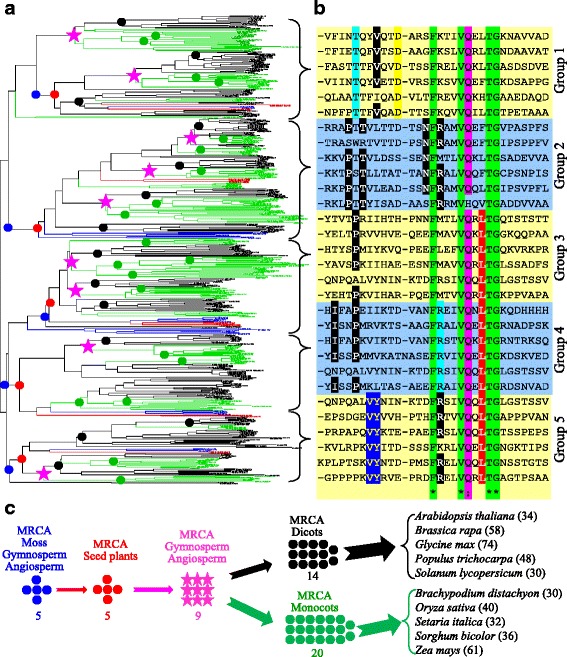
The classification, motif structure, evolutionary dynamics and selection force of the VQ gene family. **a** The phylogenetic analysis and classification of *VQs* among 12 species. The tree was constructed using the bootstrap method with a heuristic search of the PAUP 4.0b10 program as described in the Methods. The tree was verified by Bayesian analyses. Ancestral units were defined according to the description by Shiu et al. (2004) [21]. Black, green, red and blue filled circles represent MRCAs in eudicotyledons, monocotyledons, lycopodiophyta, and bryophyta, respectively. Pink filled stars indicate MRCAs of gymnosperm and angiosperm. Clade lines and VQ locus names from dicots, monocots, pine and moss were highlighted by black, green, red and blue colours, respectively. Enlarged phylogenetic tree is available in Additional file 5: Figure S1. **b** Conserved VQ motif structures of different groups of VQs. The VQ motifs were identified by HMM searched and were confirmed by the Pfam database searches. **c** The evolutionary dynamics of *VQs*. The estimated MRCAs were shown by filled circles/stars and the total numbers of *VQs* were indicated in the brackets following the species names

Previous studies showed that the *VQ* family was classified into 4–9 groups depending on different species and methods used [2, 4, 6, 19]. However, the classification was based on *VQs* from single or several species. Due to the difficulty in sequence alignment, we selected VQ motif sequences from 12 species including 5 monocotyledons (monocots), 5 eudicotyledons (dicots), 1 gymnosperm and 1 moss for phylogenetic analysis (see Methods). Based on the phylogenetic tree, the *VQ* family could be classified into 5 distinct groups and they were named as Group 1, 2, 3, 4 and 5 (Fig. 3a and Additional file 5: Figure S1). These 5 groups of *VQs* were presented in all detected species from angiosperm, gymnosperm and moss. Each group of *VQs* underwent different evolutionary histories and evolved into different subgroups, which were clustered in different species. While all groups showed the common and conserved motif structure FxxhVQxhTG (the conserved residues F, V, Q, T and G were labelled with green fonts in Fig. 3b), each group differentiates from the remaining groups with 1–4 additional conserved residences (Fig. 3b). For example, in Group 1, the residences T (cyan), V (black) and D (yellow) were conserved; however, they might not be conserved in other groups (Fig. 3b). Interestingly, not all VQs contain the conserved residues VQ. Some of *VQs* encode VH instead of VQ, which were detected only in Group 2 of VQs (Fig. 3b). These VQs with conserved VH were presented only in monocot plants and no further evidence is available for why they have evolved into different residence.

### Both monocots and dicots exhibit difference in their expansion histories

As no *VQs* were detected in all tested Chlorophyta species, we surveyed the evolutionary history of the *VQ* family among moss, gymnosperm and angiosperm. The phylogenetic tree was broken down into ancestral units [21] to estimate the most recent common ancestor (MRCA). Due to possible inaccurateness in estimation of lost genes and pseudogenes, they were excluded in this analysis, which might under-estimate the MRCA members. A total of 5 ancestral units were estimated among moss, gymnosperm and angiosperm; thus, their MRCA might encode only 5 *VQs* (blue filled circles in Fig. 3a and c). No expansion occurred before the divergence of seed plants (red filled circles). After the origin of seed plants, nearly double *VQs* (9) were required in the MRCA of gymnosperm and angiosperm (pink filled stars). MRCAs of dicots and monocots required different numbers of *VQs* and they evolved into 14 and 20 *VQs*, respectively, from 9 *VQs*. For dicots, a large-scale of *VQ* expansion was detected mainly during species divergence. However, for monocots, large-scale of *VQ* expansion occurred during the divergence between dicots and monocots followed by species divergence.

### Segmental duplication significantly contributes to the family expansion

**Fig. 4:**
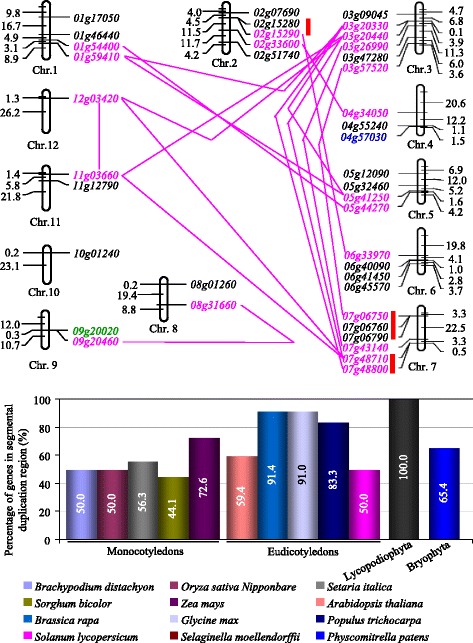
Expansion mechanisms of *VQs*. **a** distribution of *VQs* in rice chromosomes showing their expansion by duplication and mobile elements. Tandemly duplicated genes are indicated with vertical blue bold lines. Segmentally duplicated genes were labelled with pink names and the duplicated pairs were linked with pink lines. The *VQ* gene *04 g57030* labelled with blue font was duplicated by *Pack*-*MULE*. Another *VQ* gene *09 g20020* labelled with green font is a retrogene. The prefix “*LOC_Os*” in each gene locus name was omitted for convenience. **b** Contribution of segmental duplication to the family expansion among 12 species

To explore the possible mechanisms of *VQ* expansion, we first surveyed the contribution of tandem duplication to the family expansion. In *Oryza sativa*, *VQs* were localized on all 12 rice chromosomes with uneven distribution. We detected a total of three tandem clusters covering 7 *VQs* (Fig. 4a), which was also observed in most of other rice species belonging to the genus *Oryza* (Additional file 6: Figure S2). The fact suggested that these tandem duplication events occurred before the divergence of these rice species and some of them lost in some species during long evolutionary history. Interestingly, the tandem duplication could be detected only in the genus *Oryza* but was not in other four grass species. Thus, all these three tandem duplication events occurred after the divergence of rice genus from other grasses but before the presence of various rice species. Furthermore, we have also detected additional three tandem duplication events which occurred only in one species. One was observed on chromosome 1 in the species *O. meridionalis* and another was on chromosome 9 for the species *O. punctate* (Additional file 6: Figure S2). The fact suggests that these duplication events occurred after species divergence from the *Oryza* genus and were species specific and the duplicated *VQs* might take part in species divergence. We then analysed the contribution of mobile elements to the family expansion. We examined the contribution of various mobile elements to *VQ* expansion including *LTR*-retrotransposon, *retrogene*, *MULE*, *CACTA*, and *hobo*/*Ac*/*Tam3* (*hAT*) and *Helitron* elements. We found that one *VQ* gene *04 g57030* (blue fonts in Fig. 4a) was related to one of rice *MULEs* and another *VQ* gene *09 g20020* (green fonts in Fig. 4a) is a retrogene. Thus, limited contribution of mobile elements was detected to the *VQ* family expansion.

As only 9 rice *VQs* were related to tandem duplication or mobile elements, we further investigated the contribution of segmental duplication to the family expansion. We found that a total of 20 *VQs* (50%) were located on segmental duplication fragments (locus names with pink fonts, Fig. 4a). The result suggested the significant contribution of segmental duplication to the family expansion. These *VQs* might be segmentally duplicated in multiple historic periods as some of their orthologs could be detected in not only dicots but also monocots including different rice species; however, some of the orthologs were observed only in the rice species. To survey the contribution of segmental duplication to *VQ* expansion in other species, we also identified segmentally duplicated *VQs* in all 12 species which were used for the phylogenetic tree construction (Fig. 3a). Our data showed that 44.1–100% of *VQs* were located on segmentally duplicated regions in these 12 species (Fig. 4b). The results suggested that segmental duplication should be regarded as the main mechanism for *VQ* gene expansion.

### Limited contribution of gene conversion to the evolution of the *VQ* family

Duplicated genes in a family provide possible DNA fragments for gene conversion, which might contribute to the evolution of the gene family. We implemented the program GENECONV version 1.81 [22] to detect the possible gene conversion events in this gene family. In the rice genome, genome-wide gene conversion events have been reported [23] and no VQ motif coding genes were identified to be involved in the gene conversion events. We implemented the GENECONV program using aligned VQ motif coding sequences from the rice genome and confirmed that no gene conversion events were detected in the rice *VQ* gene family. Additionally, we further detected the gene conversion events in additional 11 species as listed in this study and found that a total of 13 gene conversion events were detected (Additional file 7: Table S5). These gene conversion events were from 5 species including *Brachypodium distachyon* (2 events), *Brassica rapa* (4 events), *Glycine max* (2 events), *Selaginella moellendorffii* (3 events) and *Zea mays* (2 events). The results suggested the limited contribution of gene conversion to the family evolution in these 5 species. For the remaining 6 species, no gene conversion events were detected in the *VQ* gene family.

### Positive selection occurred during species divergence

**Fig. 5:**
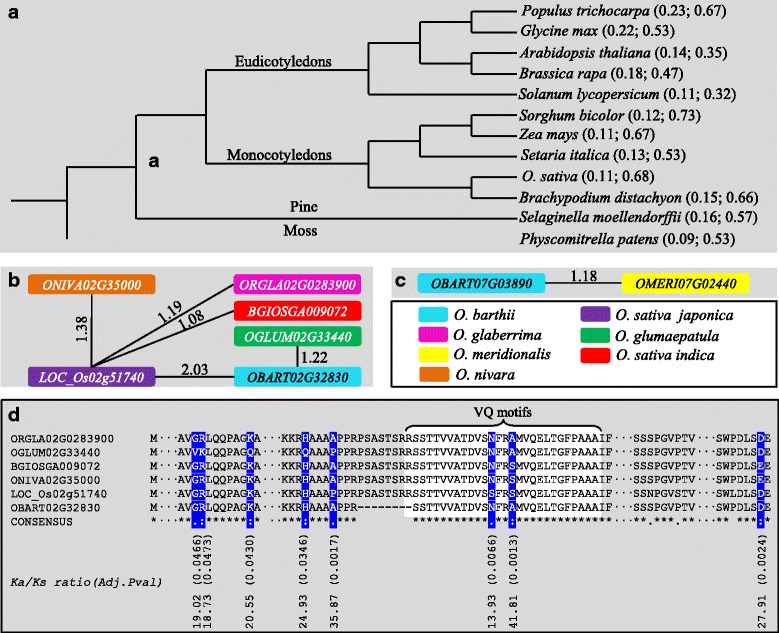
Purifying and positive selection of *VQs* during evolution. **a** The selection force of duplicated *VQs* in 12 species shown by *Ka/Ks* values. The tandem and segmentally duplicated *VQ* pairs were used for *Ka/Ks* calculation. Two *Ka*/*Ks* values were displayed and separated by semicolons. The left values were estimated using *VQ* motif regions and right values were calculated using the full-length *VQs*. The figure showed the average *Ka/Ks* ratios among duplicated *VQ* pairs in each species. The phylogenetic tree was constructed according to the data retrieved from the NCBI Taxonomy database (http://www.ncbi.nlm.nih.gov/taxonomy). **b** and **c** Orthologous genes (shown by locus names) with positive selection among 7 species/subspecies from the *Oryza* genus. These species include *O. barthii*, *O. glumaepatula*, *O. meridionalis*, *O. nivara*, *O. rufipogon*, *O. sativa Indica*, and *O. sativa japonica*. A pair of orthologous genes from two species was linked by a line and the corresponding *Ka/Ks* ratio was labelled near the line. **d** Alignment of amino acid residues from the 5 VQs with positive selection in **b**. The amino acid residues with positive selection were highlighted by blue color. Their *Ka/Ks* ratios were shown under the highlighted residues. VQ motif regions were highlighted by white color. The dots “···” represent the omitted amino acid residues with no variation among 6 species. The asterisk “*” indicates positions with fully conserved residues. The colon “:” and the period “.” indicate conservation between groups of strongly and weakly similar properties of residues, respectively

Our data showed that segmental duplication was a main driver for the family expansion followed by tandem duplication (Fig. 4). Thus, we investigated their selection forces after segmental duplication. Both VQ motif coding region and the full-length *VQs* were separately subjected to nonsynonymous substitutions per site (*Ka*) and synonymous substitutions per site (*Ks*) and their ratio (*Ka/Ks*) evaluation (Fig. 5a). For each species, the *Ka/Ks* ratio at VQ motif coding region was significantly lower than that from the full-length *VQ*, suggesting the functional conservation of VQ motifs during long evolution. For both regions, no *Ka/Ks* ratio was larger than 1, suggesting a purifying selection for these segmentally duplicated genes in all species. We further examined whether these segmentally duplicated genes were under functional constraint by C-value test [24]. Generally, VQ motif coding regions for all segmentally duplicated genes were under functional constraints. However, functional divergence might have occurred when the full-length *VQs* were used for *Ka/Ks* estimation followed by C-value test although they were under purifying selection.

To survey the selection forces of this gene family among species, we calculated the *Ka/Ks* ratios between orthologous *VQ* pairs. We have identified a total of 339 orthologous *VQs* among 10 rice species (Additional file 8: Table S6) that belong to the *Oryza* genus. These form 1169 pairs of orthologous *VQs*. We submitted these pairs of *VQs* for *Ka*/*Ks* analysis. Our analysis showed that most of *Ka/Ks* ratios were less than 1, indicating the purifying selection during and after species divergence. However, we have detected at least 6 pairs of *VQs* (0.5%) showing *Ka*/*Ks* > 1 (Fig. 5b and c). These 6 pairs of *VQs* were from 2 orthologous loci as shown in Fig. 5b and c, respectively. One of them consists of 6 VQ members (Fig. 5b) and another locus contains only two *VQs* (Fig. 5c). For the 6 *VQ* members (Fig. 5b), we further investigated the selection force in each amino acid site using the SLR program (see Methods). Totally, we detected 8 amino acid sites (highlighted by blue colour) with *Ka/Ks* > > 1 with strong statistical supporting (Adj.Pval < 0.05) (Fig. 5d). Two of them were located on VQ motif region. The analysis reveals that strong positive selection occurred not only in non-VQ motif region but also in conserved VQ region. Thus, positive selection might significantly contribute to the family evolution and neofunctionalization as well as species divergence.

### *VQs* were expressed in various tissues and frequently regulated by abiotic / biotic stresses and phytohormones

**Fig. 6:**
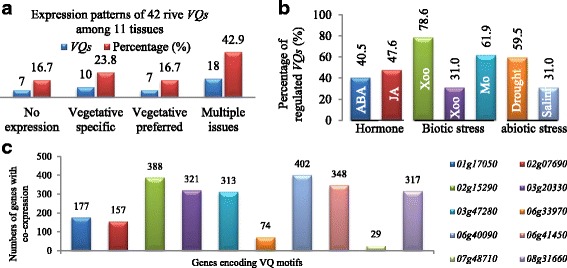
Expression profiling, co-expression and genome-wide identification of protein-protein interactions. **a** Expression patterns of rice *VQs* among 11 different developmental stages of tissues (T1-T11). Vegetative tissues include leaf (20 days, T1), shoot (T2), seedling (four-leaf stage, T3), and pre-emergence inflorescence (T4). T5, Post-emergence inflorescence; T6, Anther; T7, Pistil; T8, Seed (5 DAP); T9, Seed (10 DAP); T10, Embryo (25 DAP); T11, Endosperm (25 DAP). **b** Percentages of *VQs* showing up- or down-regulation by phytohormones, abiotic and biotic stresses. *Xoo*, *Xanthomonas oryzae* pv. *Oryzae*; *Xoc*, *Xanthomonas oryzae* pv. *Oryzicola*; *Mo*, *Magnaporthe oryzae*. **c** A total of 10 *VQs* showing co-expression with their total numbers of co-expressed genes. **d** Genome-wide identification of VQ protein interactions. Interacted proteins with VQs were identified by the STRING program (v10, http://string.embl.de). Red rectangles indicate 16 VQs with interacted proteins. Cyan pies, green triangles indicate WRKY and other transcription factors, respectively. Blue squares and yellow diamonds indicate expressed/hypothetical and other annotated proteins, respectively, which show interactions with VQs. Red lines show the interactions within VQs. Cyan, green, blue and yellow lines show the interactions of VQs with WRKYs, other transcription factors, expressed / hypothetical proteins and other annotated proteins, respectively. The prefix “*LOC_Os*” in each gene locus name in **c** and **d** was omitted for convenience

Among 42 rice *VQs*, 7 of them (16.7%) showed no detectable expression (Fig. 6a and Additional file 9: Figure S3a). The remaining 35 *VQs* were expressed in either one or multiple tissues. Among them 10 genes (23.8%) were expressed only in vegetative tissue (T1 to T4). Statistical analysis showed that other 7 genes out of the 35 rice *VQs* were preferentially expressed in vegetative tissues. Their average express abundance in four vegetative tissues (T1-T4) was at least two times’ higher than the expression level in T5 to T11. The remaining 18 *VQs* showed expression signals in multiple tissues. Generally, around half of the rice *VQs* were preferentially expressed in vegetative tissues. On the contrary, higher percentage of *Arabidopsis VQs* showed reproductive tissue preferred expression patterns (Additional file 10: Figure S4a).

Expression data also showed that rice *VQs* were frequently regulated by phytohormones, biotic and abiotic stresses (Fig. 6b, Additional file 9: Figure S3b-d). Under ABA treatment, 40.5% of rice *VQs* were up- or down-regulated. Similarly, 47.6% of *VQs* were regulated by JA. Under both bacterial and fungus pathogen treatments, 31–78.6 of *VQs* were up- or down regulated. Under drought and high salinity stresses, 59.5 and 31% of rice *VQs* were either down- or up-regulated, respectively. In *Arabidopsis*, *VQs* were frequently regulated under JA treatment. However, less *VQs* were down- or up-regulated either by other phytohormones or under various abiotic and biotic stresses in *Arabidopsis* (Additional file 10: Figure S4b).

### Some of *VQs* were co-expressed with abiotic and biotic stress-related genes

We identified 10 out of 40 *VQs* with co-expression modules in at least 3 out 14 datasets. A total of 2526 genes were identified to co-express with these 10 *VQs* (Fig. 6c). The *VQ* gene *07 g48710* was co-expressed with only 29 genes while a total of 403 genes were found to co-express with *06 g41450*. Co-expressed genes with each of these 10 *VQs* were separately submitted to rice Gene Ontology (GO) database for gene set enrichment analysis (GSEA; [25]). No over-represented genes were detected for two *VQs 02 g07690* and *07 g48710*. For the remaining 8 *VQs*, 3 *VQs* were detected with over-represented cellular component (C) (Additional file 11: Figure S5a). All 8 *VQs* were found with over-represented molecular function (F) (Additional file 11: Figure S5a). Interestingly, most of over-represented biological processes are responses to biotic or abiotic stress / stimulus, suggesting the roles of *VQs* in stress-related signalling pathways.

We are interested in co-expressed genes encoding transcription factors. We have detected 136 co-expressed genes encoding 20 families of transcription factors (Additional file 11: Figure S5b). For most of the families, less than 10 family members were shown to co-express with *VQs*. We detected a total of 5 transcription factor families, which consist of at least 15 co-expressed genes (Additional file 11: Figure S5b). These candidate transcription factors will be investigated in their interaction with *VQs* (see below).

### Expression profiling of *VQs* from nematodes and fungi

**Fig. 7:**
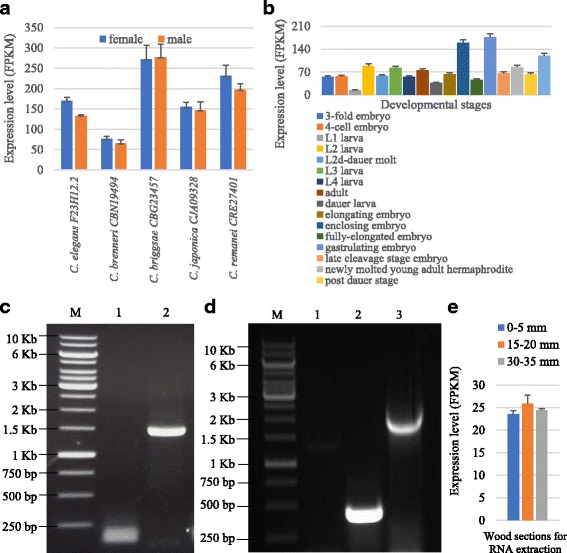
Expression analysis of some of *VQs* from nematode and fungus. **a** Expression profiling of VQs from 5 nematode species. The RNA-Seq data were achieved from https://www.ebi.ac.uk/gxa/experiments/E-MTAB-2812/. **b** Expression profiling of the VQ gene F23H12.2 in *C. elegans* among 17 different developmental stages of tissues. **c** The genomic DNA sample was isolated from the fungus *Gloeophyllum trabeum* and was used as the template to isolate the gene with the NCBI accession number XM_007869238.1 by PCR amplification. The used primer sequences were listed in Additional file 1: Table S1. M, marker (1 Kb Ladder); 1, PCR with no template (negative control); 2, with the fungus genomic DNA as template. **d** Expression analysis of the gene with the NCBI accession number XM_007869238.1 by RT-PCR. M, marker (1 Kb Ladder); 1, RT-PCR with no template (negative control); 2 and 3, with the fungus total RNA as template using Actin primers (positive control) and XM_007869238.1 primers, respectively. **e** Expression profiling of the *VQ* gene *XM*_*007869238.1* from *Gloeophyllum trabeum*. The RNA-Seq data in (**b**) and (**e**) were achieved from the NCBI GEO Datasets with accession numbers GSE41367 and GSE108189, respectively. The expression abundance was shown by FPKM (Fragments Per Kilobase Million) values in (**a**), (**b**) and (**e**)

Previously, no VQ motif coding genes was identified in nematodes and fungi. Here, we identified 14 nematode and 34 fungus *VQs*. These 14 nematode VQs were from 11 species and publicly available RNA-Seq expression data were collected from 5 species including *Caenorhabditis brenneri*, *Caenorhabditis briggsae*, *Caenorhabditis elegans*, *Caenorhabditis japonica* and *Caenorhabditis remanei* (Fig. 7a). RNA-Seq analysis showed that all these 5 *VQs* were expressed in either male or female with the lowest expression level in *C. brenneri* and the highest expression level in *C. briggsae* (Fig. 7a). In *C. elegans*, more expression data were available and we analysed the expression profiling of the *VQ* gene *F23H12.2* among 17 different developmental stages of tissues (Fig. 7b). The analysis showed that the *VQ* gene was expressed in all tested tissues with varying expression abundance (Fig. 7b). The lowest expression level was detected the L1 larva and the highest abundance was detected in gastrulating embryo.

On the other hand, we have identified 34 *VQs* in fungi and all of them were annotated proteins. To verify the annotation, one of the fungus *VQs* were selected, which was from *Gloeophyllum trabeum* with the NCBI accession number XM_007869238.1. The genomic DNA was isolated from the species and was used as template to amplify the fragment by PCR. The PCR result (Fig. 7c) and sequencing data confirmed that the annotated gene was from the genome and its deduced protein sequence indeed contained the VQ motif structure. RT-PCR analysis showed that the full-length coding region was transcribed in the fungus strain (Fig. 7d). In addition, we further analysed the expression profiling by using RNA-Seq data. The specie was used to colonize wood wafer and 3 wood sections (0–5, 15–20, 30–35 mm) were sampled for RNA-Seq, representing early to late decay stages. The *VQ* gene was expressed in all 3 wood sections with similar expression abundance (Fig. 7e). All the experiments not only verified the annotated *VQ* gene but also provided its expression evidence, which further confirmed its presence in the genome.

## Discussion

### The ancient origin and evolution of the *VQ* gene family

Among the 50 genomes used in this study, 7 genomes from Chlorophyte encode no *VQs*. The conserved VQ motif coding sequences were also lacking in additional 12 algae genomes. Thus, our data showed that the *VQ* gene family ubiquitously exists in most of Viridiplantae species but might be excluded in Chlorophyte. To explore the possible presence in other genomes, the constructed HMM profile was also used for genome-wide searches against many other protein databases from Uniprot (https://www.uniprot.org//), Swissprot (https://www.ebi.ac.uk/uniprot/), Protein Data Bank (PDB; https://www.rcsb.org/) and Ensemble database (https://www.ensembl.org/). Totally, we have genome-widely identified the *VQ* family members in 43 nematode, 249 fungus and 50 bacterial genomes and identified 14, 34 and 8 *VQs* in nematodes and fungi, respectively (Fig. 2). Some of these *VQs* were verified by either sequencing or expression (Fig. 7). Many detected *VQs* in nematodes, fungi and bacteria were not intact with only partial VQ motif sequence or its variations. For example, in nematodes *Globodera pallida* and *Caenorhabditis elegans*, VQ motifs were also detected but the two conserved amino acids “TG” were not presented. A similar situation was observed in other nematodes and some fungi as well as other two lower animals *Macrostomum lignano* (flatworm) and *Giardia intestinalis*. The former encodes a protein with 6 VQ motifs (Uniprot ID: A0A1I8HNL6) and the latter has a protein with 2 VQ motifs (accession number: XP_001709138.1) but all of them contain no “TG”. We further examined the presence of “TG” in higher plants and some variations for the residue “G” were also found in a few of plant VQs. The variations include “TR” in soybean GLYMA03G36330, “TA” in tomato Solyc02g064570 and “TS” in Solyc12g042730. Generally, *VQs* have been mainly detected in moss, gymnosperm and angiosperm. These species encode at least 10 but less than 80 *VQs*. They could also be found in unicellular lower animals, fungi and bacteria. These species encode only one to several *VQs* and their VQ motifs may not be intact. The presence of *VQs* in these lower animals, fungi and bacteria suggest an ancient origin of this gene family. Different from *VQs*, *WRKYs* were detected in Chlorophyte but not in lower animals and fungi [25]. Furthermore, obvious differences were observed in the expansion patterns and mechanisms between *VQs* and *WRKYs*. For example, rice *VQs* were mainly expanded by segmental duplication (Fig. 4); however, up to 35% of rice *WRKYs* were expanded by tandem duplication [26]. Thus, despite of their universal interactions between VQs and WRKYs, these two families should have evolved independently with different origins, evolutionary histories and patterns.

### *VQs* play important roles in abiotic and biotic stress signalling pathway

Various reports showed that relatively higher ratios of *VQs* were down- or up-regulated by abiotic and biotic stresses as well as under phytohormone treatments. We carried out a comprehensive survey and comparative analysis on rice and *Arabidopsis VQs*. We found that higher ratio of rice *VQs* were up- or down-regulated by stresses or JA treatment when compared those from *Arabidopsis* (Fig. 6b and Additional file 9: Figure S3). However, for JA treatment, up to 74% of *Arabidopsis VQs* were down- or up-regulated while only 47.6% of rice *VQs* showed JA-regulated expression patterns (Fig. 6b and Additional file 10: Figure S4). By combining all treatments, we found that all tested genes were up- or down-regulated by either phytohormes or abitic/biotic stress in rice (Fig. 6b and Additional file 9: Figure S3b-d). However, in *Arabidopsis*, two genes were not regulated by any of tested phytohormone or abiotic/biotic stresses. On the other hand, 16 rice *VQs* were regulated only by abiotic/biotic stresses but not either ABA or JA, suggesting that these genes might function in a phytohormone-independent signalling pathway. Interestingly, all ABA/JA-regulated rice *VQs* were also down- or up-regulated by ether abiotic or biotic stresses. This result suggested the role of ABA/JA signalling in abiotic/biotic stress tolerance. However, in *Arabidopsis*, some of ABA−/JA-regulated genes were not regulated by abiotic/biotic stresses. These *Arabidopsis VQs* might be regulated by other abiotic/biotic stresses or play roles only in phytohormone-mediated non-stress-related biological processing. All of these analyses imply the difference in their biological functions for individual *VQs* between rice and *Arabidopsis*.

## Conclusions

By implementing an integrative approach, we provide new insights into gene expansion, divergence, evolution and their interaction networks of this VQ family. Our data showed that all genomes from moss, gymnosperm and angiosperm encode varying numbers of *VQs* and segmental duplication significantly contributed to the family expansion. VQs were very much conserved in their motif coding region and were under purifying selection although positive selection was also observed during species divergence. We also detected the presence of this family in other genomes from alga, animals, bacteria, fungi and viruses, which showed that no *VQs* were detected in all tested algae and virus genomes but VQs could be encoded by some of fungi, bacteria and lower animals. All of these data suggest that VQs are evolutionary conserved, ancient and not plant-specific. Many *VQs* were up- or down-regulated by various abiotic / biotic stresses and phytohormones in rice and *Arabidopsis*. They were also co-expressed with some of other stress-related genes. Thus, our data suggest the comprehensive expression regulation of this *VQ* gene family.

## Methods

### Materials and growth conditions

The fungus *Gloeophyllum trabeum* was a gift from the U.S. Department of Agriculture Forest Products Laboratory (Madison, WI) and was routinely maintained on 2.5% malt agar plates.

### Genomic DNA and RNA isolation and gene cloning

The fungus genomic DNA was isolated using Quick-DNA Fungal/Bacterial Miniprep Kit (50 Preps, ZYR.D6005), Zymo Research, USA. Around 500 ng of genomic DNA was used as template for PCR amplification and verification of an annotated VQ motif encoding gene with accession number XM_007869238.1 using the primers as listed in Additional file 12: Table S7. PCR products were separated on a 1.0% of agarose gel by electrophoresis and were then purified by QIAquick® Gel Extraction Kit (Qiagen) for sequencing and cloning.

RNA samples were isolated by using RNeasy Mini Kit from Qiagen. Around 500 ng of total RNA for each sample was used as template for RT-PCR analysis to amplify corresponding coding regions using the QIAGEN OneStep RT-PCR Kit. All primer sequences used in this study were listed in Additional file 12: Table S7. RT-PCR products were separated by 1.0% agarose gel for visualisation.

### DNA and protein databases used

All protein and coding sequences from 50 species were downloaded from the v12 Phytozome database (https://phytozome.jgi.doe.gov/). For the 9 *Oryza* species, *Oryza rufipogon*, *Oryza glaberrima*, *Oryza brachyantha*, *Oryza barthii, Oryza sativa indica, Oryza punctata, Oryza meridionalis, Oryza nivara, Oryza glumaepatula,* their protein and coding sequences were downloaded from the Gramene database (release52, ftp://ftp.gramene.org/pub/gramene/archives/PAST_RELEASES/release52/). For the screening of *VQs* in wide ranges of species including bacteria, protists, fungi, plants and metazoan, their protein databases were downloaded from the EnsembleGenomes database (http://ensemblgenomes.org/info/access/ftp). For the nematode genomes, their protein sequences were downloaded from either NCBI (https://www.ncbi.nlm.nih.gov/genome/) or WormBase (ftp.wormbase.org). Species names for plants, nematodes and fungi have been listed in Additional file 1: Table S1 and Additional file 3: Table S3.

### Genome-wide identification of *VQs* by HMM and BLAST searches

All *VQs* encode a conserved VQ motif with Pfam ID PF05678 (http://pfam.xfam.org/family/PF05678#tabview=tab3). The 119 seed VQ motif sequences were downloaded from the Pfam database, which were aligned using Clustal X2 (http://www.clustal.org/clustal2/). The aligned VQ motifs were used to construct the HMM profile using the HMMER v3.1b2 (windows version, http://www.hmmer.org/). The HMM searches were carried out using the order “hmmsearch” under the E-value cutoff at 0.01 against all protein databases. The identification of VQs was also carried out by BLASTP searches using the 119 seed VQ motif sequences as queries at the E-value cutoff at 0.01. All candidates from both HMM and BLASTP searches were then submitted to the “Motif Search” database (http://www.genome.jp/tools/motif/) to confirm the presence of the VQ motif.

### Phylogenetic tree and *VQ* classification

VQ motif sequences from 12 plant species were aligned by the Clustal X2 program (http://www.clustal.org/clustal2/). The aligned VQ motif sequences, which were listed in Additional file 13, were employed for phylogenetic tree construction using the program Mac PAUP 4.0b10 (ppc) (http://paup.phylosolutions.com//) by both Maximum parsimony (MP) and maximum-likelihood (ML) analyses. Bootstrap supports of specific nodes were estimated with 1000 replicates with default options in the PAUP program. The MrBayes 3.2.6 program (http://mrbayes.csit.fsu.edu/) was used for Bayesian searches with the WAG evolutionary model [27].

### Detection of expansion mechanisms of *VQs* in 12 plant species

Tandemly duplicated *VQs* should meet the following criteria (1) *VQs* should be located within ten array genes; (2) they should within 100 kb genome region for *Arabidopsis* and moss or 350 kb for the remaining 10 species. Sequence regions flanking 50-kb upstream and downstream of a *VQ* were used for the identification of segmentally duplicated *VQs* or expanded *VQs* by various mobile elements including *mutator-like transposable element* (*MULE*), *hobo*/*Ac*/ *Tam3* (*hAT*), *CACTA*, retrotransposons and *Helitron* families as well as retroposed genes (*retrogenes*) as described by Jiang et al. (2009) [28]. *VQs* that were located on a mobile element were regarded as a mobile element related *VQs*.

### *Ka* and *Ks* estimation and detection of positive/purifying selection

*Ka*, *Ks* and their ratios were calculated using a pair of duplicated genes within a species or orthologous genes between species. Duplicated genes were identified as shown above. Orthologous genes among 10 rice species from the *Oryza* genus were identified using the Ensembl Plants database (http://plants.ensembl.org/). For each pair of duplicated or orthologous genes, full-length or VQ motif amino acid sequences were aligned using the Smith-Waterman alignment program [29]. The aligned protein sequences were converted into corresponding cDNA alignment which was used for *Ka*, *Ks* and *Ka*/*Ks* estimation with the KaKs_Calculator [30]. To estimate purifying/positively selected amino acid sites in a group of orthologous *VQs*, their full-length amino acid sequences were aligned first and were then subjected to the “sitewise likelihood-ratio” (SLR) program (http://www.ebi.ac.uk/goldman-srv/SLR/) to detect the *Ka/Ks* ratio in each amino acid site. The positive selection was identified by statistical analysis using an adjusted *P* value (Adj.Pval) from multiple comparisons as suggested by the SLR program.

### Detection of gene conversion events

To identify the possible gene conversion, VQ motif sequences from each species were first aligned with Clustal X2 (http://www.clustal.org/clustal2/) and were then converted into corresponding coding sequences. The aligned coding sequences were used for the program GENECONV version 1.81 [22] to detect the possible gene conversion events using the default parameters. *P*-values from global fragments were used to evaluate the gene conversion events as suggested by Mondragon-Palomino et al. (2005) [31] and Xu et al. (2008) [23]. The global *P*-values <= 0.05 for inner fragments were considered as statistical significance requirement for gene conversion events.

### Expression databases, gene expression profiling and co-expression

Rice RNA-Seq raw expression data from 11 different tissues including T1, leaf (20 days); T2, shoot; T3, seedling (four-leaf stage); T4, pre-emergence inflorescence; T5, post-emergence inflorescence; T6, anther; T7, pistil; T8, seed (5 days after pollination, DAP); T9, seed (10 DAP); T10, embryo (25 DAP); T11, endosperm (25 DAP) were achieved from NCBI Sequence Read Archive (SRA, https://www.ncbi.nlm.nih.gov/sra) with accession number SRP008821. In *Arabidopsis*, expression data were achieved from NCBI GEO data sets under the accession numbers: GSE5633 (tissues T1-T14) and GSE5634 (T15-T22), GSE61884 (cold, drought, NaCl and ABA), GSE61884 (Jasmonic acid, JA), GSE40973 (*Golovinomyces orontii*) and GSE92631 (*Ralstonia solanacearum*). A tissue-specific gene was identified when the gene showed expression signal only in the tissue(s) and no signal was detected in the remaining tissues. A tissue-preferred gene was identified when the expression abundance in the tissue(s) of this gene was statistically at least two times’ higher than that in any of the remaining tissues. The method was also employed to identify up- or down-regulated genes under biotic / abiotic stresses or under phytohormone treatments. Similarly, expression divergence among expanded genes was determined using their expression abundance among different tissues or expression regulation under various abiotic / biotic stresses or under phytohormone treatment.

For co-expression analysis, a total of 14 rice expression datasets were selected and their NCBI GEO accession numbers were listed as below: E-MEXP-1766, E-MEXP-2267, E-MEXP-2506, GSE10373, GSE11025, GSE16793, GSE17245, GSE18361, GSE19024, GSE19239, GSE4471, GSE6719, GSE6893, and GSE6901. Co-expression modules were identified using the WGCNA method as described by Zhang and Horvath (2005) [32]. A co-expression gene pair was identified when they showed in at least 3 expression datasets with positive or negative correlation coefficient > =0.8 or < = − 0.8.

## Additional files

Additional file 1: **Table S1.** The basic information of 50 completely sequenced genomes and related publications. (PDF 26 kb)

Additional file 2: **Table S2.** Genome-wide identification of the VQ gene family in 50 completely sequenced genomes. (PDF 593 kb)

Additional file 3: **Table S3.** Nematode and fungus species used for the genome-wide identification of *VQs. (PDF 107 kb)*

Additional file 4: **Table S4.** Identification of *VQs* in some of bacterial species. (PDF 13 kb)

Additional file 5: **Figure S1.** The enlarged phylogenetic tree of Fig. 2a. (PDF 717 kb)

Additional file 6: **Figure S2.** Syntenic analysis of orthologous *VQs* among ten species of the *Oryza* genus. (PDF 218 kb)

Additional file 7: **Table S5.** Identification of gene conversion events in the VQ motif family. (PDF 15 kb)

Additional file 8: **Table S6.** The *VQ* gene family members and their coordinates among 10 species from the *Oryza* genus. (PDF 49 kb)

Additional file 9: **Figure S3.** Expression profiling of rice *VQs* among various tissues and under abiotic and biotic stresses. (PDF 144 kb)

Additional file 10: **Figure S4.** Expression profiling of *Arabidopsis VQs* among different tissues and under various abiotic / biotic stresses and hormones. (PDF 75 kb)

Additional file 11: **Figure S5.** Gene set enrichment analysis of co-expressed genes with *VQs. (PDF 128 kb)*

Additional file 12: **Table S7.** Primer sequences used in this study. (PDF 9 kb)

Additional file 13: The alignment of VQ motif sequences from 12 plant species. (PDF 106 kb)

## Abbreviations

AD: Activation domain
AP2/EREBP: Apetala 2/ ethylene-responsive element binding protein
BD: Binding domain
bHLH: Basic helix-loop-helix
DAP: Days after pollination
hAT: Hobo/Ac/ Tam3
HMM: Hidden markov model
*Ka*: Nonsynonymous substitutions per site
*Ks*: Synonymous substitutions per site
MRCA: Most recent common ancestor
MULE: Mutator like transposable element (MULE)
MYB: Myeloblastosis
NAC: NAM, ATAF and CUC
SLR: Sitewise likelihood-ratio
VQ motif: Valine-glutamine motif
ZIMs: A plant-specific GATA factor
ZINCs: Zinc finger transcription factor

## References

[1] Morikawa K, Shiina T, Murakami S, Toyoshima Y (2002). Novel nuclearencoded proteins interacting with a plastid sigma factor, Sig1, in *Arabidopsis thaliana*. FEBS Lett.

[2] Cheng Y, Zhou Y, Yang Y, Chi YJ, Zhou J, Chen JY (2012). Structural and functional analysis of VQ motif containing proteins in *Arabidopsis* as interacting proteins of WRKY transcription factors. Plant Physiol.

[3] Jing Y, Lin R (2015). The VQ motif-containing protein family of plant-specific transcriptional regulators. Plant Physiol.

[4] Zhang G, Wang F, Li J, Ding Q, Zhang Y, Li H (2015). Genome-wide identification and analysis of the VQ motif-containing protein family in Chinese cabbage (*Brassica rapa* L. ssp. Pekinensis). Int J Mol Sci.

[5] Kim DY, Kwon SI, Choi C, Lee H, Ahn I, Park SR (2013). Expression analysis of rice *VQ* genes in response to biotic and abiotic stresses. Gene.

[6] Song W, Zhao H, Zhang X, Lei L, Lai J (2016). Genome-wide identification of VQ motif-containing proteins and their expression profiles under abiotic stresses in maize. Front Plant Sci.

[7] Jiang Y, Yu D (2016). The WRKY57 transcription factor affects the expression of jasmonate ZIM-domain genes transcriptionally to compromise *Botrytis cinerea* resistance. Plant Physiol.

[8] Li N, Li X, Xiao J, Wang S (2014). Comprehensive analysis of VQ motif-containing gene expression in rice defense responses to three pathogens. Plant Cell Rep.

[9] Wang H, Hu Y, Pan J, Yu D (2015). *Arabidopsis* VQ motif-containing proteins VQ12 and VQ29 negatively modulate basal defense against *Botrytis cinerea*. Sci Rep.

[10] Andreasson E, Jenkins T, Brodersen P, Thorgrimsen S, Petersen NH, Zhu S (2005). The MAP kinase substrate MKS1 is a regulator of plant defense responses. EMBO J.

[11] Hu P, Zhou W, Cheng Z, Fan M, Wang L, Xie D (2013). JAV1 controls jasmonate-regulated plant defense. Mol Cell.

[12] Lai Z, Li Y, Wang F, Cheng Y, Fan B, Yu JQ (2011). *Arabidopsis* sigma factor binding proteins are activators of the WRKY33 transcription factor in plant defense. Plant Cell.

[13] Pecher P, Eschen-Lippold L, Herklotz S, Kuhle K, Naumann K, Bethke G (2014). The *Arabidopsis thaliana* mitogen-activated protein kinases MPK3 and MPK6 target a subclass of ‘VQ-motif’-containing proteins to regulate immune responses. New Phytol.

[14] Petersen K, Qiu JL, Lütje J, Fiil BK, Hansen S, Mundy J, Petersen M (2010). *Arabidopsis* MKS1 is involved in basal immunity and requires an intact N-terminal domain for proper function. PLoS One.

[15] Xie YD, Li W, Guo D, Dong J, Zhang Q, Fu Y (2010). The *Arabidopsis* gene SIGMA FACTOR-BINDING PROTEIN 1 plays a role in the salicylate- and jasmonate-mediated defence responses. Plant Cell Environ.

[16] Chi Y, Yang Y, Zhou Y, Zhou J, Fan B, Yu JQ (2013). Protein-protein interactions in the regulation of WRKY transcription factors. Mol Plant.

[17] Hu Y, Chen L, Wang H, Zhang L, Wang F, Yu D (2013). *Arabidopsis* transcription factor WRKY8 functions antagonistically with its interacting partner VQ9 to modulate salinity stress tolerance. Plant J.

[18] Wang A, Garcia D, Zhang H, Feng K, Chaudhury A, Berger F (2010). The VQ motif protein IKU1 regulates endosperm growth and seed size in *Arabidopsis*. Plant J.

[19] Zhou Y, Yang Y, Zhou X, Chi Y, Fan B, Chen Z (2016). Structural and functional characterization of the VQ protein family and VQ protein variants from soybean. Sci Rep.

[20] Wang M, Vannozzi A, Wang G, Zhong Y, Corso M, Cavallini E (2015). A comprehensive survey of the grapevine *VQ* gene family and its transcriptional correlation with WRKY proteins. Front Plant Sci.

[21] Shiu SH, Karlowski WM, Pan R, Tzeng YH, Mayer KF, Li WH (2004). Comparative analysis of the receptor-like kinase family in *Arabidopsis* and rice. Plant Cell.

[22] Sawyer SA (1989). Statistical tests for detecting gene conversion. Mol Biol Evol.

[23] Xu S, Clark T, Zheng H, Vang S, Li R, Wong GK, Wang J, Zheng X (2008). Gene conversion in the rice genome. BMC Genomics.

[24] Betran E, Thornton K, Long M (2002). Retroposed new genes out of the X in *Drosophila*. Genome Res.

[25] Zhang Y, Wang L (2005). The WRKY transcription factor superfamily: its origin in eukaryotes and expansion in plants. BMC Evol Biol.

[26] Ramamoorthy R, Jiang SY, Kumar N, Venkatesh PN, Ramachandran S (2008). A comprehensive transcriptional profiling of the *WRKY* gene family in rice under various abiotic and phytohormone treatments. Plant Cell Physiol.

[27] Whelan S, Goldman N (2001). A general empirical model of protein evolution derived from multiple protein families using a maximum-likelihood approach. Mol Biol Evol.

[28] Jiang SY, Christoffels A, Ramamoorthy R, Ramachandran S (2009). Expansion mechanisms and functional annotations of hypothetical genes in the rice genome. Plant Physiol.

[29] Smith TF, Waterman MS (1981). Identification of common molecular subsequences. J Mol Biol.

[30] Wang D, Zhang Y, Zhang Z, Zhu J, Yu J (2010). KaKs_Calculator 2.0: a toolkit incorporating gamma-series methods and sliding window strategies. Genomics Proteomics Bioinformatics.

[31] Mondragon-Palomino M, Gaut BS (2005). Gene conversion and the evolution of three leucine-rich repeat gene families in *Arabidopsis thaliana*. Mol Biol Evol.

[32] Zhang B, Horvath S (2005). A general framework for weighted gene co-expression network analysis. Stat Appl Genet Mol Biol.

