# Supplementary material for: Valine-glutamine (VQ) motif coding genes are ancient and non-plant-specific with comprehensive expression regulation by various biotic and abiotic stresses
Source: BMC Genomics. 2018 May 9;19:342. doi: 10.1186/s12864-018-4733-7 (PMC5941492; doi:10.1186/s12864-018-4733-7)
Supplement: Supplementary file 1 — Table S1. The basic information of 50 completely sequenced genomes and related publications. (PDF 26 kb) [file 12864_2018_4733_MOESM1_ESM.pdf]

Additional file 1: Table S1. The basic information of 50 completely sequenced genomes and related publications

Species	Genome Size (Mb)	Annotated protein (	Publication for genome sequencing or annotation
Amaranthus hypochondriacus	377	23,038	Plant Genome. 2016 doi: 10.3835/plantgenome2015.07.0062.
Amborella trichopoda	748	26,846	Science (New York, N.Y.). 2013 Dec 20; 342 6165 1241089
Ananas comosus	382	27,024	Nature genetics. 2015 Dec ; 47 12 1435-42
Arabidopsis lyrata	207	32,670	Nature genetics. 2011 May ; 43 5 476-81
Arabidopsis thaliana	135	27,416	Nucleic Acids Res.36: D1009-14
Brachypodium distachyon	272	26,552	Nature 463, 763-768
Brassica oleracea	630	35,400	Nature communications. 2014 May 23; 5 3930
Brassica rapa	284	26,374	Nat Genet. 2011 Aug 28; 43(10):1035-9
Capsella grandiflora	112.3	24,805	Nature genetics. 2013 Jul ; 45 7 831-5
Capsella rubella	134.8	26,521	Nature genetics. 2013 Jul ; 45 7 831-5
Carica papaya	135	27,332	Nature. 2008 Apr 24; 452 7190 991-6
Chlamydomonas reinhardtii	111.1	17,741	Science (New York, N.Y.). 2007 Oct 12; 318 5848 245-50
Citrus clementina	301.4	24,533	Nature biotechnology. 2014 Jul ; 32 7 656-62
Citrus sinensis	319	25,376	Nature biotechnology. 2014 Jul ; 32 7 656-62
Coccomyxa subellipsoidea C-169	49	9,629	Genome biology. 2012 May 25; 13 5 R39
Cucumis sativus	203	21,491	Nature Genetics 41, 1275 - 1281
Daucus carota	421	32,113	Nature genetics. 2016 Jun ; 48 6 657-66
Eucalyptus grandis	691	36,349	Nature. 2014 Jun 19; 510 7505 356-62
Eutrema salsugineum	243.1	26,351	Frontiers in plant science. 2013 ; 4 46
Fragaria vesca	240	32,831	Nature genetics. 2011 Feb ; 43 2 109-16
Glycine max	975	65,153	Nature 463, 178-183
Gossypium raimondii	761.4	37,505	Nature. 2012 Dec 20; 492 7429 423-7
Linum usitatissimum	318.3	43,471	The Plant journal : for cell and molecular biology. 2012 Nov ; 72 3 461-73
Malus domestica	881	26374	Nat. Genet. 42: 833-9
Manihot esculenta	582.25	33,033	Nature biotechnology. 2016 May ; 34 5 562-70
Medicago truncatula	390	50,894	Nature. 2011 Nov 16; 480 7378 520-4
Micromonas pusilla CCMP1545	22	10,660	Science (New York, N.Y.). 2009 Apr 10; 324 5924 268-72
Micromonas sp. RCC299	20.9	10,056	Science. 2009 Apr 10;324(5924):268-72. doi: 10.1126/science.1167222.
Mimulus guttatus	312.7	28,140	Proc Natl Acad Sci USA 110: 19478-19482.
Musa acuminata	472	36,528	Database : the journal of biological databases and curation. 2013 ; 2013 bat035
Oropetium thomaeum	250	28,446	Nature. 2015 Nov 26; 527 7579 508-11
Oryza sativa	373	55986*	Nucleic Acids Res. 35: D883-7
Ostreococcus lucimarinus	13.2	7,796	Proc Natl Acad Sci USA 2007 104: 7705-7710
Physcomitrella patens	480	32,272	Science 319: 64-9
Populus trichocarpa	403	40,668	Science 313: 1596-604

Additional file 1: Table S1. The basic information of 50 completely sequenced genomes and related publications (to be continued)

Species	Genome Size (Mb)	Annotated protein [illegible]	Publication for genome sequencing or annotation
Prunus persica	225.7	26,873	Nature genetics. 2013 May ; 45 5 487-94
Ricinius communis	400	31,221	Nat. Biotechnol. 28: 951-956
Selaginella moellendorffii	213	22,273	Science 332: 960-3
Setaria italica	405.7	34,584	Nature biotechnology. 2012 May 13; 30 6 555-61
Solanum lycopersicum	950	34,675	Nature 485:635-41
Solanum tuberosum	~800	35,119	Nature. 2011 Jul 10; 475 7355 189-95
Sorghum bicolor	698	34,496	Nature 457, 551-556
Spirodela polyrhiza	158	19,623	Nature communications. 2014 ; 5 3311
Theobroma cacao	346	29,452	Genome biology. 2013 Jun 03; 14 6 r53
Trifolium pratense	309	40,868	Scientific reports. 2015 Nov 30; 5 17394
Triticum aestivum	1700	99,386	Science (New York, N.Y.). 2014 Jul 18; 345 6194 1251788
Vitis vinifera	487	26,346	Nature 449: 463-7
Volvox carteri	131.2	14,247	Science (New York, N.Y.). 2010 Jul 09; 329 5988 223-6
Zea mays	2300	32540	Science 326: 1112-5
Zostera marina	202.3	20,450	Nature. 2016 Feb 18; 530 7590 331-5
