# Supplementary material for: Valine-glutamine (VQ) motif coding genes are ancient and non-plant-specific with comprehensive expression regulation by various biotic and abiotic stresses
Source: BMC Genomics. 2018 May 9;19:342. doi: 10.1186/s12864-018-4733-7 (PMC5941492; doi:10.1186/s12864-018-4733-7)
Supplement: Supplementary file 2 — Table S2. Genome-wide identification of the VQ gene family in 50 completely sequenced genomes. (PDF 593 kb) [file 12864_2018_4733_MOESM2_ESM.pdf]

Table S2

Additional file 2: Table S2. Genome-wide identification of the VQ gene family in 50 completely sequenced genomes*

Locus name	Species name	Chromosome/Linkage	From	To
407942	*Selaginella moellendorffii*	scaffold_7	3666862	3668456
409496	*Selaginella moellendorffii*	scaffold_10	2396688	2397635
410083	*Selaginella moellendorffii*	scaffold_11	2788359	2789447
414696	*Selaginella moellendorffii*	scaffold_24	1578752	1579477
419311	*Selaginella moellendorffii*	scaffold_42	142154	143146
443192	*Selaginella moellendorffii*	scaffold_30	1742368	1744484
447774	*Selaginella moellendorffii*	scaffold_100	199704	202427
27985.t000012	*Ricinius communis*	27985	69485	70249
28745.t000001	*Ricinius communis*	28745	10308	12319
29333.t000052	*Ricinius communis*	29333	395548	395976
29613.t000002	*Ricinius communis*	29613	15315	15860
29661.t000045	*Ricinius communis*	29661	389081	389680
29668.t000018	*Ricinius communis*	29668	266202	266963
29680.t000008	*Ricinius communis*	29680	55111	55512
29680.t000009	*Ricinius communis*	29680	57670	58080
29693.t000042	*Ricinius communis*	29693	341606	341962
29733.t000029	*Ricinius communis*	29733	639099	640132
29739.t000027	*Ricinius communis*	29739	155653	157749
29840.t000028	*Ricinius communis*	29840	490975	491700
29848.t000064	*Ricinius communis*	29848	351983	352513
29908.t000067	*Ricinius communis*	29908	1100604	1102816
29908.t000120	*Ricinius communis*	29908	1444974	1446384
29974.t000011	*Ricinius communis*	29974	84520	85233
30066.t000002	*Ricinius communis*	30066	17735	18373
30076.t000130	*Ricinius communis*	30076	829213	830178
30106.t000001	*Ricinius communis*	30106	122825	124048
30131.t000316	*Ricinius communis*	30131	1971064	1971495
30136.t000017	*Ricinius communis*	30136	408702	409677
30190.t000107	*Ricinius communis*	30190	320390	321542
30226.t000055	*Ricinius communis*	30226	1809026	1809541
Aco000466	*Ananas comosus*	LG12	1655570	1656166
Aco000601	*Ananas comosus*	LG12	724824	725195
Aco000747	*Ananas comosus*	LG02	17023432	17023812
Aco001196	*Ananas comosus*	LG02	13459813	13461018
Aco001722	*Ananas comosus*	LG18	8459367	8459918
Aco003362	*Ananas comosus*	LG17	2354011	2354571
Aco004064	*Ananas comosus*	LG15	1639011	1642420
Aco004090	*Ananas comosus*	LG15	1476513	1477508
Aco004363	*Ananas comosus*	LG05	2644985	2645299
Aco005493	*Ananas comosus*	LG11	10658565	10658864
Aco005609	*Ananas comosus*	LG11	11687253	11687831
Aco006014	*Ananas comosus*	LG16	9708045	9708779
Aco008349	*Ananas comosus*	LG19	9699476	9700198
Aco008593	*Ananas comosus*	LG09	951439	951951
Aco008756	*Ananas comosus*	LG09	2150723	2151214
Aco009553	*Ananas comosus*	LG01	1360334	1363981
Aco009572	*Ananas comosus*	LG01	1247102	1250787
Aco010682	*Ananas comosus*	LG10	2701319	2702163
Aco015019	*Ananas comosus*	LG04	11823385	11823813
Aco017124	*Ananas comosus*	LG14	11581598	11582047
Aco018255	*Ananas comosus*	LG01	14349915	14350667
Aco019270	*Ananas comosus*	LG13	3740599	3740973
Aco023939	*Ananas comosus*	LG13	3206150	3206788
Aco026698	*Ananas comosus*	LG10	10378360	10379301

Table S2

Aco027359	*Ananas comosus*	LG05	1864958	1865644
AHYPO_001099	*Amaranthus hypochondriacus*	scaffold_5	406148	407078
AHYPO_004130	*Amaranthus hypochondriacus*	scaffold_26	337083	337712
AHYPO_004517	*Amaranthus hypochondriacus*	scaffold_28	1320978	1321586
AHYPO_005030	*Amaranthus hypochondriacus*	scaffold_33	378546	379097
AHYPO_005138	*Amaranthus hypochondriacus*	scaffold_34	318053	318478
AHYPO_005397	*Amaranthus hypochondriacus*	scaffold_36	507434	507966
AHYPO_005475	*Amaranthus hypochondriacus*	scaffold_37	10603	11373
AHYPO_005526	*Amaranthus hypochondriacus*	scaffold_37	740578	741960
AHYPO_007029	*Amaranthus hypochondriacus*	scaffold_54	531312	532150
AHYPO_008856	*Amaranthus hypochondriacus*	scaffold_80	40491	41632
AHYPO_008920	*Amaranthus hypochondriacus*	scaffold_80	697006	697689
AHYPO_010632	*Amaranthus hypochondriacus*	scaffold_108	9147	9647
AHYPO_010728	*Amaranthus hypochondriacus*	scaffold_110	156150	156518
AHYPO_013325	*Amaranthus hypochondriacus*	scaffold_176	215314	216000
AHYPO_017474	*Amaranthus hypochondriacus*	scaffold_388	193182	194324
AHYPO_017689	*Amaranthus hypochondriacus*	scaffold_404	199562	201494
AHYPO_018116	*Amaranthus hypochondriacus*	scaffold_446	18753	19367
AHYPO_018969	*Amaranthus hypochondriacus*	scaffold_525	106359	107657
AHYPO_019400	*Amaranthus hypochondriacus*	scaffold_580	118009	118701
AHYPO_020163	*Amaranthus hypochondriacus*	scaffold_704	44683	45369
AHYPO_021211	*Amaranthus hypochondriacus*	scaffold_931	26229	26738
AHYPO_021424	*Amaranthus hypochondriacus*	scaffold_993	29858	30538
AHYPO_021430	*Amaranthus hypochondriacus*	scaffold_993	77034	79055
AHYPO_022802	*Amaranthus hypochondriacus*	scaffold_1781	11830	12327
AL1G29370	*Arabidopsis lyrata*	scaffold_1	7174413	7175015
AL1G34420	*Arabidopsis lyrata*	scaffold_1	9195956	9197013
AL1G41830	*Arabidopsis lyrata*	scaffold_1	12456597	12457621
AL1G46870	*Arabidopsis lyrata*	scaffold_1	14939365	14940875
AL1G50790	*Arabidopsis lyrata*	scaffold_1	17684965	17686324
AL2G27310	*Arabidopsis lyrata*	scaffold_2	13095244	13096004
AL2G38660	*Arabidopsis lyrata*	scaffold_2	18129874	18131206
AL2G38780	*Arabidopsis lyrata*	scaffold_2	18174801	18175287
AL2G41050	*Arabidopsis lyrata*	scaffold_2	19034167	19038641
AL3G27690	*Arabidopsis lyrata*	scaffold_3	6390187	6391091
AL3G31640	*Arabidopsis lyrata*	scaffold_3	7876735	7877780
AL3G32010	*Arabidopsis lyrata*	scaffold_3	8019066	8019907
AL3G32030	*Arabidopsis lyrata*	scaffold_3	8026639	8027547
AL3G36350	*Arabidopsis lyrata*	scaffold_3	9840684	9841416
AL3G48200	*Arabidopsis lyrata*	scaffold_3	20721603	20722067
AL4G12470	*Arabidopsis lyrata*	scaffold_4	1323901	1324251
AL4G29490	*Arabidopsis lyrata*	scaffold_4	16016544	16017452
AL4G31260	*Arabidopsis lyrata*	scaffold_4	16718270	16720513
AL4G38990	*Arabidopsis lyrata*	scaffold_4	20006832	20007766
AL4G39150	*Arabidopsis lyrata*	scaffold_4	20061611	20062583
AL4G40510	*Arabidopsis lyrata*	scaffold_4	20594104	20594857
AL4G43320	*Arabidopsis lyrata*	scaffold_4	21624331	21625378
AL5G38080	*Arabidopsis lyrata*	scaffold_5	18035613	18036387
AL5G38270	*Arabidopsis lyrata*	scaffold_5	18115922	18116980
AL5G39650	*Arabidopsis lyrata*	scaffold_5	18673181	18674065
AL5G42290	*Arabidopsis lyrata*	scaffold_5	19664857	19665676
AL6G18950	*Arabidopsis lyrata*	scaffold_6	3343470	3344785
AL7G11920	*Arabidopsis lyrata*	scaffold_7	716690	717826
AL7G12970	*Arabidopsis lyrata*	scaffold_7	1144770	1145240
AL7G33860	*Arabidopsis lyrata*	scaffold_7	9831642	9832504
AL7G40590	*Arabidopsis lyrata*	scaffold_7	13290775	13291600
AL8G11560	*Arabidopsis lyrata*	scaffold_8	724304	725534
AL8G28890	*Arabidopsis lyrata*	scaffold_8	16091231	16092298

Table S2

AL8G42690	*Arabidopsis lyrata*	scaffold_8	21808737	21810168
Bol005142	*Brassica oleracea*	C04	39127651	39128328
Bol006908	*Brassica oleracea*	C04	342147	342572
Bol008035	*Brassica oleracea*	C02	41694703	41695668
Bol008412	*Brassica oleracea*	C03	44149264	44149965
Bol008485	*Brassica oleracea*	C07	28512004	28512696
Bol008806	*Brassica oleracea*	C03	1638556	1639080
Bol009641	*Brassica oleracea*	C05	16686132	16686824
Bol011139	*Brassica oleracea*	C05	28184470	28185147
Bol015936	*Brassica oleracea*	C03	29890388	29891476
Bol017211	*Brassica oleracea*	C07	26141099	26141779
Bol018598	*Brassica oleracea*	C07	47784777	47785121
Bol018960	*Brassica oleracea*	C02	20750497	20750811
Bol018964	*Brassica oleracea*	C02	20685672	20686553
Bol020569	*Brassica oleracea*	C03	11864857	11865282
Bol021449	*Brassica oleracea*	C06	30299005	30299523
Bol021556	*Brassica oleracea*	C06	31297762	31298079
Bol021563	*Brassica oleracea*	C06	31356709	31357749
Bol021672	*Brassica oleracea*	C04	40021677	40022219
Bol022878	*Brassica oleracea*	C03	23112762	23113400
Bol023244	*Brassica oleracea*	C01	28340238	28340828
Bol023823	*Brassica oleracea*	C06	27703295	27703678
Bol027428	*Brassica oleracea*	C04	21708508	21709734
Bol027483	*Brassica oleracea*	C06	736346	736660
Bol027489	*Brassica oleracea*	C06	764423	765289
Bol028835	*Brassica oleracea*	C01	304002	304871
Bol029300	*Brassica oleracea*	C08	19925801	19926094
Bol029826	*Brassica oleracea*	C03	47839399	47840001
Bol030074	*Brassica oleracea*	C04	2050948	2051490
Bol030076	*Brassica oleracea*	C04	2080607	2081164
Bol030935	*Brassica oleracea*	C01	31572529	31573371
Bol030958	*Brassica oleracea*	C01	31335705	31336358
Bol032726	*Brassica oleracea*	C03	47191222	47191566
Bol037818	*Brassica oleracea*	C04	36017455	36018483
Bol038290	*Brassica oleracea*	C05	6971415	6971708
Bol038790	*Brassica oleracea*	C09	21666963	21667646
Bol039807	*Brassica oleracea*	C03	8599256	8600557
Bol043792	*Brassica oleracea*	C09	37749496	37758167
Bol044298	*Brassica oleracea*	C04	22062301	22062570
Bol044299	*Brassica oleracea*	C04	22060798	22061250
Bol044309	*Brassica oleracea*	C04	21981182	21981892
Bol044405	*Brassica oleracea*	C08	30378433	30378882
Bol045470	*Brassica oleracea*	C08	30513115	30513804
Bol045527	*Brassica oleracea*	C08	31223700	31224233
Bol045596	*Brassica oleracea*	C08	32049827	32050300
Bol045819	*Brassica oleracea*	C08	34949314	34949730
Bradi1g06260	*Brachypodium distachyon*	Bd1	4197109	4198319
Bradi1g12580	*Brachypodium distachyon*	Bd1	9472882	9473810
Bradi1g17290	*Brachypodium distachyon*	Bd1	13834851	13836072
Bradi1g32060	*Brachypodium distachyon*	Bd1	27535170	27536378
Bradi1g35890	*Brachypodium distachyon*	Bd1	31639180	31639731
Bradi1g36630	*Brachypodium distachyon*	Bd1	32421151	32422605
Bradi1g38740	*Brachypodium distachyon*	Bd1	35221274	35222042
Bradi1g48842	*Brachypodium distachyon*	Bd1	47669774	47670145
Bradi1g56950	*Brachypodium distachyon*	Bd1	55838892	55839737
Bradi1g60860	*Brachypodium distachyon*	Bd1	60441437	60442192
Bradi1g63980	*Brachypodium distachyon*	Bd1	63398462	63399375
Bradi1g64050	*Brachypodium distachyon*	Bd1	63475028	63477787

Table S2

Bradi1g71870	*Brachypodium distachyon*	Bd1	69997991	69998989
Bradi2g06271	*Brachypodium distachyon*	Bd2	4714845	4715957
Bradi2g10700	*Brachypodium distachyon*	Bd2	8910180	8911483
Bradi2g15795	*Brachypodium distachyon*	Bd2	13950979	13953102
Bradi2g19600	*Brachypodium distachyon*	Bd2	17237946	17239509
Bradi2g21400	*Brachypodium distachyon*	Bd2	18761652	18762777
Bradi2g49740	*Brachypodium distachyon*	Bd2	49546077	49547278
Bradi2g52881	*Brachypodium distachyon*	Bd2	51916446	51917607
Bradi3g05410	*Brachypodium distachyon*	Bd3	3826120	3827605
Bradi3g09510	*Brachypodium distachyon*	Bd3	7634424	7636986
Bradi3g13033	*Brachypodium distachyon*	Bd3	11673785	11674615
Bradi3g34300	*Brachypodium distachyon*	Bd3	36473552	36476417
Bradi3g34440	*Brachypodium distachyon*	Bd3	36575683	36576351
Bradi3g35600	*Brachypodium distachyon*	Bd3	37639712	37641340
Bradi3g45220	*Brachypodium distachyon*	Bd3	46939354	46942028
Bradi3g58880	*Brachypodium distachyon*	Bd3	57969713	57970896
Bradi3g58890	*Brachypodium distachyon*	Bd3	57972887	57974035
Bradi4g00780	*Brachypodium distachyon*	Bd4	398093	398506
Bradi4g04497	*Brachypodium distachyon*	Bd4	3725814	3726574
Bradi4g29080	*Brachypodium distachyon*	Bd4	34401592	34403507
Bradi4g43770	*Brachypodium distachyon*	Bd4	47304079	47305500
Bradi5g03090	*Brachypodium distachyon*	Bd5	3420280	3423352
Bradi5g09730	*Brachypodium distachyon*	Bd5	13193982	13195768
Bradi5g23893	*Brachypodium distachyon*	Bd5	25852040	25853115
Bradi5g25360	*Brachypodium distachyon*	Bd5	26884424	26885277
Cagra.0007s0072	*Capsella grandiflora*	Scaffold7	252117	252746
Cagra.0096s0007	*Capsella grandiflora*	Scaffold96	27225	28475
Cagra.0301s0003	*Capsella grandiflora*	Scaffold301	6630	7223
Cagra.0578s0002	*Capsella grandiflora*	Scaffold578	2854	3736
Cagra.0578s0017	*Capsella grandiflora*	Scaffold578	56569	57790
Cagra.0686s0018	*Capsella grandiflora*	Scaffold686	59621	60166
Cagra.0751s0021	*Capsella grandiflora*	Scaffold751	87995	89022
Cagra.0824s0083	*Capsella grandiflora*	Scaffold824	283091	283739
Cagra.0917s0068	*Capsella grandiflora*	Scaffold917	299832	301160
Cagra.0993s0033	*Capsella grandiflora*	Scaffold993	110898	113030
Cagra.10347s0001	*Capsella grandiflora*	Scaffold10347	469	1425
Cagra.12804s0011	*Capsella grandiflora*	Scaffold12804	23717	24220
Cagra.1365s0059	*Capsella grandiflora*	Scaffold1365	201641	202168
Cagra.1562s0019	*Capsella grandiflora*	Scaffold1562	74015	74754
Cagra.1642s0067	*Capsella grandiflora*	Scaffold1642	236301	236996
Cagra.1642s0082	*Capsella grandiflora*	Scaffold1642	287433	288419
Cagra.1681s0005	*Capsella grandiflora*	Scaffold1681	15944	16753
Cagra.1725s0099	*Capsella grandiflora*	Scaffold1725	375526	376342
Cagra.1822s0034	*Capsella grandiflora*	Scaffold1822	141282	142464
Cagra.18423s0001	*Capsella grandiflora*	Scaffold18423	888	1427
Cagra.1850s0015	*Capsella grandiflora*	Scaffold1850	66987	67562
Cagra.1934s0003	*Capsella grandiflora*	Scaffold1934	15731	16066
Cagra.22718s0002	*Capsella grandiflora*	Scaffold22718	3543	8913
Cagra.2374s0064	*Capsella grandiflora*	Scaffold2374	217629	218504
Cagra.2420s0009	*Capsella grandiflora*	Scaffold2420	33053	33807
Cagra.2453s0017	*Capsella grandiflora*	Scaffold2453	51346	51873
Cagra.2460s0045	*Capsella grandiflora*	Scaffold2460	148294	149262
Cagra.2564s0020	*Capsella grandiflora*	Scaffold2564	66637	67251
Cagra.3364s0001	*Capsella grandiflora*	Scaffold3364	6048	6500
Cagra.3527s0041	*Capsella grandiflora*	Scaffold3527	169422	169895
Cagra.4290s0009	*Capsella grandiflora*	Scaffold4290	46171	46512
Cagra.7865s0021	*Capsella grandiflora*	Scaffold7865	76014	76673
Cagra.9757s0006	*Capsella grandiflora*	Scaffold9757	14250	14600

Table S2

Carubv10000171m.g	*Capsella rubella*	scaffold_6	14094249	14101213
Carubv10003884m.g	*Capsella rubella*	scaffold_6	2733377	2733904
Carubv10005634m.g	*Capsella rubella*	scaffold_7	7966010	7966898
Carubv10007072m.g	*Capsella rubella*	scaffold_7	577405	578283
Carubv10007212m.g	*Capsella rubella*	scaffold_7	912686	913027
Carubv10007700m.g	*Capsella rubella*	scaffold_7	10249553	10250131
Carubv10010726m.g	*Capsella rubella*	scaffold_1	5914929	5915654
Carubv10010842m.g	*Capsella rubella*	scaffold_1	7449818	7450531
Carubv10011477m.g	*Capsella rubella*	scaffold_1	9693152	9693898
Carubv10012223m.g	*Capsella rubella*	scaffold_1	12867270	12868226
Carubv10012318m.g	*Capsella rubella*	scaffold_1	11484967	11485632
Carubv10014701m.g	*Capsella rubella*	scaffold_3	8062389	8063338
Carubv10015299m.g	*Capsella rubella*	scaffold_3	6556098	6556751
Carubv10015457m.g	*Capsella rubella*	scaffold_3	5259334	5259963
Carubv10016148m.g	*Capsella rubella*	scaffold_3	6432944	6433750
Carubv10017877m.g	*Capsella rubella*	scaffold_5	11303808	11305708
Carubv10018159m.g	*Capsella rubella*	scaffold_5	11246744	11247770
Carubv10018493m.g	*Capsella rubella*	scaffold_5	11761471	11762016
Carubv10019258m.g	*Capsella rubella*	scaffold_5	12488959	12489432
Carubv10020938m.g	*Capsella rubella*	scaffold_2	13873238	13874288
Carubv10021179m.g	*Capsella rubella*	scaffold_2	13183327	13183917
Carubv10021365m.g	*Capsella rubella*	scaffold_2	13150139	13151089
Carubv10021516m.g	*Capsella rubella*	scaffold_2	9044485	9044931
Carubv10023326m.g	*Capsella rubella*	scaffold_4	9772254	9774812
Carubv10024274m.g	*Capsella rubella*	scaffold_4	12313309	12314660
Carubv10024521m.g	*Capsella rubella*	scaffold_4	13540243	13540776
Carubv10024784m.g	*Capsella rubella*	scaffold_4	12262033	12262737
Carubv10024877m.g	*Capsella rubella*	scaffold_4	815388	815738
Carubv10025399m.g	*Capsella rubella*	scaffold_4	12711124	12711624
Carubv10025526m.g	*Capsella rubella*	scaffold_4	9190247	9190840
Carubv10026700m.g	*Capsella rubella*	scaffold_8	12412832	12414102
Carubv10026943m.g	*Capsella rubella*	scaffold_8	425270	426408
Carubv10026984m.g	*Capsella rubella*	scaffold_8	7960166	7961221
Carubv10027887m.g	*Capsella rubella*	scaffold_8	1446960	1447697
Ciclev10001250m.g	*Citrus clementina*	scaffold_5	36490752	36492636
Ciclev10001938m.g	*Citrus clementina*	scaffold_5	36166730	36168991
Ciclev10002106m.g	*Citrus clementina*	scaffold_5	38469258	38470252
Ciclev10002803m.g	*Citrus clementina*	scaffold_5	21399871	21400827
Ciclev10009260m.g	*Citrus clementina*	scaffold_1	17418249	17419200
Ciclev10009660m.g	*Citrus clementina*	scaffold_1	891333	892160
Ciclev10012154m.g	*Citrus clementina*	scaffold_6	3003862	3006390
Ciclev10012618m.g	*Citrus clementina*	scaffold_6	21681866	21683202
Ciclev10012635m.g	*Citrus clementina*	scaffold_6	18451376	18452564
Ciclev10013474m.g	*Citrus clementina*	scaffold_6	18907844	18909431
Ciclev10013749m.g	*Citrus clementina*	scaffold_6	19555041	19555523
Ciclev10016465m.g	*Citrus clementina*	scaffold_2	33532219	33534581
Ciclev10016595m.g	*Citrus clementina*	scaffold_2	29886356	29887239
Ciclev10017188m.g	*Citrus clementina*	scaffold_2	35199443	35200114
Ciclev10022688m.g	*Citrus clementina*	scaffold_3	35784884	35785592
Ciclev10023693m.g	*Citrus clementina*	scaffold_3	10065274	10065846
Ciclev10023791m.g	*Citrus clementina*	scaffold_3	10062212	10062880
Ciclev10025465m.g	*Citrus clementina*	scaffold_7	20690094	20692017
Ciclev10026434m.g	*Citrus clementina*	scaffold_7	16614911	16616240
Ciclev10026439m.g	*Citrus clementina*	scaffold_7	12890748	12891580
Ciclev10026545m.g	*Citrus clementina*	scaffold_7	4127518	4128342
Ciclev10026619m.g	*Citrus clementina*	scaffold_7	5805230	5807265
Ciclev10033296m.g	*Citrus clementina*	scaffold_4	15559529	15560119
Ciclev10033421m.g	*Citrus clementina*	scaffold_4	15555148	15555729

Table S2

Ciclev10033922m.g	*Citrus clementina*	scaffold_4	25176534	25176812
Cucsa.029510	*Cucumis sativus*	scaffold00397	125234	126451
Cucsa.046270	*Cucumis sativus*	scaffold00542	1825721	1826395
Cucsa.048710	*Cucumis sativus*	scaffold00542	3351717	3352523
Cucsa.058430	*Cucumis sativus*	scaffold00614	588527	588996
Cucsa.068360	*Cucumis sativus*	scaffold00707	144292	145870
Cucsa.077000	*Cucumis sativus*	scaffold00791	96254	96787
Cucsa.103330	*Cucumis sativus*	scaffold00927	2138904	2139410
Cucsa.104470	*Cucumis sativus*	scaffold00927	2971587	2972971
Cucsa.105580	*Cucumis sativus*	scaffold00929	200584	200948
Cucsa.123100	*Cucumis sativus*	scaffold01000	402209	402960
Cucsa.126760	*Cucumis sativus*	scaffold01017	610078	610830
Cucsa.133030	*Cucumis sativus*	scaffold01037	825162	825684
Cucsa.160170	*Cucumis sativus*	scaffold01144	433448	434839
Cucsa.170100	*Cucumis sativus*	scaffold01174	390450	390950
Cucsa.180120	*Cucumis sativus*	scaffold01233	459174	459717
Cucsa.199120	*Cucumis sativus*	scaffold01357	1923147	1923680
Cucsa.242220	*Cucumis sativus*	scaffold02047	1997651	1997965
Cucsa.242710	*Cucumis sativus*	scaffold02047	2389018	2389536
Cucsa.249990	*Cucumis sativus*	scaffold02219	126609	127093
Cucsa.271370	*Cucumis sativus*	scaffold02500	267604	268131
Cucsa.283500	*Cucumis sativus*	scaffold02653	591228	591659
Cucsa.291310	*Cucumis sativus*	scaffold02764	69714	70112
Cucsa.307850	*Cucumis sativus*	scaffold02978	126553	127441
Cucsa.311600	*Cucumis sativus*	scaffold02995	1146449	1147453
Cucsa.312720	*Cucumis sativus*	scaffold02995	2049367	2050356
Cucsa.313130	*Cucumis sativus*	scaffold02995	2372156	2373112
Cucsa.321830	*Cucumis sativus*	scaffold03080	1646873	1648697
Cucsa.337490	*Cucumis sativus*	scaffold03356	563520	564242
Cucsa.337760	*Cucumis sativus*	scaffold03356	765288	766585
Cucsa.394710	*Cucumis sativus*	scaffold04100	520796	521509
DCAR_001091	*Daucus carota*	DCARv2_Chr1	10502163	10502804
DCAR_001286	*Daucus carota*	DCARv2_Chr1	4075578	4076270
DCAR_001357	*Daucus carota*	DCARv2_Chr1	13845888	13846493
DCAR_001486	*Daucus carota*	DCARv2_Chr1	15982473	15983447
DCAR_004068	*Daucus carota*	DCARv2_Chr1	44423133	44423852
DCAR_004339	*Daucus carota*	DCARv2_Chr1	46974176	46974886
DCAR_005628	*Daucus carota*	DCARv2_Chr2	16020411	16021496
DCAR_008272	*Daucus carota*	DCARv2_Chr2	40816946	40818058
DCAR_008723	*Daucus carota*	DCARv2_Chr3	614567	615999
DCAR_010807	*Daucus carota*	DCARv2_Chr3	28895780	28896253
DCAR_012621	*Daucus carota*	DCARv2_Chr3	49453195	49456150
DCAR_012887	*Daucus carota*	DCARv2_Chr4	34505164	34505949
DCAR_013031	*Daucus carota*	DCARv2_Chr4	33394276	33394947
DCAR_013320	*Daucus carota*	DCARv2_Chr4	31060697	31061446
DCAR_013523	*Daucus carota*	DCARv2_Chr4	29526296	29526946
DCAR_013546	*Daucus carota*	DCARv2_Chr4	29355399	29355929
DCAR_013643	*Daucus carota*	DCARv2_Chr4	28607497	28607916
DCAR_013798	*Daucus carota*	DCARv2_Chr4	27297705	27298025
DCAR_014231	*Daucus carota*	DCARv2_Chr4	23714186	23716156
DCAR_014231	*Daucus carota*	DCARv2_Chr4	23714186	23716156
DCAR_014345	*Daucus carota*	DCARv2_Chr4	22620815	22621417
DCAR_014416	*Daucus carota*	DCARv2_Chr4	22061047	22062202
DCAR_014417	*Daucus carota*	DCARv2_Chr4	22060231	22060950
DCAR_015713	*Daucus carota*	DCARv2_Chr4	8759751	8760122
DCAR_016273	*Daucus carota*	DCARv2_Chr5	1844483	1844884
DCAR_016274	*Daucus carota*	DCARv2_Chr5	1850971	1851426
DCAR_016902	*Daucus carota*	DCARv2_Chr5	8993239	8993772

Table S2

DCAR_017566	*Daucus carota*	DCARv2_Chr5	20975820	20976128
DCAR_019562	*Daucus carota*	DCARv2_Chr5	40096873	40097517
DCAR_019636	*Daucus carota*	DCARv2_Chr5	40681965	40682252
DCAR_020574	*Daucus carota*	DCARv2_Chr6	30198073	30198564
DCAR_020610	*Daucus carota*	DCARv2_Chr6	29887840	29888400
DCAR_021057	*Daucus carota*	DCARv2_Chr6	26183966	26184271
DCAR_021260	*Daucus carota*	DCARv2_Chr6	24622348	24622944
DCAR_021355	*Daucus carota*	DCARv2_Chr6	23771056	23771670
DCAR_023208	*Daucus carota*	DCARv2_Chr6	676011	676694
DCAR_024218	*Daucus carota*	DCARv2_Chr7	10825863	10826573
DCAR_025326	*Daucus carota*	DCARv2_Chr7	26068523	26069140
DCAR_026737	*Daucus carota*	DCARv2_Chr8	29206647	29207198
DCAR_028062	*Daucus carota*	DCARv2_Chr8	15576599	15577273
DCAR_029592	*Daucus carota*	DCARv2_Chr9	9499241	9499579
Eucgr.A02182	*Eucalyptus grandis*	Chr01	37113647	37114778
Eucgr.A02242	*Eucalyptus grandis*	Chr01	37821284	37822147
Eucgr.A02450	*Eucalyptus grandis*	Chr01	39836460	39837326
Eucgr.A02544	*Eucalyptus grandis*	Chr01	40671118	40671423
Eucgr.B00035	*Eucalyptus grandis*	Chr06	35375963	35378557
Eucgr.C02621	*Eucalyptus grandis*	Chr03	51875030	51875674
Eucgr.C02629	*Eucalyptus grandis*	Chr03	51927510	51928462
Eucgr.D00026	*Eucalyptus grandis*	Chr04	216578	217627
Eucgr.D00026	*Eucalyptus grandis*	Chr04	215594	217627
Eucgr.D02200	*Eucalyptus grandis*	Chr04	35568137	35570178
Eucgr.F00302	*Eucalyptus grandis*	Chr06	5689343	5690714
Eucgr.F00371	*Eucalyptus grandis*	Chr06	5040555	5041763
Eucgr.F01148	*Eucalyptus grandis*	Chr06	15726039	15727038
Eucgr.F01407	*Eucalyptus grandis*	Chr06	19292825	19293671
Eucgr.F01408	*Eucalyptus grandis*	Chr06	19298931	19299260
Eucgr.F02723	*Eucalyptus grandis*	Chr06	39354381	39355067
Eucgr.F03907	*Eucalyptus grandis*	Chr06	49847862	49848569
Eucgr.G02312	*Eucalyptus grandis*	Chr07	43757342	43758655
Eucgr.G03218	*Eucalyptus grandis*	Chr07	52999148	52999886
Eucgr.H02317	*Eucalyptus grandis*	Chr08	29626932	29627764
Eucgr.H02318	*Eucalyptus grandis*	Chr08	29632300	29632908
Eucgr.H02319	*Eucalyptus grandis*	Chr08	29636679	29638276
Eucgr.H04261	*Eucalyptus grandis*	Chr08	57364319	57364817
Eucgr.H04807	*Eucalyptus grandis*	Chr08	67074212	67075837
Eucgr.I00016	*Eucalyptus grandis*	Chr09	223638	224213
Eucgr.I00226	*Eucalyptus grandis*	Chr09	4661485	4662947
Eucgr.K01342	*Eucalyptus grandis*	Chr11	16771278	16772474
Eucgr.K01686	*Eucalyptus grandis*	Chr11	19466093	19467503
evm.TU.contig_3510	*Carica papaya*	contig_35103	2783	3391
evm.TU.contig_39771	*Carica papaya*	contig_39771	19	701
*evm.TU.supercontig_*	*Carica papaya*	supercontig_1	572889	573437
*evm.TU.supercontig_*	*Carica papaya*	supercontig_129	754778	755380
*evm.TU.supercontig_*	*Carica papaya*	supercontig_148	562698	563442
*evm.TU.supercontig_*	*Carica papaya*	supercontig_162	36793	37182
*evm.TU.supercontig_*	*Carica papaya*	supercontig_17	107298	107645
*evm.TU.supercontig_*	*Carica papaya*	supercontig_189	16803	17779
*evm.TU.supercontig_*	*Carica papaya*	supercontig_2	2767619	2768176
*evm.TU.supercontig_*	*Carica papaya*	supercontig_2579	2413	2748
*evm.TU.supercontig_*	*Carica papaya*	supercontig_27	351979	352812
*evm.TU.supercontig_*	*Carica papaya*	supercontig_3	3603897	3604613
*evm.TU.supercontig_*	*Carica papaya*	supercontig_30	1690330	1691040
*evm.TU.supercontig_*	*Carica papaya*	supercontig_30	1707944	1708495
*evm.TU.supercontig_*	*Carica papaya*	supercontig_33	1860672	1861343
*evm.TU.supercontig_*	*Carica papaya*	supercontig_36	960055	961404

Table S2

*evm.TU.supercontig_*	*Carica papaya*	supercontig_36	1236238	1237314
*evm.TU.supercontig_*	*Carica papaya*	supercontig_42	1041214	1041807
*evm.TU.supercontig_*	*Carica papaya*	supercontig_49	331828	332241
*evm.TU.supercontig_*	*Carica papaya*	supercontig_49	340756	341478
*evm.TU.supercontig_*	*Carica papaya*	supercontig_5	1416914	1417462
*evm.TU.supercontig_*	*Carica papaya*	supercontig_568	9364	10848
*evm.TU.supercontig_*	*Carica papaya*	supercontig_71	1059159	1060157
*evm.TU.supercontig_*	*Carica papaya*	supercontig_8	2046215	2046994
*evm.TU.supercontig_*	*Carica papaya*	supercontig_80	570809	571582
evm_27.TU.AmTr_v1.	*Amborella trichopoda*	AmTr_v1.0_scaffold00002	4628951	4629823
evm_27.TU.AmTr_v1.	*Amborella trichopoda*	AmTr_v1.0_scaffold00004	2267431	2268150
evm_27.TU.AmTr_v1.	*Amborella trichopoda*	AmTr_v1.0_scaffold00010	2891832	2892812
evm_27.TU.AmTr_v1.	*Amborella trichopoda*	AmTr_v1.0_scaffold00014	5280287	5280670
evm_27.TU.AmTr_v1.	*Amborella trichopoda*	AmTr_v1.0_scaffold00019	818411	818989
evm_27.TU.AmTr_v1.	*Amborella trichopoda*	AmTr_v1.0_scaffold00024	5753895	5754503
evm_27.TU.AmTr_v1.	*Amborella trichopoda*	AmTr_v1.0_scaffold00030	2074031	2074645
evm_27.TU.AmTr_v1.	*Amborella trichopoda*	AmTr_v1.0_scaffold00058	4510012	4510278
evm_27.TU.AmTr_v1.	*Amborella trichopoda*	AmTr_v1.0_scaffold00065	1737311	1737790
evm_27.TU.AmTr_v1.	*Amborella trichopoda*	AmTr_v1.0_scaffold00073	2547671	2548456
evm_27.TU.AmTr_v1.	*Amborella trichopoda*	AmTr_v1.0_scaffold00079	1632707	1633330
evm_27.TU.AmTr_v1.	*Amborella trichopoda*	AmTr_v1.0_scaffold00095	2948215	2948856
evm_27.TU.AmTr_v1.	*Amborella trichopoda*	AmTr_v1.0_scaffold00095	700786	701160
evm_27.TU.AmTr_v1.	*Amborella trichopoda*	AmTr_v1.0_scaffold00099	1326801	1327535
evm_27.TU.AmTr_v1.	*Amborella trichopoda*	AmTr_v1.0_scaffold00183	260284	260751
gene00230-v1.0-hybr	*Fragaria vesca*	LG6	3509186	3509932
gene02352-v1.0-hybr	*Fragaria vesca*	LG3	8950952	8951329
gene02378-v1.0-hybr	*Fragaria vesca*	LG3	5408370	5409131
gene03724-v1.0-hybr	*Fragaria vesca*	LG4	25161674	25162408
gene05795-v1.0-hybr	*Fragaria vesca*	LG1	15137546	15138079
gene11973-v1.0-hybr	*Fragaria vesca*	LG5	23784866	23785858
gene12350-v1.0-hybr	*Fragaria vesca*	unanchored	11462621	11463181
gene14221-v1.0-hybr	*Fragaria vesca*	LG7	6613976	6614374
gene15994-v1.0-hybr	*Fragaria vesca*	LG6	22989178	22989765
gene16338-v1.0-hybr	*Fragaria vesca*	LG1	18751864	18753246
gene16601-v1.0-hybr	*Fragaria vesca*	LG6	356206	356601
gene17096-v1.0-hybr	*Fragaria vesca*	LG4	6739897	6745549
gene17872-v1.0-hybr	*Fragaria vesca*	LG1	12887614	12888429
gene18133-v1.0-hybr	*Fragaria vesca*	LG6	17840134	17840580
gene19985-v1.0-hybr	*Fragaria vesca*	LG3	7372485	7373201
gene21583-v1.0-hybr	*Fragaria vesca*	LG6	10183937	10184386
gene22130-v1.0-hybr	*Fragaria vesca*	LG5	27235620	27236900
gene22640-v1.0-hybr	*Fragaria vesca*	LG6	18793588	18794142
gene22762-v1.0-hybr	*Fragaria vesca*	LG4	20408371	20409318
gene24219-v1.0-hybr	*Fragaria vesca*	LG6	30980995	30981720
gene25153-v1.0-hybr	*Fragaria vesca*	LG5	6574758	6575447
gene26510-v1.0-hybr	*Fragaria vesca*	LG1	3368020	3371633
gene32666-v1.0-hybr	*Fragaria vesca*	LG2	5336158	5338705
Gorai.001G039000	*Gossypium raimondii*	Chr01	3638826	3639500
Gorai.001G093400	*Gossypium raimondii*	Chr01	10392223	10393758
Gorai.002G038600	*Gossypium raimondii*	Chr02	3085749	3086317
Gorai.002G069400	*Gossypium raimondii*	Chr02	8048653	8050042
Gorai.002G069400	*Gossypium raimondii*	Chr02	8048444	8050064
Gorai.002G069400	*Gossypium raimondii*	Chr02	8048444	8050064
Gorai.002G139900	*Gossypium raimondii*	Chr02	24266863	24268840
Gorai.002G154000	*Gossypium raimondii*	Chr02	31609529	31609955
Gorai.002G224500	*Gossypium raimondii*	Chr02	58000003	58000651
Gorai.002G226300	*Gossypium raimondii*	Chr02	58252023	58253235
Gorai.002G233400	*Gossypium raimondii*	Chr02	59303577	59304105

Table S2

Gorai.003G089500	*Gossypium raimondii*	Chr03	25751332	25752625
Gorai.003G181800	*Gossypium raimondii*	Chr03	45341646	45343844
Gorai.003G181800	*Gossypium raimondii*	Chr03	45341646	45343914
Gorai.003G181800	*Gossypium raimondii*	Chr03	45341646	45343914
Gorai.004G112000	*Gossypium raimondii*	Chr04	25584480	25585145
Gorai.004G185400	*Gossypium raimondii*	Chr04	49963794	49964957
Gorai.006G068000	*Gossypium raimondii*	Chr06	27358431	27359021
Gorai.006G142700	*Gossypium raimondii*	Chr06	40062726	40063143
Gorai.006G216500	*Gossypium raimondii*	Chr06	46953841	46955151
Gorai.007G056800	*Gossypium raimondii*	Chr07	4000985	4001943
Gorai.007G173300	*Gossypium raimondii*	Chr07	15926361	15927074
Gorai.007G174400	*Gossypium raimondii*	Chr07	16114297	16114767
Gorai.007G225500	*Gossypium raimondii*	Chr07	27046228	27047158
Gorai.007G240000	*Gossypium raimondii*	Chr07	32987122	32988679
Gorai.007G351100	*Gossypium raimondii*	Chr07	58139987	58140767
Gorai.007G351200	*Gossypium raimondii*	Chr07	58141334	58142323
Gorai.007G352700	*Gossypium raimondii*	Chr07	58326332	58327385
Gorai.008G000200	*Gossypium raimondii*	Chr08	40910	42234
Gorai.008G049400	*Gossypium raimondii*	Chr08	6919683	6920321
Gorai.008G049600	*Gossypium raimondii*	Chr08	6985375	6986635
Gorai.008G049600	*Gossypium raimondii*	Chr08	6985342	6986635
Gorai.008G084600	*Gossypium raimondii*	Chr08	18235440	18236008
Gorai.008G104800	*Gossypium raimondii*	Chr08	32632567	32634655
Gorai.009G064400	*Gossypium raimondii*	Chr09	4585317	4586353
Gorai.009G154700	*Gossypium raimondii*	Chr09	11863296	11863731
Gorai.009G168800	*Gossypium raimondii*	Chr09	12994913	12995930
Gorai.009G238300	*Gossypium raimondii*	Chr09	18923832	18925025
Gorai.009G281800	*Gossypium raimondii*	Chr09	23846494	23847294
Gorai.009G347500	*Gossypium raimondii*	Chr09	42996266	42997147
Gorai.009G384400	*Gossypium raimondii*	Chr09	52148752	52150015
Gorai.010G038300	*Gossypium raimondii*	Chr10	3571492	3574060
Gorai.010G042200	*Gossypium raimondii*	Chr10	3936551	3938464
Gorai.010G042200	*Gossypium raimondii*	Chr10	3936021	3938688
Gorai.010G119600	*Gossypium raimondii*	Chr10	24000804	24001806
Gorai.010G154500	*Gossypium raimondii*	Chr10	42421834	42423240
Gorai.011G044800	*Gossypium raimondii*	Chr11	3360599	3361238
Gorai.011G113000	*Gossypium raimondii*	Chr11	14227178	14229131
Gorai.011G116000	*Gossypium raimondii*	Chr11	15178174	15179308
Gorai.011G121400	*Gossypium raimondii*	Chr11	16780669	16783565
Gorai.011G217100	*Gossypium raimondii*	Chr11	52270072	52270818
Gorai.012G018900	*Gossypium raimondii*	Chr12	2257419	2258087
Gorai.013G186400	*Gossypium raimondii*	Chr13	48615105	48615437
GSMUA_Achr10G01	*Musa acuminata*	chr10	3649423	3649848
GSMUA_Achr10G01	*Musa acuminata*	chr10	3691936	3692529
GSMUA_Achr10G09	*Musa acuminata*	chr10	19283971	19284588
GSMUA_Achr10G14	*Musa acuminata*	chr10	22483722	22484438
GSMUA_Achr10G14	*Musa acuminata*	chr10	22674791	22675096
GSMUA_Achr11G10	*Musa acuminata*	chr11	7932669	7933034
GSMUA_Achr11G13	*Musa acuminata*	chr11	13357098	13357937
GSMUA_Achr1G209	*Musa acuminata*	chr1	15811157	15812051
GSMUA_Achr2G011	*Musa acuminata*	chr2	6335388	6336023
GSMUA_Achr2G025	*Musa acuminata*	chr2	8367836	8368703
GSMUA_Achr2G172	*Musa acuminata*	chr2	17835083	17835678
GSMUA_Achr2G221	*Musa acuminata*	chr2	21348507	21354969
GSMUA_Achr3G094	*Musa acuminata*	chr3	6788170	6788769
GSMUA_Achr3G198	*Musa acuminata*	chr3	21134104	21134724
GSMUA_Achr3G213	*Musa acuminata*	chr3	22323258	22324642
GSMUA_Achr4G025	*Musa acuminata*	chr4	2211540	2212585

Table S2

GSMUA_Achr4G104	*Musa acuminata*	chr4	7571839	7572474
GSMUA_Achr4G190	*Musa acuminata*	chr4	20125588	20133442
GSMUA_Achr4G273	*Musa acuminata*	chr4	26183378	26187950
GSMUA_Achr5G034	*Musa acuminata*	chr5	2377062	2378006
GSMUA_Achr5G077	*Musa acuminata*	chr5	5588907	5589563
GSMUA_Achr5G276	*Musa acuminata*	chr5	28103236	28109273
GSMUA_Achr6G020	*Musa acuminata*	chr6	1318572	1319378
GSMUA_Achr6G041	*Musa acuminata*	chr6	2814957	2816126
GSMUA_Achr6G130	*Musa acuminata*	chr6	8733188	8733889
GSMUA_Achr6G144	*Musa acuminata*	chr6	9513192	9513485
GSMUA_Achr6G320	*Musa acuminata*	chr6	31615018	31615721
GSMUA_Achr7G025	*Musa acuminata*	chr7	1986357	1986746
GSMUA_Achr7G032	*Musa acuminata*	chr7	2562804	2563457
GSMUA_Achr7G064	*Musa acuminata*	chr7	4722238	4722603
GSMUA_Achr7G078	*Musa acuminata*	chr7	5797001	5797471
GSMUA_Achr7G137	*Musa acuminata*	chr7	11046754	11047500
GSMUA_Achr7G191	*Musa acuminata*	chr7	21975468	21978593
GSMUA_Achr8G057	*Musa acuminata*	chr8	3763077	3767225
GSMUA_Achr8G181	*Musa acuminata*	chr8	21243917	21244684
GSMUA_Achr8G186	*Musa acuminata*	chr8	23154198	23155208
GSMUA_Achr8G215	*Musa acuminata*	chr8	26428085	26428877
GSMUA_Achr9G058	*Musa acuminata*	chr9	3694390	3695404
GSMUA_Achr9G172	*Musa acuminata*	chr9	11824035	11824373
GSMUA_Achr9G308	*Musa acuminata*	chr9	33958017	33959149
GSMUA_AchrUn_ra	*Musa acuminata*	chrUn_random	79129485	79130111
GSMUA_AchrUn_ra	*Musa acuminata*	chrUn_random	105894805	105895679
GSMUA_AchrUn_ra	*Musa acuminata*	chrUn_random	113307788	113308525
GSMUA_AchrUn_ra	*Musa acuminata*	chrUn_random	121966782	121967264
Lus10004172.g	*Linum usitatissimum*	scaffold2404	125224	126024
Lus10005523.g	*Linum usitatissimum*	scaffold290	75463	75990
Lus10005543.g	*Linum usitatissimum*	scaffold2324	16526	17284
Lus10006569.g	*Linum usitatissimum*	scaffold1202	178020	178550
Lus10006941.g	*Linum usitatissimum*	scaffold253	138913	140124
Lus10008659.g	*Linum usitatissimum*	scaffold1635	44551	44928
Lus10009033.g	*Linum usitatissimum*	scaffold883	179322	179987
Lus10010346.g	*Linum usitatissimum*	scaffold732	319294	320067
Lus10010347.g	*Linum usitatissimum*	scaffold732	323059	323421
Lus10011555.g	*Linum usitatissimum*	scaffold260	296672	297124
Lus10012286.g	*Linum usitatissimum*	scaffold273	405252	405470
Lus10012292.g	*Linum usitatissimum*	scaffold273	446740	448185
Lus10015315.g	*Linum usitatissimum*	scaffold924	459794	460537
Lus10015982.g	*Linum usitatissimum*	scaffold172	68770	69495
Lus10016205.g	*Linum usitatissimum*	scaffold947	199969	200721
Lus10016814.g	*Linum usitatissimum*	scaffold903	612690	613211
Lus10018519.g	*Linum usitatissimum*	scaffold1308	146596	147237
Lus10019270.g	*Linum usitatissimum*	scaffold377	276342	276836
Lus10020092.g	*Linum usitatissimum*	scaffold454	6891	7463
Lus10021054.g	*Linum usitatissimum*	scaffold155	306070	306840
Lus10022005.g	*Linum usitatissimum*	scaffold87	275850	276257
Lus10023308.g	*Linum usitatissimum*	scaffold98	460382	460720
Lus10024139.g	*Linum usitatissimum*	scaffold353	726228	726743
Lus10024738.g	*Linum usitatissimum*	scaffold320	169870	170934
Lus10026165.g	*Linum usitatissimum*	scaffold319	983787	984161
Lus10026247.g	*Linum usitatissimum*	scaffold898	309235	309855
Lus10027279.g	*Linum usitatissimum*	scaffold472	611141	611707
Lus10029339.g	*Linum usitatissimum*	scaffold360	743700	745891
Lus10029768.g	*Linum usitatissimum*	scaffold418	1126810	1127376
Lus10036438.g	*Linum usitatissimum*	scaffold57	643691	644197

Table S2

Lus10036479.g	*Linum usitatissimum*	scaffold57	809165	809971
Lus10036480.g	*Linum usitatissimum*	scaffold57	819667	820776
Lus10038502.g	*Linum usitatissimum*	scaffold28	1666263	1666622
Lus10038985.g	*Linum usitatissimum*	scaffold34	1133910	1134476
Lus10039493.g	*Linum usitatissimum*	scaffold15	33858	34346
Lus10039494.g	*Linum usitatissimum*	scaffold15	39990	40478
Lus10039654.g	*Linum usitatissimum*	scaffold15	1020951	1021700
Lus10039737.g	*Linum usitatissimum*	scaffold15	1418084	1418722
Lus10041112.g	*Linum usitatissimum*	scaffold280	1015614	1016120
Lus10041929.g	*Linum usitatissimum*	scaffold123	16113	16853
Lus10042423.g	*Linum usitatissimum*	scaffold123	2099300	2099923
Lus10042794.g	*Linum usitatissimum*	scaffold67	2113313	2113732
Manes.01G010900	*Manihot esculenta*	Chromosome01	1957364	1958047
Manes.01G233900	*Manihot esculenta*	Chromosome01	31617045	31618639
Manes.02G149700	*Manihot esculenta*	Chromosome02	11096736	11097803
Manes.02G153400	*Manihot esculenta*	Chromosome02	11507943	11508986
Manes.02G176600	*Manihot esculenta*	Chromosome02	14172705	14173841
Manes.03G074500	*Manihot esculenta*	Chromosome03	10193479	10194511
Manes.03G109400	*Manihot esculenta*	Chromosome03	20050569	20051977
Manes.04G110500	*Manihot esculenta*	Chromosome04	24466608	24467189
Manes.04G157400	*Manihot esculenta*	Chromosome04	28002483	28003304
Manes.05G009900	*Manihot esculenta*	Chromosome05	659403	661845
Manes.05G009900	*Manihot esculenta*	Chromosome05	659393	661788
Manes.05G009900	*Manihot esculenta*	Chromosome05	659403	661788
Manes.06G067000	*Manihot esculenta*	Chromosome06	18068109	18068705
Manes.08G037400	*Manihot esculenta*	Chromosome08	3433427	3433756
Manes.08G065900	*Manihot esculenta*	Chromosome08	8853040	8854127
Manes.08G137000	*Manihot esculenta*	Chromosome08	30312303	30312668
Manes.08G137100	*Manihot esculenta*	Chromosome08	30317556	30317942
Manes.09G001100	*Manihot esculenta*	Chromosome09	136322	137303
Manes.09G004900	*Manihot esculenta*	Chromosome09	1065966	1066768
Manes.09G035200	*Manihot esculenta*	Chromosome09	4923819	4924805
Manes.09G150100	*Manihot esculenta*	Chromosome09	26706449	26706856
Manes.10G152200	*Manihot esculenta*	Chromosome10	26201392	26201859
Manes.11G007000	*Manihot esculenta*	Chromosome11	745790	746631
Manes.11G059000	*Manihot esculenta*	Chromosome11	5775444	5776584
Manes.12G081300	*Manihot esculenta*	Chromosome12	9548387	9549756
Manes.12G116300	*Manihot esculenta*	Chromosome12	26816251	26816820
Manes.12G136100	*Manihot esculenta*	Chromosome12	29052990	29055396
Manes.12G152600	*Manihot esculenta*	Chromosome12	31040220	31040921
Manes.13G093500	*Manihot esculenta*	Chromosome13	19275821	19278110
Manes.13G109800	*Manihot esculenta*	Chromosome13	23690200	23691060
Manes.14G103400	*Manihot esculenta*	Chromosome14	8407513	8408157
Manes.15G087700	*Manihot esculenta*	Chromosome15	6411567	6413162
Manes.15G122000	*Manihot esculenta*	Chromosome15	9235521	9236417
Manes.16G028900	*Manihot esculenta*	Chromosome16	3006644	3008231
Manes.16G083700	*Manihot esculenta*	Chromosome16	24060638	24061141
Manes.16G132000	*Manihot esculenta*	Chromosome16	28193885	28194421
Manes.17G046600	*Manihot esculenta*	Chromosome17	18361605	18362761
Manes.17G070600	*Manihot esculenta*	Chromosome17	20992545	20993093
Manes.18G070800	*Manihot esculenta*	Chromosome18	6071939	6072511
Manes.S050000	*Manihot esculenta*	Scaffold00994	60398	61487
Manes.S072900	*Manihot esculenta*	Scaffold01201	30511	31101
Manes.S089300	*Manihot esculenta*	Scaffold01392	20081	20829
MDP0000122563	*Malus domestica*	MDC005944.589	12959	13735
MDP0000127852	*Malus domestica*	MDC013556.545	12592	13269
MDP0000130738	*Malus domestica*	MDC018913.57	3011	3796
MDP0000136351	*Malus domestica*	MDC007650.539	967	1626

Table S2

MDP0000148283	*Malus domestica*	MDC007489.200	12561	13794
MDP0000148903	*Malus domestica*	MDC005944.403	2509	3303
MDP0000152343	*Malus domestica*	MDC011891.338	2555	2959
MDP0000154438	*Malus domestica*	MDC039631.9	2009	2989
MDP0000159948	*Malus domestica*	MDC000216.352	17056	17844
MDP0000172223	*Malus domestica*	MDC002920.175	1364	1942
MDP0000176426	*Malus domestica*	MDC007216.431	6444	7603
MDP0000179253	*Malus domestica*	MDC005932.140	1556	2059
MDP0000179796	*Malus domestica*	MDC006484.324	1933	2340
MDP0000182585	*Malus domestica*	MDC007649.204	6202	6519
MDP0000182830	*Malus domestica*	MDC007963.100	929	2185
MDP0000183958	*Malus domestica*	MDC008307.239	492	1270
MDP0000193206	*Malus domestica*	MDC010400.331	158	904
MDP0000210996	*Malus domestica*	MDC016275.391	8283	9516
MDP0000216667	*Malus domestica*	MDC020990.323	2185	3534
MDP0000219970	*Malus domestica*	MDC023041.57	9301	10904
MDP0000227657	*Malus domestica*	MDC030946.7	5008	5679
MDP0000247951	*Malus domestica*	MDC000140.134	64996	70497
MDP0000248043	*Malus domestica*	MDC002948.92	18152	18697
MDP0000261758	*Malus domestica*	MDC018989.79	36407	39347
MDP0000264361	*Malus domestica*	MDC002488.213	4810	5445
MDP0000274905	*Malus domestica*	MDC003165.207	2451	6779
MDP0000278259	*Malus domestica*	MDC008307.240	6056	14342
MDP0000284090	*Malus domestica*	MDC007628.679	2533	16137
MDP0000295494	*Malus domestica*	MDC011603.246	17662	22571
MDP0000312336	*Malus domestica*	MDC019715.103	12727	13251
MDP0000340025	*Malus domestica*	MDC018226.184	4546	4968
MDP0000346969	*Malus domestica*	MDC016797.262	5451	5993
MDP0000348190	*Malus domestica*	MDC001315.773	19301	19915
MDP0000467241	*Malus domestica*	MDC017708.244	8442	9785
MDP0000478954	*Malus domestica*	MDC009316.433	10686	11393
MDP0000514448	*Malus domestica*	MDC006191.181	3706	4305
MDP0000564427	*Malus domestica*	MDC005944.592	9238	10032
MDP0000566117	*Malus domestica*	MDC004936.114	898	11600
MDP0000624279	*Malus domestica*	MDC005778.440	253	801
MDP0000713750	*Malus domestica*	MDC006022.710	6795	7361
MDP0000772371	*Malus domestica*	MDC008280.313	31	1014
MDP0000805273	*Malus domestica*	MDC000200.352	1867	2619
MDP0000833431	*Malus domestica*	MDC008272.442	1011	1418
MDP0000856686	*Malus domestica*	MDC019799.274	25720	26205
MDP0000868063	*Malus domestica*	MDC020164.56	3434	4369
MDP0000881505	*Malus domestica*	MDC018041.236	13616	14368
MDP0000885511	*Malus domestica*	MDC002975.216	22993	23382
MDP0000893506	*Malus domestica*	MDC010932.713	30969	31739
MDP0000925584	*Malus domestica*	MDC000987.661	12685	13275
Medtr1g028910	*Medicago truncatula*	chr1	9847443	9848042
Medtr1g028920	*Medicago truncatula*	chr1	9855883	9856191
Medtr1g054055	*Medicago truncatula*	chr1	23040618	23041237
Medtr1g110570	*Medicago truncatula*	chr1	49891664	49892623
Medtr2g013950	*Medicago truncatula*	chr2	3839731	3840815
Medtr2g019320	*Medicago truncatula*	chr2	6269503	6270082
Medtr2g035850	*Medicago truncatula*	chr2	15214004	15215247
Medtr2g061720	*Medicago truncatula*	chr2	26182404	26183236
Medtr2g070550	*Medicago truncatula*	chr2	29736749	29737159
Medtr2g079120	*Medicago truncatula*	chr2	33154057	33155261
Medtr3g013980	*Medicago truncatula*	chr3	3851224	3851618
Medtr3g081500	*Medicago truncatula*	chr3	36904409	36905059
Medtr3g087390	*Medicago truncatula*	chr3	39635830	39638412

Table S2

Medtr3g090350	*Medicago truncatula*	chr3	41104477	41105804
Medtr3g099400	*Medicago truncatula*	chr3	45563608	45565108
Medtr4g009950	*Medicago truncatula*	chr4	2137715	2138666
Medtr4g088695	*Medicago truncatula*	chr4	35302060	35303299
Medtr4g094698	*Medicago truncatula*	chr4	38752751	38753724
Medtr4g097280	*Medicago truncatula*	chr4	40092375	40093365
Medtr4g097350	*Medicago truncatula*	chr4	40121292	40122380
Medtr4g097360	*Medicago truncatula*	chr4	40124928	40125519
Medtr5g015190	*Medicago truncatula*	chr5	5227392	5228070
Medtr5g030570	*Medicago truncatula*	chr5	12954070	12957762
Medtr5g030570	*Medicago truncatula*	chr5	12954070	12958319
Medtr5g063310	*Medicago truncatula*	chr5	26256801	26258739
Medtr6g041990	*Medicago truncatula*	chr6	14496963	14497256
Medtr6g042010	*Medicago truncatula*	chr6	14504568	14504918
Medtr6g084200	*Medicago truncatula*	chr6	31464916	31465653
Medtr7g088620	*Medicago truncatula*	chr7	34541954	34543335
Medtr7g089860	*Medicago truncatula*	chr7	35219206	35219959
Medtr7g115420	*Medicago truncatula*	chr7	47688748	47689116
Medtr8g040080	*Medicago truncatula*	chr8	14917344	14917700
Medtr8g090290	*Medicago truncatula*	chr8	37941341	37942366
Medtr8g093335	*Medicago truncatula*	chr8	38970208	38971355
Medtr8g093390	*Medicago truncatula*	chr8	39001408	39002292
Medtr8g095470	*Medicago truncatula*	chr8	39955742	39957108
Medtr8g095470	*Medicago truncatula*	chr8	39955693	39958398
Medtr8g095470	*Medicago truncatula*	chr8	39955742	39958827
Migut.A00386	*Mimulus guttatus*	scaffold_1	2005141	2005608
Migut.B00886	*Mimulus guttatus*	scaffold_2	5800545	5801109
Migut.B01240	*Mimulus guttatus*	scaffold_2	14580495	14580941
Migut.B01401	*Mimulus guttatus*	scaffold_2	16126833	16127321
Migut.C00264	*Mimulus guttatus*	scaffold_3	5826815	5827786
Migut.C01025	*Mimulus guttatus*	scaffold_3	16975271	16976076
Migut.E00932	*Mimulus guttatus*	scaffold_5	10279210	10280160
Migut.H00333	*Mimulus guttatus*	scaffold_8	1994872	1995357
Migut.H00745	*Mimulus guttatus*	scaffold_8	5062540	5063686
Migut.H01482	*Mimulus guttatus*	scaffold_8	16284273	16285407
Migut.H02364	*Mimulus guttatus*	scaffold_8	23762720	23764010
Migut.H02540	*Mimulus guttatus*	scaffold_8	24826627	24827959
Migut.I00745	*Mimulus guttatus*	scaffold_9	12543834	12544512
Migut.J00487	*Mimulus guttatus*	scaffold_10	2456535	2457323
Migut.J01607	*Mimulus guttatus*	scaffold_10	17680453	17681590
Migut.J01657	*Mimulus guttatus*	scaffold_10	18011997	18012899
Migut.J01831	*Mimulus guttatus*	scaffold_10	19032286	19032856
Migut.K00136	*Mimulus guttatus*	scaffold_11	610559	611035
Migut.K00167	*Mimulus guttatus*	scaffold_11	733210	734100
Migut.L00113	*Mimulus guttatus*	scaffold_12	795520	796799
Migut.L00423	*Mimulus guttatus*	scaffold_12	5751968	5752534
Migut.L00424	*Mimulus guttatus*	scaffold_12	5756380	5756955
Migut.L01057	*Mimulus guttatus*	scaffold_12	20996028	20996546
Migut.M00092	*Mimulus guttatus*	scaffold_13	545669	546629
Migut.M00580	*Mimulus guttatus*	scaffold_13	11001913	11002470
Migut.M00647	*Mimulus guttatus*	scaffold_13	14059183	14059509
Migut.M00648	*Mimulus guttatus*	scaffold_13	14076865	14077185
Migut.M00649	*Mimulus guttatus*	scaffold_13	14078268	14078515
Migut.M00651	*Mimulus guttatus*	scaffold_13	14126605	14126919
Migut.M00652	*Mimulus guttatus*	scaffold_13	14128631	14128903
Migut.M00799	*Mimulus guttatus*	scaffold_13	15330447	15331172
Migut.M01133	*Mimulus guttatus*	scaffold_13	17086516	17087660
Migut.M02002	*Mimulus guttatus*	scaffold_13	21116339	21117061

Table S2

Migut.N01656	*Mimulus guttatus*	scaffold_14	14400527	14401947
Migut.N01854	*Mimulus guttatus*	scaffold_14	15686810	15687238
Migut.N02882	*Mimulus guttatus*	scaffold_14	23939619	23941059
orange1.1g012250m.g	*Citrus sinensis*	scaffold00099	255740	257628
orange1.1g014412m.g	*Citrus sinensis*	scaffold00004	1445342	1446619
orange1.1g021088m.g	*Citrus sinensis*	scaffold00004	1768999	1771843
orange1.1g021088m.g	*Citrus sinensis*	scaffold00004	1768999	1771843
orange1.1g021088m.g	*Citrus sinensis*	scaffold00004	1768999	1771843
orange1.1g021088m.g	*Citrus sinensis*	scaffold00004	1768999	1771843
orange1.1g026455m.g	*Citrus sinensis*	scaffold00091	343444	344588
orange1.1g026721m.g	*Citrus sinensis*	scaffold00036	854056	855601
orange1.1g027415m.g	*Citrus sinensis*	scaffold00078	412770	413682
orange1.1g027535m.g	*Citrus sinensis*	scaffold00305	158073	158858
orange1.1g029381m.g	*Citrus sinensis*	scaffold00006	800521	801275
orange1.1g030720m.g	*Citrus sinensis*	scaffold00074	423159	425823
orange1.1g030720m.g	*Citrus sinensis*	scaffold00074	422882	425823
orange1.1g031928m.g	*Citrus sinensis*	scaffold00553	37694	38260
orange1.1g033523m.g	*Citrus sinensis*	scaffold00007	1650471	1651115
orange1.1g038337m.g	*Citrus sinensis*	scaffold00008	574065	574604
orange1.1g039986m.g	*Citrus sinensis*	scaffold00008	1373926	1374606
orange1.1g040034m.g	*Citrus sinensis*	scaffold00025	847904	848368
orange1.1g041172m.g	*Citrus sinensis*	scaffold00072	99012	99437
orange1.1g041571m.g	*Citrus sinensis*	scaffold00339	16227	17234
orange1.1g044039m.g	*Citrus sinensis*	scaffold00356	60866	61252
orange1.1g044113m.g	*Citrus sinensis*	scaffold00018	1679168	1679629
orange1.1g045610m.g	*Citrus sinensis*	scaffold00002	483571	484431
Oropetium_20150105	*Oropetium thomaeum*	Oropetium_genomic_20141	6637087	6637560
Oropetium_20150105	*Oropetium thomaeum*	Oropetium_genomic_20141	6681508	6682122
Oropetium_20150105	*Oropetium thomaeum*	Oropetium_genomic_20141	2816044	2816649
Oropetium_20150105	*Oropetium thomaeum*	Oropetium_genomic_20141	3494293	3494766
Oropetium_20150105	*Oropetium thomaeum*	Oropetium_genomic_20141	5800605	5801903
Oropetium_20150105	*Oropetium thomaeum*	Oropetium_genomic_20141	562993	563580
Oropetium_20150105	*Oropetium thomaeum*	Oropetium_genomic_20141	1392192	1392935
Oropetium_20150105	*Oropetium thomaeum*	Oropetium_genomic_20141	2703470	2707164
Oropetium_20150105	*Oropetium thomaeum*	Oropetium_genomic_20141	840858	841466
Oropetium_20150105	*Oropetium thomaeum*	Oropetium_genomic_20141	2807365	2808639
Oropetium_20150105	*Oropetium thomaeum*	Oropetium_genomic_20141	2350787	2351245
Oropetium_20150105	*Oropetium thomaeum*	Oropetium_genomic_20141	1576636	1577007
Oropetium_20150105	*Oropetium thomaeum*	Oropetium_genomic_20141	810937	813717
Oropetium_20150105	*Oropetium thomaeum*	Oropetium_genomic_20141	270780	271682
Oropetium_20150105	*Oropetium thomaeum*	Oropetium_genomic_20141	2346176	2346871
Oropetium_20150105	*Oropetium thomaeum*	Oropetium_genomic_20141	3620718	3621368
Oropetium_20150105	*Oropetium thomaeum*	Oropetium_genomic_20141	2237915	2238805
Oropetium_20150105	*Oropetium thomaeum*	Oropetium_genomic_20141	1692174	1692410
Oropetium_20150105	*Oropetium thomaeum*	Oropetium_genomic_20141	717098	717829
Oropetium_20150105	*Oropetium thomaeum*	Oropetium_genomic_20141	97682	98425
Oropetium_20150105	*Oropetium thomaeum*	Oropetium_genomic_20141	678267	678950
Oropetium_20150105	*Oropetium thomaeum*	Oropetium_genomic_20141	145652	146221
Oropetium_20150105	*Oropetium thomaeum*	Oropetium_genomic_20141	571880	572548
Oropetium_20150105	*Oropetium thomaeum*	Oropetium_genomic_20141	1981958	1982356
Oropetium_20150105	*Oropetium thomaeum*	Oropetium_genomic_20141	1412473	1413126
Oropetium_20150105	*Oropetium thomaeum*	Oropetium_genomic_20141	1758651	1759406
Oropetium_20150105	*Oropetium thomaeum*	Oropetium_genomic_20141	172611	173288
Oropetium_20150105	*Oropetium thomaeum*	Oropetium_genomic_20141	470549	471124
Oropetium_20150105	*Oropetium thomaeum*	Oropetium_genomic_20141	1384288	1384593
Oropetium_20150105	*Oropetium thomaeum*	Oropetium_genomic_20141	1018784	1019611
Oropetium_20150105	*Oropetium thomaeum*	Oropetium_genomic_20141	1573314	1573721
Oropetium_20150105	*Oropetium thomaeum*	Oropetium_genomic_20141	254710	255075

Table S2

Oropetium_2015010	*Oropetium thomaeum*	Oropetium_genomic_20141	105432	105884
Oropetium_2015010	*Oropetium thomaeum*	Oropetium_genomic_20141	1976	2527
Oropetium_2015010	*Oropetium thomaeum*	Oropetium_genomic_20141	90187	90681
Oropetium_2015010	*Oropetium thomaeum*	Oropetium_genomic_20141	35017	35580
Oropetium_2015010	*Oropetium thomaeum*	Oropetium_genomic_20141	15888	16346
Oropetium_2015010	*Oropetium thomaeum*	Oropetium_genomic_20141	502	1085
Oropetium_2015010	*Oropetium thomaeum*	Oropetium_genomic_20141	25360	26010
PGSC0003DMG4000	*Solanum tuberosum*	ST4.03ch04	58797800	58798630
PGSC0003DMG4000	*Solanum tuberosum*	ST4.03ch11	6423951	6424259
PGSC0003DMG4000	*Solanum tuberosum*	ST4.03ch04	51268311	51269672
PGSC0003DMG4000	*Solanum tuberosum*	ST4.03ch06	44749122	44750159
PGSC0003DMG4000	*Solanum tuberosum*	ST4.03ch06	44749122	44750159
PGSC0003DMG4000	*Solanum tuberosum*	ST4.03ch09	4200101	4200778
PGSC0003DMG4000	*Solanum tuberosum*	ST4.03ch02	22625866	22626681
PGSC0003DMG4000	*Solanum tuberosum*	ST4.03ch01	74238678	74239172
PGSC0003DMG4000	*Solanum tuberosum*	ST4.03ch03	58945995	58946483
PGSC0003DMG4000	*Solanum tuberosum*	ST4.03ch10	59556146	59556781
PGSC0003DMG4000	*Solanum tuberosum*	ST4.03ch12	1092570	1102551
PGSC0003DMG4000	*Solanum tuberosum*	ST4.03ch10	59222091	59222519
PGSC0003DMG4000	*Solanum tuberosum*	ST4.03ch10	59222091	59222423
PGSC0003DMG4000	*Solanum tuberosum*	ST4.03ch12	55283358	55284344
PGSC0003DMG4000	*Solanum tuberosum*	ST4.03ch12	55283358	55284344
PGSC0003DMG4000	*Solanum tuberosum*	ST4.03ch02	27170481	27171077
PGSC0003DMG4000	*Solanum tuberosum*	ST4.03ch12	56317475	56317915
PGSC0003DMG4000	*Solanum tuberosum*	ST4.03ch07	41503400	41503873
PGSC0003DMG4000	*Solanum tuberosum*	ST4.03ch07	41503400	41503936
PGSC0003DMG4000	*Solanum tuberosum*	ST4.03ch09	4273386	4273997
PGSC0003DMG4000	*Solanum tuberosum*	ST4.03ch09	4290266	4290805
PGSC0003DMG4000	*Solanum tuberosum*	ST4.03ch03	57317009	57317509
PGSC0003DMG4000	*Solanum tuberosum*	ST4.03ch02	15572377	15572964
PGSC0003DMG4000	*Solanum tuberosum*	ST4.03ch06	43419764	43421381
PGSC0003DMG4000	*Solanum tuberosum*	ST4.03ch06	43419764	43420501
PGSC0003DMG4000	*Solanum tuberosum*	ST4.03ch04	62977420	62978793
PGSC0003DMG4000	*Solanum tuberosum*	ST4.03ch05	50792878	50793213
PGSC0003DMG4000	*Solanum tuberosum*	ST4.03ch10	57492633	57493154
PGSC0003DMG4000	*Solanum tuberosum*	ST4.03ch04	5449162	5449431
PGSC0003DMG4000	*Solanum tuberosum*	ST4.03ch04	5446059	5446337
PGSC0003DMG4000	*Solanum tuberosum*	ST4.03ch07	51841107	51841430
PGSC0003DMG4000	*Solanum tuberosum*	ST4.03ch10	56872795	56873466
PGSC0003DMG4000	*Solanum tuberosum*	ST4.03ch12	49719801	49720548
PGSC0003DMG4000	*Solanum tuberosum*	ST4.03ch12	49719801	49720133
PGSC0003DMG4000	*Solanum tuberosum*	ST4.03ch11	6991798	6992487
PGSC0003DMG4000	*Solanum tuberosum*	ST4.03ch01	66962853	66963320
PGSC0003DMG4000	*Solanum tuberosum*	ST4.03ch02	33798135	33798923
PGSC0003DMG4000	*Solanum tuberosum*	ST4.03ch10	2698123	2698842
PGSC0003DMG4000	*Solanum tuberosum*	ST4.03ch08	49631734	49632849
PGSC0003DMG4000	*Solanum tuberosum*	ST4.03ch07	53133434	53134126
PGSC0003DMG4000	*Solanum tuberosum*	ST4.03ch07	51242374	51242880
PGSC0003DMG4000	*Solanum tuberosum*	ST4.03ch04	64029228	64030193
PGSC0003DMG4010	*Solanum tuberosum*	ST4.03ch06	50793226	50794152
Potri.001G029700	*Populus trichocarpa*	Chr01	2186522	2187295
Potri.001G130300	*Populus trichocarpa*	Chr01	10520583	10520941
Potri.001G142400	*Populus trichocarpa*	Chr01	11493369	11494939
Potri.001G158800	*Populus trichocarpa*	Chr01	13225758	13226690
Potri.001G230800	*Populus trichocarpa*	Chr01	24286048	24286941
Potri.001G230800	*Populus trichocarpa*	Chr01	24286048	24286941
Potri.001G378300	*Populus trichocarpa*	Chr01	39400955	39401393
Potri.001G399100	*Populus trichocarpa*	Chr01	42015598	42016722

Table S2

Potri.002G070600	*Populus trichocarpa*	Chr02	4877302	4878531
Potri.002G094900	*Populus trichocarpa*	Chr02	6835088	6837213
Potri.002G099900	*Populus trichocarpa*	Chr02	7209578	7211686
Potri.003G075800	*Populus trichocarpa*	Chr03	10433665	10434499
Potri.003G091800	*Populus trichocarpa*	Chr03	11906118	11908575
Potri.003G194700	*Populus trichocarpa*	Chr03	19815407	19816443
Potri.004G044800	*Populus trichocarpa*	Chr04	3413662	3414922
Potri.004G071500	*Populus trichocarpa*	Chr04	6001274	6001710
Potri.004G134200	*Populus trichocarpa*	Chr04	15472198	15472737
Potri.005G057800	*Populus trichocarpa*	Chr05	4066011	4067266
Potri.005G076600	*Populus trichocarpa*	Chr05	5625322	5627730
Potri.005G162300	*Populus trichocarpa*	Chr05	16607608	16609452
Potri.005G166100	*Populus trichocarpa*	Chr05	17260773	17263580
Potri.005G189300	*Populus trichocarpa*	Chr05	20689820	20690840
Potri.005G204000	*Populus trichocarpa*	Chr05	21881226	21882935
Potri.006G006300	*Populus trichocarpa*	Chr06	438132	439307
Potri.006G032300	*Populus trichocarpa*	Chr06	2192817	2194363
Potri.006G038900	*Populus trichocarpa*	Chr06	2741633	2743465
Potri.006G192100	*Populus trichocarpa*	Chr06	20687841	20688371
Potri.006G199300	*Populus trichocarpa*	Chr06	21444923	21445471
Potri.006G266600	*Populus trichocarpa*	Chr06	26863353	26864293
Potri.006G266700	*Populus trichocarpa*	Chr06	26867519	26869778
Potri.007G006200	*Populus trichocarpa*	Chr07	440560	441141
Potri.007G091600	*Populus trichocarpa*	Chr07	11851318	11852980
Potri.007G110100	*Populus trichocarpa*	Chr07	13325551	13326901
Potri.009G024200	*Populus trichocarpa*	Chr09	3572517	3573629
Potri.010G123700	*Populus trichocarpa*	Chr10	13998861	13999918
Potri.011G053700	*Populus trichocarpa*	Chr11	4668383	4669684
Potri.011G084800	*Populus trichocarpa*	Chr11	8950138	8950638
Potri.011G095900	*Populus trichocarpa*	Chr11	11689143	11689560
Potri.011G118200	*Populus trichocarpa*	Chr11	14358167	14359731
Potri.011G118200	*Populus trichocarpa*	Chr11	14356562	14359731
Potri.011G118200	*Populus trichocarpa*	Chr11	14356562	14359731
Potri.012G055900	*Populus trichocarpa*	Chr12	5752301	5753139
Potri.013G043800	*Populus trichocarpa*	Chr13	3094146	3094553
Potri.014G006400	*Populus trichocarpa*	Chr14	703700	704197
Potri.014G008600	*Populus trichocarpa*	Chr14	879088	879675
Potri.014G141600	*Populus trichocarpa*	Chr14	10776134	10776691
Potri.015G046600	*Populus trichocarpa*	Chr15	4689835	4690437
Potri.016G017900	*Populus trichocarpa*	Chr16	958255	959444
Potri.016G029600	*Populus trichocarpa*	Chr16	1676014	1677622
Potri.016G036600	*Populus trichocarpa*	Chr16	2182480	2184539
Potri.016G046000	*Populus trichocarpa*	Chr16	2901669	2902513
Potri.016G093900	*Populus trichocarpa*	Chr16	8101978	8102394
Potri.018G016500	*Populus trichocarpa*	Chr18	1292423	1293612
Potri.019G013300	*Populus trichocarpa*	Chr19	1510650	1511545
Pp3c1_9790	*Physcomitrella patens*	Chr01	7133824	7136789
Pp3c1_9790	*Physcomitrella patens*	Chr01	7133324	7136789
Pp3c1_9790	*Physcomitrella patens*	Chr01	7133324	7136764
Pp3c1_9790	*Physcomitrella patens*	Chr01	7133324	7136769
Pp3c1_9790	*Physcomitrella patens*	Chr01	7133324	7136764
Pp3c1_9790	*Physcomitrella patens*	Chr01	7133324	7136769
Pp3c1_9790	*Physcomitrella patens*	Chr01	7133807	7136212
Pp3c1_9790	*Physcomitrella patens*	Chr01	7133822	7136764
Pp3c1_9790	*Physcomitrella patens*	Chr01	7133824	7136764
Pp3c11_25170	*Physcomitrella patens*	Chr11	16415847	16418347
Pp3c11_25170	*Physcomitrella patens*	Chr11	16415812	16418296
Pp3c11_25170	*Physcomitrella patens*	Chr11	16415821	16418347

Table S2

Pp3c15_15270	*Physcomitrella patens*	Chr15	9951739	9954559
Pp3c15_15270	*Physcomitrella patens*	Chr15	9951561	9954559
Pp3c15_15270	*Physcomitrella patens*	Chr15	9951670	9954575
Pp3c15_19310	*Physcomitrella patens*	Chr15	12715520	12717429
Pp3c15_19310	*Physcomitrella patens*	Chr15	12715520	12717747
Pp3c16_10920	*Physcomitrella patens*	Chr16	6901776	6903218
Pp3c16_10920	*Physcomitrella patens*	Chr16	6901648	6903149
Pp3c16_19040	*Physcomitrella patens*	Chr16	11638746	11641402
Pp3c16_19040	*Physcomitrella patens*	Chr16	11638738	11641423
Pp3c16_19220	*Physcomitrella patens*	Chr16	11743969	11745864
Pp3c16_19220	*Physcomitrella patens*	Chr16	11743935	11745816
Pp3c16_22640	*Physcomitrella patens*	Chr16	14076141	14079066
Pp3c16_22640	*Physcomitrella patens*	Chr16	14075785	14079696
Pp3c16_22640	*Physcomitrella patens*	Chr16	14075785	14079770
Pp3c16_22640	*Physcomitrella patens*	Chr16	14075785	14079696
Pp3c2_38120	*Physcomitrella patens*	Chr02	25825213	25828059
Pp3c2_38120	*Physcomitrella patens*	Chr02	25825203	25827985
Pp3c2_38120	*Physcomitrella patens*	Chr02	25825203	25827939
Pp3c2_38120	*Physcomitrella patens*	Chr02	25825203	25827960
Pp3c20_1460	*Physcomitrella patens*	Chr20	757984	760731
Pp3c20_1460	*Physcomitrella patens*	Chr20	757832	760687
Pp3c23_18320	*Physcomitrella patens*	Chr23	12097590	12100236
Pp3c23_18320	*Physcomitrella patens*	Chr23	12096936	12100236
Pp3c23_18320	*Physcomitrella patens*	Chr23	12096986	12100236
Pp3c23_18320	*Physcomitrella patens*	Chr23	12097571	12100236
Pp3c23_18320	*Physcomitrella patens*	Chr23	12097584	12100235
Pp3c23_18340	*Physcomitrella patens*	Chr23	12103392	12105298
Pp3c23_18340	*Physcomitrella patens*	Chr23	12103019	12105733
Pp3c25_12310	*Physcomitrella patens*	Chr25	8748177	8749617
Pp3c25_12310	*Physcomitrella patens*	Chr25	8747370	8750161
Pp3c25_12310	*Physcomitrella patens*	Chr25	8747370	8750161
Pp3c25_12370	*Physcomitrella patens*	Chr25	8776415	8777317
Pp3c25_3350	*Physcomitrella patens*	Chr25	2215159	2216642
Pp3c25_3350	*Physcomitrella patens*	Chr25	2214550	2217384
Pp3c25_3350	*Physcomitrella patens*	Chr25	2214550	2217384
Pp3c25_9290	*Physcomitrella patens*	Chr25	6469594	6470822
Pp3c25_9290	*Physcomitrella patens*	Chr25	6469562	6470826
Pp3c5_24370	*Physcomitrella patens*	Chr05	17056390	17058920
Pp3c5_24370	*Physcomitrella patens*	Chr05	17056343	17058707
Pp3c5_24450	*Physcomitrella patens*	Chr05	17085812	17088401
Pp3c5_25320	*Physcomitrella patens*	Chr05	17800561	17803281
Pp3c5_25320	*Physcomitrella patens*	Chr05	17799429	17802285
Pp3c5_25320	*Physcomitrella patens*	Chr05	17799429	17802285
Pp3c5_25320	*Physcomitrella patens*	Chr05	17799954	17802286
Pp3c5_25320	*Physcomitrella patens*	Chr05	17799954	17802274
Pp3c5_25320	*Physcomitrella patens*	Chr05	17799954	17802285
Pp3c5_25320	*Physcomitrella patens*	Chr05	17799954	17802285
Pp3c5_25320	*Physcomitrella patens*	Chr05	17799954	17802276
Pp3c5_25420	*Physcomitrella patens*	Chr05	17931059	17932450
Pp3c5_25420	*Physcomitrella patens*	Chr05	17931059	17932358
Pp3c6_4170	*Physcomitrella patens*	Chr06	2473222	2475659
Pp3c6_4170	*Physcomitrella patens*	Chr06	2473192	2475781
Pp3c6_7300	*Physcomitrella patens*	Chr06	4337344	4337769
Pp3c6_7300	*Physcomitrella patens*	Chr06	4336988	4338305
Pp3c6_7340	*Physcomitrella patens*	Chr06	4390256	4391854
Pp3c6_7340	*Physcomitrella patens*	Chr06	4390256	4391792
Pp3c6_7340	*Physcomitrella patens*	Chr06	4390256	4391930
Pp3c6_7340	*Physcomitrella patens*	Chr06	4390256	4391930

Table S2

Pp3c7_13780	*Physcomitrella patens*	Chr07	9344828	9347998
Pp3c7_13780	*Physcomitrella patens*	Chr07	9344827	9348000
Pp3c7_13780	*Physcomitrella patens*	Chr07	9344828	9348002
Pp3c7_13780	*Physcomitrella patens*	Chr07	9344828	9347998
Pp3c7_13780	*Physcomitrella patens*	Chr07	9344828	9347998
Pp3c7_7100	*Physcomitrella patens*	Chr07	4443698	4445556
Pp3c7_7100	*Physcomitrella patens*	Chr07	4442859	4445393
Pp3c8_22400	*Physcomitrella patens*	Chr08	15405228	15408157
Pp3c8_22400	*Physcomitrella patens*	Chr08	15405148	15408183
Pp3c8_22400	*Physcomitrella patens*	Chr08	15405207	15408166
Pp3c8_22400	*Physcomitrella patens*	Chr08	15405207	15408157
Pp3c8_22400	*Physcomitrella patens*	Chr08	15405207	15408136
Pp3c8_22400	*Physcomitrella patens*	Chr08	15405207	15408000
Pp3c8_22400	*Physcomitrella patens*	Chr08	15405207	15408136
Pp3c8_22400	*Physcomitrella patens*	Chr08	15405207	15408140
Pp3c8_22400	*Physcomitrella patens*	Chr08	15405207	15408136
Pp3c9_19950	*Physcomitrella patens*	Chr09	13447810	13450561
Pp3c9_19950	*Physcomitrella patens*	Chr09	13447787	13450625
Prupe.1G312500	*Prunus persica*	Pp01	30310330	30311534
Prupe.1G396600	*Prunus persica*	Pp01	35157071	35159007
Prupe.1G514600	*Prunus persica*	Pp01	42345600	42347900
Prupe.1G522100	*Prunus persica*	Pp01	42787397	42791235
Prupe.1G550700	*Prunus persica*	Pp01	45006928	45010323
Prupe.1G550700	*Prunus persica*	Pp01	45005025	45015615
Prupe.1G550700	*Prunus persica*	Pp01	45005025	45015612
Prupe.1G550700	*Prunus persica*	Pp01	45005025	45015706
Prupe.1G550700	*Prunus persica*	Pp01	45005025	45015750
Prupe.1G550700	*Prunus persica*	Pp01	45005025	45015722
Prupe.1G550700	*Prunus persica*	Pp01	45005025	45015612
Prupe.1G550700	*Prunus persica*	Pp01	45006060	45015612
Prupe.1G550700	*Prunus persica*	Pp01	45006094	45009795
Prupe.1G550700	*Prunus persica*	Pp01	45006150	45015630
Prupe.1G550700	*Prunus persica*	Pp01	45006240	45015172
Prupe.1G550700	*Prunus persica*	Pp01	45006240	45015172
Prupe.1G550700	*Prunus persica*	Pp01	45006241	45015706
Prupe.1G550700	*Prunus persica*	Pp01	45006242	45015582
Prupe.1G550700	*Prunus persica*	Pp01	45006251	45015750
Prupe.1G550700	*Prunus persica*	Pp01	45006251	45015582
Prupe.2G000100	*Prunus persica*	Pp02	93474	94791
Prupe.2G002600	*Prunus persica*	Pp02	325286	326279
Prupe.2G041200	*Prunus persica*	Pp02	4528127	4529124
Prupe.3G091700	*Prunus persica*	Pp03	6831873	6833100
Prupe.3G175400	*Prunus persica*	Pp03	19224514	19225499
Prupe.3G185800	*Prunus persica*	Pp03	20034265	20035525
Prupe.4G048900	*Prunus persica*	Pp04	2336709	2337451
Prupe.4G116000	*Prunus persica*	Pp04	6273009	6274145
Prupe.4G149000	*Prunus persica*	Pp04	8510604	8512604
Prupe.4G180100	*Prunus persica*	Pp04	10679240	10680055
Prupe.4G286700	*Prunus persica*	Pp04	25146373	25146954
Prupe.5G036600	*Prunus persica*	Pp05	4155069	4158442
Prupe.5G036600	*Prunus persica*	Pp05	4155069	4158442
Prupe.5G036600	*Prunus persica*	Pp05	4155069	4158442
Prupe.5G222000	*Prunus persica*	Pp05	17263929	17264977
Prupe.6G183200	*Prunus persica*	Pp06	19037459	19040864
Prupe.6G183200	*Prunus persica*	Pp06	19037459	19040864
Prupe.6G218200	*Prunus persica*	Pp06	22485430	22486482
Prupe.6G218300	*Prunus persica*	Pp06	22498702	22499601
Prupe.6G310500	*Prunus persica*	Pp06	27841446	27842672

Table S2

Prupe.6G347000	*Prunus persica*	Pp06	29707539	29707919
Prupe.7G038200	*Prunus persica*	Pp07	7097581	7098362
Prupe.7G076600	*Prunus persica*	Pp07	11329895	11330935
Prupe.8G022500	*Prunus persica*	Pp08	2023441	2025113
Seita.1G015300	*Setaria italica*	scaffold_1	1341648	1342593
Seita.1G015400	*Setaria italica*	scaffold_1	1346277	1346633
Seita.1G070200	*Setaria italica*	scaffold_1	6462653	6466293
Seita.1G185200	*Setaria italica*	scaffold_1	26627116	26629147
Seita.1G324200	*Setaria italica*	scaffold_1	38372178	38373103
Seita.2G039200	*Setaria italica*	scaffold_2	3233035	3233796
Seita.2G188800	*Setaria italica*	scaffold_2	28482730	28484079
Seita.2G396000	*Setaria italica*	scaffold_2	46041864	46043403
Seita.2G439500	*Setaria italica*	scaffold_2	48840569	48841763
Seita.3G007100	*Setaria italica*	scaffold_3	375158	375944
Seita.3G029500	*Setaria italica*	scaffold_3	1806182	1806655
Seita.3G075700	*Setaria italica*	scaffold_3	4819529	4820346
Seita.3G138400	*Setaria italica*	scaffold_3	9638909	9645164
Seita.3G176500	*Setaria italica*	scaffold_3	13094775	13095521
Seita.3G195500	*Setaria italica*	scaffold_3	14865245	14866371
Seita.3G404200	*Setaria italica*	scaffold_3	50302942	50303310
Seita.4G067700	*Setaria italica*	scaffold_4	5222077	5222460
Seita.4G169300	*Setaria italica*	scaffold_4	26904286	26905224
Seita.4G214500	*Setaria italica*	scaffold_4	33221018	33222383
Seita.4G223000	*Setaria italica*	scaffold_4	34348206	34349526
Seita.4G268900	*Setaria italica*	scaffold_4	38604982	38606063
Seita.5G040900	*Setaria italica*	scaffold_5	3725366	3725950
Seita.5G142700	*Setaria italica*	scaffold_5	12571458	12572120
Seita.5G247300	*Setaria italica*	scaffold_5	30883175	30883647
Seita.5G316700	*Setaria italica*	scaffold_5	36760934	36764278
Seita.5G354000	*Setaria italica*	scaffold_5	39570825	39572070
Seita.6G003100	*Setaria italica*	scaffold_6	230417	231199
Seita.6G141800	*Setaria italica*	scaffold_6	25319447	25321399
Seita.7G094400	*Setaria italica*	scaffold_7	19741913	19743783
Seita.7G261300	*Setaria italica*	scaffold_7	31441226	31442868
Seita.7G309400	*Setaria italica*	scaffold_7	34606900	34608119
Seita.8G004900	*Setaria italica*	scaffold_8	296448	297847
Seita.9G062300	*Setaria italica*	scaffold_9	3581688	3582612
Seita.9G141000	*Setaria italica*	scaffold_9	9013344	9014725
Seita.9G302300	*Setaria italica*	scaffold_9	35017799	35018581
Seita.9G302500	*Setaria italica*	scaffold_9	35040088	35040763
Seita.9G310200	*Setaria italica*	scaffold_9	35815634	35816350
Seita.9G381000	*Setaria italica*	scaffold_9	44006973	44007809
Seita.9G422500	*Setaria italica*	scaffold_9	47861865	47862869
Seita.9G424100	*Setaria italica*	scaffold_9	47969554	47970534
Sobic.001G063200	*Sorghum bicolor*	Chr01	4718332	4719645
Sobic.001G138600	*Sorghum bicolor*	Chr01	11019649	11020808
Sobic.001G279300	*Sorghum bicolor*	Chr01	54539553	54540393
Sobic.001G279500	*Sorghum bicolor*	Chr01	54568947	54569768
Sobic.001G285000	*Sorghum bicolor*	Chr01	55824296	55825255
Sobic.001G287400	*Sorghum bicolor*	Chr01	56294761	56296031
Sobic.001G352700	*Sorghum bicolor*	Chr01	64183029	64183913
Sobic.001G389700	*Sorghum bicolor*	Chr01	67631769	67633355
Sobic.001G390700	*Sorghum bicolor*	Chr01	67707824	67710090
Sobic.001G475701	*Sorghum bicolor*	Chr01	74796033	74796776
Sobic.001G492600	*Sorghum bicolor*	Chr01	76193243	76196393
Sobic.001G492600	*Sorghum bicolor*	Chr01	76183043	76196501
Sobic.001G492600	*Sorghum bicolor*	Chr01	76183048	76196501
Sobic.001G492600	*Sorghum bicolor*	Chr01	76183069	76196393

Table S2

Sobic.001G492600	*Sorghum bicolor*	Chr01	76193019	76196501
Sobic.002G040900	*Sorghum bicolor*	Chr02	3944279	3945265
Sobic.002G185200	*Sorghum bicolor*	Chr02	56923688	56928881
Sobic.002G185200	*Sorghum bicolor*	Chr02	56923688	56928881
Sobic.002G381300	*Sorghum bicolor*	Chr02	73712952	73714246
Sobic.002G425000	*Sorghum bicolor*	Chr02	77174919	77175533
Sobic.003G027400	*Sorghum bicolor*	Chr03	2408735	2410667
Sobic.003G131500	*Sorghum bicolor*	Chr03	12322802	12323668
Sobic.003G240600	*Sorghum bicolor*	Chr03	57961107	57962017
Sobic.003G294400	*Sorghum bicolor*	Chr03	62677714	62679117
Sobic.003G329700	*Sorghum bicolor*	Chr03	65497419	65503103
Sobic.004G111800	*Sorghum bicolor*	Chr04	11036312	11037382
Sobic.004G232400	*Sorghum bicolor*	Chr04	58136911	58138552
Sobic.005G021950	*Sorghum bicolor*	Chr05	2015176	2015814
Sobic.006G075500	*Sorghum bicolor*	Chr06	43980789	43981892
Sobic.006G239100	*Sorghum bicolor*	Chr06	57985251	57987615
Sobic.006G256700	*Sorghum bicolor*	Chr06	59330329	59331263
Sobic.006G275300	*Sorghum bicolor*	Chr06	60646112	60647075
Sobic.007G002100	*Sorghum bicolor*	Chr07	218555	220436
Sobic.007G124301	*Sorghum bicolor*	Chr07	53279013	53281524
Sobic.008G018400	*Sorghum bicolor*	Chr08	1493728	1495198
Sobic.008G188000	*Sorghum bicolor*	Chr08	62233390	62234784
Sobic.008G188000	*Sorghum bicolor*	Chr08	62233390	62234783
Sobic.009G180550	*Sorghum bicolor*	Chr09	53477276	53477659
Sobic.009G200700	*Sorghum bicolor*	Chr09	55054074	55055883
Sobic.009G239700	*Sorghum bicolor*	Chr09	57753538	57753792
Sobic.010G045000	*Sorghum bicolor*	Chr10	3482938	3483360
Sobic.010G155300	*Sorghum bicolor*	Chr10	45701695	45702656
Sobic.010G185000	*Sorghum bicolor*	Chr10	52485614	52489444
Sobic.010G194300	*Sorghum bicolor*	Chr10	53688115	53688687
Sobic.010G221032	*Sorghum bicolor*	Chr10	56362136	56363551
Sobic.010G221032	*Sorghum bicolor*	Chr10	56362136	56363551
Solyc01g096510.2	*Solanum lycopersicum*	SL2.50ch01	87596368	87597121
Solyc02g031990.1	*Solanum lycopersicum*	SL2.50ch02	27259478	27260026
Solyc02g064570.1	*Solanum lycopersicum*	SL2.50ch02	35678099	35680533
Solyc02g068460.1	*Solanum lycopersicum*	SL2.50ch02	38401854	38402432
Solyc02g068470.1	*Solanum lycopersicum*	SL2.50ch02	38412522	38412989
Solyc02g078030.1	*Solanum lycopersicum*	SL2.50ch02	42843097	42843888
Solyc03g117500.1	*Solanum lycopersicum*	SL2.50ch03	66645395	66645901
Solyc03g119410.1	*Solanum lycopersicum*	SL2.50ch03	68034081	68034536
Solyc04g055050.1	*Solanum lycopersicum*	SL2.50ch04	53604965	53606995
Solyc04g073950.1	*Solanum lycopersicum*	SL2.50ch04	59963374	59964768
Solyc04g074520.1	*Solanum lycopersicum*	SL2.50ch04	60506623	60507594
Solyc06g060470.1	*Solanum lycopersicum*	SL2.50ch06	38497586	38498308
Solyc06g061190.1	*Solanum lycopersicum*	SL2.50ch06	39193327	39194433
Solyc06g069100.1	*Solanum lycopersicum*	SL2.50ch06	42886815	42891453
Solyc07g043250.1	*Solanum lycopersicum*	SL2.50ch07	56909878	56910360
Solyc07g056600.1	*Solanum lycopersicum*	SL2.50ch07	64413049	64413378
Solyc07g063070.1	*Solanum lycopersicum*	SL2.50ch07	65649614	65650315
Solyc09g008740.1	*Solanum lycopersicum*	SL2.50ch09	2170423	2170956
Solyc09g008750.1	*Solanum lycopersicum*	SL2.50ch09	2181098	2181706
Solyc09g008760.1	*Solanum lycopersicum*	SL2.50ch09	2190939	2191652
Solyc10g007580.1	*Solanum lycopersicum*	SL2.50ch10	1880180	1880425
Solyc10g077130.1	*Solanum lycopersicum*	SL2.50ch10	60003111	60003728
Solyc10g078440.1	*Solanum lycopersicum*	SL2.50ch10	60268059	60268484
Solyc11g005720.1	*Solanum lycopersicum*	SL2.50ch11	572324	573019
Solyc12g042730.1	*Solanum lycopersicum*	SL2.50ch12	39718257	39718775
Solyc12g088490.1	*Solanum lycopersicum*	SL2.50ch12	63836570	63837058

Table S2

Spipo12G0038500	*Spirodela polyrhiza*	pseudo12	2876960	2877436
Spipo12G0049100	*Spirodela polyrhiza*	pseudo12	3515818	3517319
Spipo13G0006800	*Spirodela polyrhiza*	pseudo13	354115	354903
Spipo15G0010600	*Spirodela polyrhiza*	pseudo15	931596	941062
Spipo18G0012100	*Spirodela polyrhiza*	pseudo18	1081521	1081814
Spipo18G0034700	*Spirodela polyrhiza*	pseudo18	2883258	2884235
Spipo19G0000400	*Spirodela polyrhiza*	pseudo19	24210	25019
Spipo21G0001000	*Spirodela polyrhiza*	pseudo21	110214	110738
Spipo24G0029600	*Spirodela polyrhiza*	pseudo24	2290244	2291044
Spipo29G0006300	*Spirodela polyrhiza*	pseudo29	461442	461858
Spipo2G0045300	*Spirodela polyrhiza*	pseudo2	3777527	3778405
Spipo3G0021200	*Spirodela polyrhiza*	pseudo3	1348393	1348890
Spipo3G0110800	*Spirodela polyrhiza*	pseudo3	8596307	8596774
Spipo4G0015800	*Spirodela polyrhiza*	pseudo4	1531542	1532285
Spipo4G0108900	*Spirodela polyrhiza*	pseudo4	8014277	8014942
Spipo5G0064400	*Spirodela polyrhiza*	pseudo5	5632264	5633232
Spipo7G0026800	*Spirodela polyrhiza*	pseudo7	2443441	2444073
Spipo7G0038000	*Spirodela polyrhiza*	pseudo7	3593248	3593835
Spipo9G0025500	*Spirodela polyrhiza*	pseudo9	1823291	1823731
Thecc1EG000826	*Theobroma cacao*	scaffold_1	3665568	3666433
Thecc1EG001135	*Theobroma cacao*	scaffold_1	5423498	5425872
Thecc1EG001406	*Theobroma cacao*	scaffold_1	6944558	6946248
Thecc1EG001671	*Theobroma cacao*	scaffold_1	8652393	8654890
Thecc1EG002034	*Theobroma cacao*	scaffold_1	11143089	11144353
Thecc1EG006012	*Theobroma cacao*	scaffold_2	274858	276288
Thecc1EG006014	*Theobroma cacao*	scaffold_2	283299	284298
Thecc1EG009707	*Theobroma cacao*	scaffold_2	26622742	26623950
Thecc1EG012360	*Theobroma cacao*	scaffold_3	2485917	2486851
Thecc1EG015718	*Theobroma cacao*	scaffold_3	29270881	29273305
Thecc1EG015718	*Theobroma cacao*	scaffold_3	29270857	29273318
Thecc1EG015718	*Theobroma cacao*	scaffold_3	29270870	29273425
Thecc1EG023150	*Theobroma cacao*	scaffold_5	10994582	11009245
Thecc1EG023799	*Theobroma cacao*	scaffold_5	18600636	18611220
Thecc1EG024241	*Theobroma cacao*	scaffold_5	23763691	23764366
Thecc1EG024904	*Theobroma cacao*	scaffold_5	29471037	29478144
Thecc1EG026260	*Theobroma cacao*	scaffold_5	37381493	37382865
Thecc1EG027281	*Theobroma cacao*	scaffold_6	2697791	2698810
Thecc1EG029475	*Theobroma cacao*	scaffold_6	22812011	22812585
Thecc1EG029847	*Theobroma cacao*	scaffold_6	24602683	24604298
Thecc1EG031539	*Theobroma cacao*	scaffold_7	5454561	5455635
Thecc1EG032332	*Theobroma cacao*	scaffold_7	10162333	10163717
Thecc1EG034861	*Theobroma cacao*	scaffold_8	5118475	5121469
Thecc1EG034928	*Theobroma cacao*	scaffold_8	5485111	5491034
Thecc1EG035487	*Theobroma cacao*	scaffold_8	9424134	9425316
Thecc1EG036879	*Theobroma cacao*	scaffold_9	668138	669443
Thecc1EG045032	*Theobroma cacao*	scaffold_10r	21868748	21869354
Thhalv10000717m.g	*Eutrema salsugineum*	scaffold_15	1429878	1430234
Thhalv10001010m.g	*Eutrema salsugineum*	scaffold_20	2033641	2034855
Thhalv10001232m.g	*Eutrema salsugineum*	scaffold_20	480616	481489
Thhalv10001743m.g	*Eutrema salsugineum*	scaffold_22	389789	390325
Thhalv10001873m.g	*Eutrema salsugineum*	scaffold_23	1571662	1572618
Thhalv10004427m.g	*Eutrema salsugineum*	scaffold_6	975223	976384
Thhalv10006263m.g	*Eutrema salsugineum*	scaffold_19	1921267	1921944
Thhalv10006291m.g	*Eutrema salsugineum*	scaffold_19	2465919	2466646
Thhalv10006454m.g	*Eutrema salsugineum*	scaffold_19	1222045	1222494
Thhalv10006505m.g	*Eutrema salsugineum*	scaffold_19	2402224	2402946
Thhalv10008551m.g	*Eutrema salsugineum*	scaffold_5	5446091	5447172
Thhalv10008673m.g	*Eutrema salsugineum*	scaffold_5	8072007	8072799

Table S2

Thhalv10009187m.g	*Eutrema salsugineum*	scaffold_5	9622060	9622944
Thhalv10014514m.g	*Eutrema salsugineum*	scaffold_2	11485181	11485955
Thhalv10015866m.g	*Eutrema salsugineum*	scaffold_2	2778729	2779253
Thhalv10016759m.g	*Eutrema salsugineum*	scaffold_10	8114319	8115511
Thhalv10017173m.g	*Eutrema salsugineum*	scaffold_10	11031710	11032653
Thhalv10017206m.g	*Eutrema salsugineum*	scaffold_10	7480202	7480995
Thhalv10017346m.g	*Eutrema salsugineum*	scaffold_10	11092283	11094238
Thhalv10017963m.g	*Eutrema salsugineum*	scaffold_10	11497338	11497898
Thhalv10018916m.g	*Eutrema salsugineum*	scaffold_9	996327	997416
Thhalv10019207m.g	*Eutrema salsugineum*	scaffold_9	272333	273094
Thhalv10019299m.g	*Eutrema salsugineum*	scaffold_9	5846380	5847047
Thhalv10019550m.g	*Eutrema salsugineum*	scaffold_9	946690	947019
Thhalv10021464m.g	*Eutrema salsugineum*	scaffold_13	2839871	2841019
Thhalv10021466m.g	*Eutrema salsugineum*	scaffold_13	4235466	4236424
Thhalv10021568m.g	*Eutrema salsugineum*	scaffold_13	1225140	1226065
Thhalv10022101m.g	*Eutrema salsugineum*	scaffold_13	2977157	2978014
Thhalv10025887m.g	*Eutrema salsugineum*	scaffold_1	580157	581035
Thhalv10026244m.g	*Eutrema salsugineum*	scaffold_1	8563671	8564472
Thhalv10026342m.g	*Eutrema salsugineum*	scaffold_1	12263727	12264485
Thhalv10027223m.g	*Eutrema salsugineum*	scaffold_1	929348	929725
Thhalv10028393m.g	*Eutrema salsugineum*	scaffold_3	3410891	3414723
*Tp57577_TGAC_v2_*	*Trifolium pratense*	Tp57577_TGAC_v2_scaf_1	270	1435
*Tp57577_TGAC_v2_*	*Trifolium pratense*	Tp57577_TGAC_v2_LG2	14195686	14197972
*Tp57577_TGAC_v2_*	*Trifolium pratense*	Tp57577_TGAC_v2_LG3	2128112	2131043
*Tp57577_TGAC_v2_*	*Trifolium pratense*	Tp57577_TGAC_v2_LG3	10821203	10823031
*Tp57577_TGAC_v2_*	*Trifolium pratense*	Tp57577_TGAC_v2_scaf_4	183806	185630
*Tp57577_TGAC_v2_*	*Trifolium pratense*	Tp57577_TGAC_v2_scaf_5	65636	66688
*Tp57577_TGAC_v2_*	*Trifolium pratense*	Tp57577_TGAC_v2_LG7	10849687	10850315
*Tp57577_TGAC_v2_*	*Trifolium pratense*	Tp57577_TGAC_v2_scaf_2	186626	194135
*Tp57577_TGAC_v2_*	*Trifolium pratense*	Tp57577_TGAC_v2_LG2	16426220	16427220
*Tp57577_TGAC_v2_*	*Trifolium pratense*	Tp57577_TGAC_v2_scaf_2	56246	57135
*Tp57577_TGAC_v2_*	*Trifolium pratense*	Tp57577_TGAC_v2_scaf_3	210767	213564
*Tp57577_TGAC_v2_*	*Trifolium pratense*	Tp57577_TGAC_v2_LG2	8923799	8924698
*Tp57577_TGAC_v2_*	*Trifolium pratense*	Tp57577_TGAC_v2_LG2	10201325	10202519
*Tp57577_TGAC_v2_*	*Trifolium pratense*	Tp57577_TGAC_v2_scaf_3	100576	101057
*Tp57577_TGAC_v2_*	*Trifolium pratense*	Tp57577_TGAC_v2_LG3	3936684	3937244
*Tp57577_TGAC_v2_*	*Trifolium pratense*	Tp57577_TGAC_v2_LG7	21940000	21940694
*Tp57577_TGAC_v2_*	*Trifolium pratense*	Tp57577_TGAC_v2_LG5	9844690	9845387
*Tp57577_TGAC_v2_*	*Trifolium pratense*	Tp57577_TGAC_v2_LG5	10657414	10658162
*Tp57577_TGAC_v2_*	*Trifolium pratense*	Tp57577_TGAC_v2_LG5	10649334	10650182
*Tp57577_TGAC_v2_*	*Trifolium pratense*	Tp57577_TGAC_v2_LG5	10639474	10648000
*Tp57577_TGAC_v2_*	*Trifolium pratense*	Tp57577_TGAC_v2_scaf_1	5274	5925
*Tp57577_TGAC_v2_*	*Trifolium pratense*	Tp57577_TGAC_v2_scaf_1	253976	254581
*Tp57577_TGAC_v2_*	*Trifolium pratense*	Tp57577_TGAC_v2_LG3	3359460	3360648
*Tp57577_TGAC_v2_*	*Trifolium pratense*	Tp57577_TGAC_v2_scaf_2	1337	1865
*Tp57577_TGAC_v2_*	*Trifolium pratense*	Tp57577_TGAC_v2_LG3	9854332	9855059
*Tp57577_TGAC_v2_*	*Trifolium pratense*	Tp57577_TGAC_v2_scaf_7	31480	32965
*Tp57577_TGAC_v2_*	*Trifolium pratense*	Tp57577_TGAC_v2_LG1	16222138	16231206
*Tp57577_TGAC_v2_*	*Trifolium pratense*	Tp57577_TGAC_v2_LG4	24196044	24197227
*Tp57577_TGAC_v2_*	*Trifolium pratense*	Tp57577_TGAC_v2_scaf_1	20425	21755
*Tp57577_TGAC_v2_*	*Trifolium pratense*	Tp57577_TGAC_v2_LG3	5918713	5919659
*Tp57577_TGAC_v2_*	*Trifolium pratense*	Tp57577_TGAC_v2_LG7	27206325	27207651
*Tp57577_TGAC_v2_*	*Trifolium pratense*	Tp57577_TGAC_v2_scaf_2	238573	239475
*Tp57577_TGAC_v2_*	*Trifolium pratense*	Tp57577_TGAC_v2_scaf_3	1	1385
*Tp57577_TGAC_v2_*	*Trifolium pratense*	Tp57577_TGAC_v2_scaf_5	57376	58307
Traes_1AL_AEBCF3	*Triticum aestivum_v2.2*	ta_iwgsc_1al_v2_3955706	7302	7544
Traes_1AL_AEBCF3	*Triticum aestivum_v2.2*	ta_iwgsc_1al_v2_3955706	7287	7549
Traes_1BL_17A04D6	*Triticum aestivum_v2.2*	ta_iwgsc_1bl_v1_3835690	8290	8529

Table S2

Traes_1DL_BD5E22[illegible]	*Triticum aestivum_v2.2*	ta_iwgsc_1dl_v1_2259655	17370	18084
Traes_2AL_5AE0780	*Triticum aestivum_v2.2*	ta_iwgsc_2al_v1_6425742	3411	4437
Traes_2BL_08C80B0	*Triticum aestivum_v2.2*	ta_iwgsc_2bl_v1_7988902	13007	13743
Traes_2BL_26A39B2[illegible]	*Triticum aestivum_v2.2*	ta_iwgsc_2bl_v1_7988389	10073	10748
Traes_2BL_666C4AF	*Triticum aestivum_v2.2*	ta_iwgsc_2bl_v1_7973968	6630	7269
Traes_2BL_666C4AF	*Triticum aestivum_v2.2*	ta_iwgsc_2bl_v1_7973968	6346	7472
Traes_2BL_A115DF[illegible]	*Triticum aestivum_v2.2*	ta_iwgsc_2bl_v1_8076324	23472	25126
Traes_3AS_22575A3[illegible]	*Triticum aestivum_v2.2*	ta_iwgsc_3as_v1_507703	16	216
Traes_3B_25B6CEF[illegible]	*Triticum aestivum_v2.2*	ta_iwgsc_3b_v1_10420942	1002	1498
Traes_3B_A958F1F7	*Triticum aestivum_v2.2*	ta_iwgsc_3b_v1_10422470	5163	5728
Traes_3B_A958F1F7	*Triticum aestivum_v2.2*	ta_iwgsc_3b_v1_10422470	4995	6420
Traes_3B_A958F1F7	*Triticum aestivum_v2.2*	ta_iwgsc_3b_v1_10422470	5056	5640
Traes_4AS_1A74C1A	*Triticum aestivum_v2.2*	ta_iwgsc_4as_v2_5941526	1981	2723
Traes_4AS_3AD486E	*Triticum aestivum_v2.2*	ta_iwgsc_4as_v2_5901944	764	1213
Traes_4AS_3AD486E	*Triticum aestivum_v2.2*	ta_iwgsc_4as_v2_5901944	47	1484
Traes_4AS_85A2DD[illegible]	*Triticum aestivum_v2.2*	ta_iwgsc_4as_v2_5964577	421	1408
Traes_4BL_9E0B4C[illegible]	*Triticum aestivum_v2.2*	ta_iwgsc_4bl_v1_7015109	1	356
Traes_4BL_9E0B4C[illegible]	*Triticum aestivum_v2.2*	ta_iwgsc_4bl_v1_7015109	1	716
Traes_4BL_E4F29BE	*Triticum aestivum_v2.2*	ta_iwgsc_4bl_v1_7014673	8142	9079
Traes_4DL_788BB68	*Triticum aestivum_v2.2*	ta_iwgsc_4dl_v3_14423362	3757	4922
Traes_4DL_EBB92E[illegible]	*Triticum aestivum_v2.2*	ta_iwgsc_4dl_v3_14376027	1826	2345
Traes_4DL_FD481A[illegible]	*Triticum aestivum_v2.2*	ta_iwgsc_4dl_v3_14109166	4	445
Traes_4DL_FD481A[illegible]	*Triticum aestivum_v2.2*	ta_iwgsc_4dl_v3_14109166	1	385
Traes_5BL_C205AA[illegible]	*Triticum aestivum_v2.2*	ta_iwgsc_5bl_v1_10828952	10473	12676
Traes_5BL_C205AA[illegible]	*Triticum aestivum_v2.2*	ta_iwgsc_5bl_v1_10828952	10299	12432
Traes_5BL_D900FB[illegible]	*Triticum aestivum_v2.2*	ta_iwgsc_5bl_v1_1332183	840	1283
Traes_5BL_D900FB[illegible]	*Triticum aestivum_v2.2*	ta_iwgsc_5bl_v1_1332183	501	1400
Traes_5BS_29454AF[illegible]	*Triticum aestivum_v2.2*	ta_iwgsc_5bs_v1_2262073	286	1023
Traes_5BS_29454AF[illegible]	*Triticum aestivum_v2.2*	ta_iwgsc_5bs_v1_2262073	12	1413
Traes_5DL_E80D1C[illegible]	*Triticum aestivum_v2.2*	ta_iwgsc_5dl_v1_4516522	1779	2222
Traes_5DL_E80D1C[illegible]	*Triticum aestivum_v2.2*	ta_iwgsc_5dl_v1_4516522	1399	2415
Traes_5DS_D3E79E[illegible]	*Triticum aestivum_v2.2*	ta_iwgsc_5ds_v1_2278349	716	2154
Traes_7DL_C276851	*Triticum aestivum_v2.2*	ta_iwgsc_7dl_v1_3330926	7971	8585
Traes_7DL_C276851	*Triticum aestivum_v2.2*	ta_iwgsc_7dl_v1_3330926	7666	8768
Zosma114g00230	*Zostera marina*	scaffold_114	110320	110920
Zosma140g00160	*Zostera marina*	scaffold_140	171593	172517
Zosma146g00250	*Zostera marina*	scaffold_146	131238	132511
Zosma17g01270	*Zostera marina*	scaffold_17	988766	989227
Zosma181g00160	*Zostera marina*	scaffold_181	132211	132985
Zosma201g00200	*Zostera marina*	scaffold_201	188019	190416
Zosma23g01310	*Zostera marina*	scaffold_23	925709	926818
Zosma250g00040	*Zostera marina*	scaffold_250	89439	90047
Zosma25g00790	*Zostera marina*	scaffold_25	524299	525761
Zosma317g00060	*Zostera marina*	scaffold_317	42005	42646
Zosma319g00030	*Zostera marina*	scaffold_319	52926	53935
Zosma320g00020	*Zostera marina*	scaffold_320	72373	72880
Zosma388g00080	*Zostera marina*	scaffold_388	22359	22799
Zosma4g01760	*Zostera marina*	scaffold_4	1297227	1298411
Zosma56g01070	*Zostera marina*	scaffold_56	421220	423571
Zosma56g01590	*Zostera marina*	scaffold_56	691471	692085
Zosma69g00530	*Zostera marina*	scaffold_69	329596	330268
Zosma6g00080	*Zostera marina*	scaffold_6	148783	149755
Zosma88g00310	*Zostera marina*	scaffold_88	307297	308576

* No *VQs* were identified in 6 alga genomes.

* *VQs* from *Arabidopsis thaliana, Brassica rapa, Glycine max, Vitis vinifera, Zea mays and Oryza sativa* have been previously identified and were excluded.
