# Supplementary material for: Valine-glutamine (VQ) motif coding genes are ancient and non-plant-specific with comprehensive expression regulation by various biotic and abiotic stresses
Source: BMC Genomics. 2018 May 9;19:342. doi: 10.1186/s12864-018-4733-7 (PMC5941492; doi:10.1186/s12864-018-4733-7)
Supplement: Supplementary file 3 — Table S3. Nematode and fungus species used for the genome-wide identification of VQs. (PDF 107 kb) [file 12864_2018_4733_MOESM3_ESM.pdf]

Additional file 3: Table S3. Nematode and fungus species used for the genome-wde identification of VQs

Classification	Species	Host type	No of VQs
Nematodes	*Caenorhabditis angaria*	Free-living	0
	Caenorhabditis brenneri	Free-living	1
	Caenorhabditis briggsae	Free-living	1
	Caenorhabditis elegans	Free-living	1
	Caenorhabditis japonica	Free-living	1
	Caenorhabditis remanei	Free-living	1
	Caenorhabditis sp11	Free-living	0
	Caenorhabditis sp5	Free-living	0
	Pristionchus pacificus	Free-living	0
	Zeldia punctata	Free-living	0
	Ancylostoma ceylanicum	Human Parasitic Nematodes	0
	Ancylostoma duodenale	Human Parasitic Nematodes	0
	Angiostrongylus cantonensis	Human Parasitic Nematodes	0
	Brugia malayi	Human Parasitic Nematodes	0
	Dracunculus medinensis	Human Parasitic Nematodes	0
	Loa loa	Human Parasitic Nematodes	0
	Necator americanus	Human Parasitic Nematodes	0
	Onchocerca volvulus	Human Parasitic Nematodes	0
	Trichinella spiralis	Human Parasitic Nematodes	0
	Trichuris trichiura	Human Parasitic Nematodes	0
	Acanthocheilonema viteae	Animal Parasitic Nematodes	0
	Ancylostoma caninum	Animal Parasitic Nematodes	0
	Anisakis simplex	Animal Parasitic Nematodes	0
	Dictyocaulus viviparus	Animal Parasitic Nematodes	0
	Haemonchus contortus	Animal Parasitic Nematodes	1
	Oesophagostomum dentatum	Animal Parasitic Nematodes	0
	Onchocerca flexuosa	Animal Parasitic Nematodes	0
	Strongyloides ratti	Animal Parasitic Nematodes	0
	Teladorsagia circumcincta	Animal Parasitic Nematodes	0
	Toxocara canis	Animal Parasitic Nematodes	0
	Xenorhabdus nematophila	Animal Parasitic Nematodes	0
	Bursaphelenchus xylophilus	Plant Parasitic Nematodes	0
	Ditylenchus africanus	Plant Parasitic Nematodes	0
	Globodera pallida	Plant Parasitic Nematodes	2
	Globodera rostochiensis	Plant Parasitic Nematodes	2
	Meloidogyne arenaria	Plant Parasitic Nematodes	0
	Meloidogyne floridensis	Plant Parasitic Nematodes	2
	Meloidogyne hapla	Plant Parasitic Nematodes	1
	Meloidogyne incognita	Plant Parasitic Nematodes	1
	Pratylenchus penetrans	Plant Parasitic Nematodes	0
	Xiphinema index	Plant Parasitic Nematodes	0
	Heterorhabditis bacteriophora	Entomopathogenic Nematodes	0
	Steinernema carpocapsae	Entomopathogenic Nematodes	0
Fungi	*Absidia glauca*	-	0
	Acidomyces richmondensis	-	0
	Acremonium chrysogenum	-	0
	Agaricus bisporus	-	0
	Allomyces macrogynus	-	0
	Alternaria alternata	-	0
	Amanita muscaria	-	1
	Anthracocystis flocculosa	-	0
	Arthrobotrys oligospora	-	0
	Arthroderma otae	-	0
	Aschersonia aleyrodis	-	0
	Ascochyta rabiei	-	0
	Ascoidea rubescens	-	0
	Ashbya gossypii	-	0

Aspergillus aculeatus	-	0
Aspergillus bombycis	-	1
Aspergillus flavus	-	1
Aspergillus nomius	-	1
Aspergillus oryzae	-	1
Aspergillus parasiticus	-	1
Aureobasidium melanogenum	-	0
Babjeviella inositovora	-	0
Batrachochytrium salamandrivorans	-	0
Baudoinia panamericana	-	0
Blastomyces dermatitidis	-	0
Blumeria graminis	-	0
Botryobasidium botryosum	-	0
Botrytis cinerea	-	0
Brettanomyces bruxellensis	-	0
Byssochlamys spectabilis	-	0
Calocera cornea	-	0
Candida albicans	-	1
Ceraceosorus bombacis	-	0
Ceratocystis platani	-	0
Chaetomium globosum	-	1
Choanephora cucurbitarum	-	0
Claviceps purpurea	-	0
Clavispora lusitaniae	-	0
Coccidioides immitis	-	0
Colletotrichum chlorophyti	-	0
Conidiobolus coronatus	-	0
Coniochaeta ligniaria	-	0
Coniophora puteana	-	0
Coprinopsis cinerea	-	0
Cordyceps brongniartii	-	0
Cryptococcus depauperatus	-	0
Cutaneotrichosporon oleaginosus	-	0
Cyberlindnera fabianii	-	0
Cylindrobasidium torrendii	-	1
Dacryopinax primogenitus	-	0
Dactylellina haptotyla	-	0
Daedalea quercina	-	0
Diaporthe ampelina	-	0
Dichomitus squalens	-	0
Diplodia corticola	-	0
Dothistroma septosporum	-	1
Drechmeria coniospora	-	0
Edhazardia aedis	-	0
Emergomyces pasteuriana	-	0
Emmonsia crescens	-	0
Encephalitozoon cuniculi	-	0
Endocarpon pusillum	-	0
Enterocytozoon bieneusi	-	0
Enterospora canceri	-	0
Eremothecium cymbalariae	-	0
Erysiphe necator	-	0
Escovopsis weberi	-	0
Eutypa lata	-	0
Exidia glandulosa	-	0
Exophiala aquamarina	-	0
Fibroporia radiculosa	-	0
Fistulina hepatica	-	0
Fomitiporia mediterranea	-	0

Fomitopsis pinicola	-	0
Fonsecaea erecta	-	0
Fusarium fujikuroi	-	1
Fusarium mangiferae	-	1
Fusarium oxysporum	-	1
Fusarium proliferatum	-	1
Fusarium verticillioides	-	0
Gaeumannomyces graminis	-	0
Galerina marginata	-	0
Gelatoporia subvermispora	-	0
Geotrichum candidum	-	0
Gibberella fujikuroi	-	2
Glarea lozoyensis	-	0
Gloeophyllum trabeum	-	1
Gonapodya prolifera	-	0
Grifola frondosa	-	0
Grosmannia clavigera	-	0
Gymnopus luxurians	-	0
Hanseniaspora guilliermondii	-	0
Hebeloma cylindrosporum	-	1
Heterobasidion irregulare	-	0
Hirsutella minnesotensis	-	0
Histoplasma capsulatum	-	0
Hydnomerulius pinastri	-	0
Hypholoma sublateritium	-	0
Hypsizygus marmoreus	-	2
Isaria fumosorosea	-	0
Jaapia argillacea	-	0
Kalmanozyma brasiliensis	-	0
Kazachstania africana	-	0
Kluyveromyces lactis	-	0
Kluyveromyces marxianus	-	2
Komagataella pastoris	-	0
Kwoniella pini	-	0
Laccaria bicolor	-	0
Lachancea dasiensis	-	0
Laetiporus sulphureus	-	0
Lentinula edodes	-	1
Leptosphaeria maculans	-	0
Lichtheimia ramosa	-	0
Lipomyces starkeyi	-	0
Lodderomyces elongisporus	-	0
Macrophomina phaseolina	-	0
Madurella mycetomatis	-	0
Magnaporthe poae	-	0
Malassezia sympodialis	-	0
Marssonina brunnea	-	0
Melampsora laricipopulina	-	0
Metarhizium robertsii	-	0
Microbotryum violaceum	-	0
Microdochium bolleyi	-	0
Millerozyma farinosa	-	0
Mitosporidium daphniae	-	0
Mixia osmundae	-	1
Moesziomyces aphidis	-	0
Moniliophthora roreri	-	0
Mortierella verticillata	-	0
Mucor circinelloides	-	0
Mycosphaerella eumusae	-	0

Nadsonia fulvescens	-	0
Nannizzia gypsea	-	0
Naumovozyma dairenensis	-	0
Neolecta irregularis	-	0
Neolentinus lepideus	-	0
Neonectria ditissima	-	0
Neosartorya fischeri	-	0
Neurospora tetrasperma	-	0
Nosema ceranae	-	0
Ogataea polymorpha	-	0
Oidiodendron maius	-	0
Ophiocordyceps unilateralis	-	0
Ordospora colligata	-	0
Pachysolen tannophilus	-	0
Paraphaeosphaeria sporulosa	-	0
Parasitella parasitica	-	0
Paxillus involutus	-	0
Paxillus rubicundulus	-	2
Penicilliopsis zonata	-	0
Penicillium vulpinum	-	0
Phaeoacremonium minimum	-	0
Phaeomoniella chlamydospora	-	0
Phaeosphaeria nodorum	-	0
Phanerochaete carnosa	-	0
Phialocephala subalpina	-	0
Phialophora americana	-	0
Phlebiopsis gigantea	-	0
Phycomyces blakesleeanus	-	0
Pichia membranifaciens	-	0
Piloderma croceum	-	0
Pisolithus tinctorius	-	0
Pleurotus ostreatus	-	0
Plicaturopsis crispa	-	0
Pneumocystis murina	-	0
Pochonia chlamydosporia	-	0
Podospora anserina	-	0
Postia placenta	-	0
Pseudocercospora musae	-	0
Pseudogymnoascus verrucosus	-	0
Pseudoloma neurophilia	-	0
Pseudozyma hubeiensis	-	0
Puccinia triticina	-	0
Punctularia strigosozonata	-	0
Purpureocillium lilacinum	-	0
Pyrenophora triticirepentis	-	0
Pyronema omphalodes	-	1
Rachicladosporium antarcticum	-	0
Rasamsonia emersonii	-	0
Rhinocladiella mackenziei	-	0
Rhizoctonia solani	-	1
Rhizopogon vinicolor	-	0
Rhizopus microsporus	-	0
Rhynchosporium secalis	-	0
Rozella allomycis	-	0
Saccharomyces cerevisiae	-	0
Saitoella complicata	-	0
Scedosporium apiospermum	-	0
Schizophyllum commune	-	0
Schizopora paradoxa	-	1

Schizosaccharomyces pombe	-	0
Scleroderma citrinum	-	0
Sclerotinia sclerotiorum	-	0
Serendipita vermifera	-	0
Serpula lacrymans	-	0
Setosphaeria turcica	-	0
Sistotremastrum suecicum	-	0
Sordaria macrospora	-	0
Spathaspora passalidarum	-	0
Sphaerobolus stellatus	-	0
Sphaerulina musiva	-	0
Spizellomyces punctatus	-	0
Sporidiobolus salmonicolor	-	0
Sporisorium scitamineum	-	0
Sporothrix schenckii	-	0
Spraguea lophii	-	0
Stachybotrys chlorohalonata	-	0
Stemphylium lycopersici	-	0
Stereum hirsutum	-	0
Sugiyamaella lignohabitans	-	0
Suillus luteus	-	0
Talaromyces stipitatus	-	0
Tetrapisispora phaffii	-	0
Thermothelomyces thermophila	-	0
Thielavia terrestris	-	0
Thielaviopsis punctulata	-	0
Tilletia controversa	-	0
Tilletia indica	-	1
Tilletia walkeri	-	1
Tilletiaria anomala	-	0
Tolypocladium ophioglossoides	-	0
Torrubiella hemipterigena	-	0
Tortispora caseinolytica	-	0
Torulaspora delbrueckii	-	0
Trachipleistophora hominis	-	0
Trametes versicolor	-	0
Tremella mesenterica	-	0
Trichoderma guizhouense	-	1
Trichoderma reesei	-	0
Trichophyton violaceum	-	0
Trichosporon asahii	-	0
Tuber melanosporum	-	0
Tulasnella calospora	-	0
Umbilicaria pustulata	-	2
Uncinocarpus reesii	-	0
Ustilaginoidea virens	-	0
Ustilago maydis	-	0
Valsa mali	-	0
Vanderwaltozyma polyspora	-	0
Vavraia culicis	-	0
Vittaforma corneae	-	0
Wallemia mellicola	-	0
Wickerhamomyces ciferrii	-	0
Xanthophyllomyces dendrorhous	-	0
Xylona heveae	-	0
Yarrowia lipolytica	-	0
Zygosaccharomyces rouxii	-	0
Zymoseptoria tritici	-	0
