# Supplementary material for: Valine-glutamine (VQ) motif coding genes are ancient and non-plant-specific with comprehensive expression regulation by various biotic and abiotic stresses
Source: BMC Genomics. 2018 May 9;19:342. doi: 10.1186/s12864-018-4733-7 (PMC5941492; doi:10.1186/s12864-018-4733-7)
Supplement: Supplementary file 4 — Table S4. Identification of VQs in some of bacterial species. (PDF 13 kb) [file 12864_2018_4733_MOESM4_ESM.pdf]

Additional file 4: Table S4. Identification of *VQs* in some of bacterial species

Species name	**Accession No**	VQ motif from	VQ motif to	Motif Seq
Aeromonas diversa	G114_03853	269	286	ASVAEFKALVDRLTGIYQ
Enterobacter sp. BWH64	SK50_02740	115	131	YQLAQSETFRQVVQQLT
Lactobacillus camelliae	FC75_GL000025	35	49	TSAEDFKVMVQALTG
Lactobacillus manihotivorans	FD01_GL002336	35	49	TDAEDFKVLVQALTG
Lactobacillus paracasei	Lpp126_02327	39	50	DFKTLVQALTGM
Leptospira terpstrae	LEP1GSC203_2036	325	338	KEFMELVQTITGFQ
Phaeospirillum molischianum	PHAMO_230004	242	260	PRFYVTSAHRDFKSMVQRL
Weissella oryzae	WOSG25_040030	135	148	PKQFMDEVQQLTGK
