# Supplementary figures and images for: Valine-glutamine (VQ) motif coding genes are ancient and non-plant-specific with comprehensive expression regulation by various biotic and abiotic stresses
Source: BMC Genomics. 2018 May 9;19:342. doi: 10.1186/s12864-018-4733-7 (PMC5941492; doi:10.1186/s12864-018-4733-7)

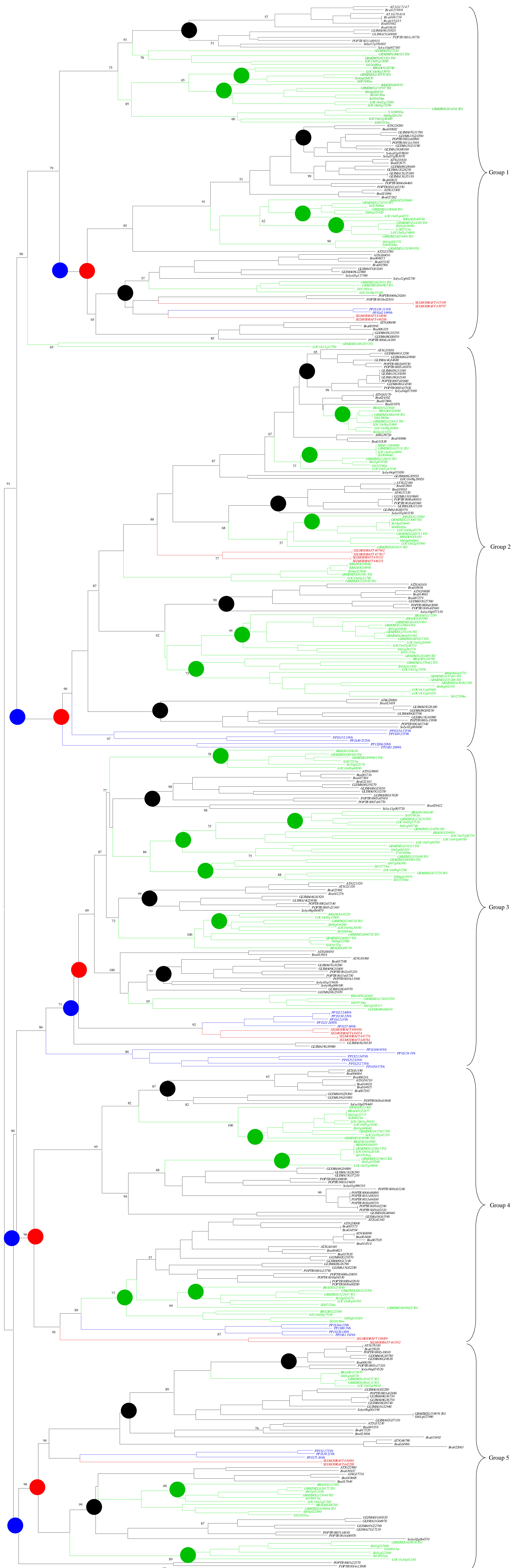

Additional file 5 Figure S1. The enlarged phylogenetic tree of Figure 2a.

Supplement: Supplementary file 5 — Figure S1. The enlarged phylogenetic tree of Fig. 2a. (PDF 717 kb) [file 12864_2018_4733_MOESM5_ESM.pdf]
