# Supplementary material for: Valine-glutamine (VQ) motif coding genes are ancient and non-plant-specific with comprehensive expression regulation by various biotic and abiotic stresses
Source: BMC Genomics. 2018 May 9;19:342. doi: 10.1186/s12864-018-4733-7 (PMC5941492; doi:10.1186/s12864-018-4733-7)
Supplement: Supplementary file 6 — Figure S2. Syntenic analysis of orthologous VQs among ten species of the Oryza genus. (PDF 218 kb) [file 12864_2018_4733_MOESM6_ESM.pdf]

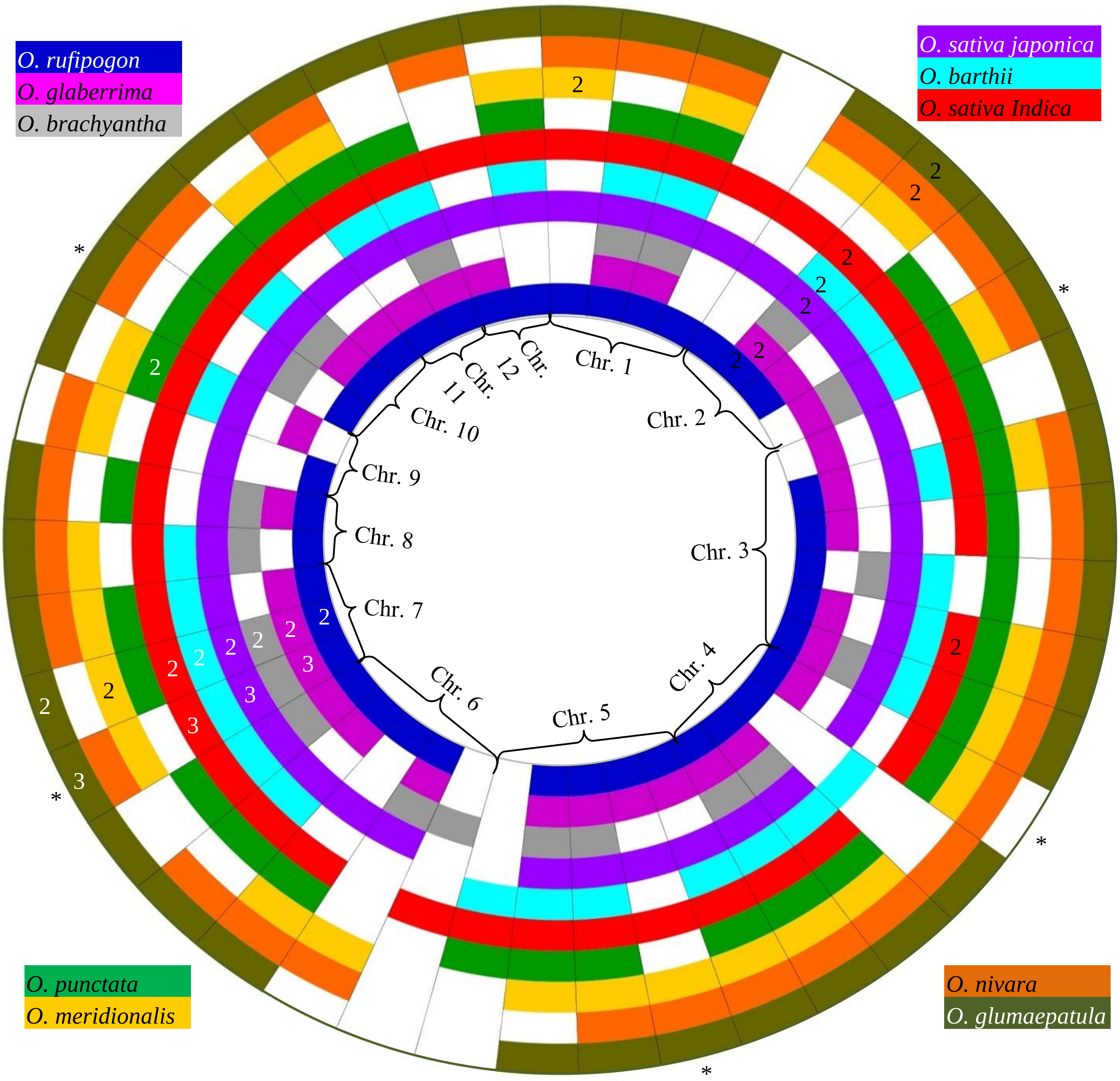

Additional file 6: **Figure S2**. Syntenic analysis of orthologous *VQs* among ten species of the *Oryza* genus. The coordinates were mapped using the GenomeRing program (Herbig et al. 2012. *Bioinformatics* **28,** i7-i15). The stars indicated that these loci were undergone positive selection with *Ka/Ks* value significantly larger than 1. The numbers indicated copies of tandemly duplicated genes in these loci among different species. Locus names and coordinates were listed in Table S3.
