# Supplementary material for: Valine-glutamine (VQ) motif coding genes are ancient and non-plant-specific with comprehensive expression regulation by various biotic and abiotic stresses
Source: BMC Genomics. 2018 May 9;19:342. doi: 10.1186/s12864-018-4733-7 (PMC5941492; doi:10.1186/s12864-018-4733-7)
Supplement: Supplementary file 7 — Table S5. Identification of gene conversion events in the VQ motif family. (PDF 15 kb) [file 12864_2018_4733_MOESM7_ESM.pdf]

Additional file 7: Table S5. Identification of gene conversion events in the VQ motif family

Species	Gene conversion pairs	P-value	Begin	End	Length
Zea mays	*GRMZM2G023921 vs GRMZM2G055404*	0.0065	12	36	25
	GRMZM2G064903 vs GRMZM2G055404	0.0121	12	36	25
Glycine max	*GLYMA13G26290 vs GLYMA14G34680*	0.023	40	54	15
	GLYMA15G37230 vs GLYMA14G34680	0.023	40	54	15
Brassica rapa	*Bra022675 vs Bra008473*	0.0049	19	33	15
	Bra035182 vs Bra037588	0.0235	53	72	20
	Bra039565 vs Bra007505	0.0438	23	33	11
	Bra003032 vs Bra008473	0.0447	19	33	15
Brachypodium distachyon	*BRADI1G35890 vs BRADI1G32060*	0.0332	31	49	19
	BRADI4G00780 vs BRADI1G35890	0.0477	49	71	23
Selaginella moellendorffii	*SELMODRAFT_415509 vs SELMODRAFT_438797*	0	106	209	104
	SELMODRAFT_415509 vs SELMODRAFT_438797	0	24	104	81
	SELMODRAFT_410083 vs SELMODRAFT_442289	0.0442	46	194	149
