# Supplementary material for: Valine-glutamine (VQ) motif coding genes are ancient and non-plant-specific with comprehensive expression regulation by various biotic and abiotic stresses
Source: BMC Genomics. 2018 May 9;19:342. doi: 10.1186/s12864-018-4733-7 (PMC5941492; doi:10.1186/s12864-018-4733-7)
Supplement: Supplementary file 8 — Table S6. The VQ gene family members and their coordinates among 10 species from the Oryza genus. (PDF 49 kb) [file 12864_2018_4733_MOESM8_ESM.pdf]

Additional file 8: Table S6. The VQ gene family members and their coordinates among 10 species from the *Oryza* genus

Chromosome	Coordinates	*Oryza rufipogon*	*Oryza glaberrima*	*Oryza brachyantha*	*Oryza sativa japonica*	*Oryza barthii*	*Oryza sativa Indica*	*Oryza punctata*	*Oryza meridionalis*	*Oryza nivara*	*Oryza glumaepatula*
Chr1	1	*ORUFI01G12270*			*LOC_Os01g17050*		*BGIOSGA001894*		*OMERI01G11190/OMERI01G11200*	*ONIVA01G13640*	*OGLUM01G12770*
	2	*ORUFI01G27780*	*ORGLA01G0202500*	*OB01G34080*	*LOC_Os01g46440*	*OBART01G24900*	*BGIOSGA001106*	*OPUNC01G24880*		*ONIVA01G28180*	*OGLUM01G28720*
	3	*ORUFI01G33870*	*ORGLA01G0251600*	*OB01G39640*	*LOC_Os01g54400*	*OBART01G30720*	*BGIOSGA000834*	*OPUNC01G15710*	*OMERI01G27840*	*ONIVA01G35110*	*OGLUM01G34880*
	4	*ORUFI01G37830*			*LOC_Os01g59410*		*BGIOSGA004652*				
Chr2	5	*ORUFI02G05660*			*LOC_Os02g07690*		*BGIOSGA007637*		*OMERI02G06350*	*ONIVA02G05890*	*OGLUM02G05440*
	6	*ORUFI02G10700*	*ORGLA02G0097300*	*OB02G19620*	*LOC_Os02g15280*	*OBART02G10760*	*BGIOSGA006777*		*OMERI02G11720*	*ONIVA02G11970*	*OGLUM02G10710*
	6	*ORUFI02G10710*	*ORGLA02G0097400*		*LOC_Os02g15290*	*OBART02G10770*	*BGIOSGA006776*			*ONIVA02G11980*	*OGLUM02G10720*
	7	*ORUFI02G20620*	*ORGLA02G0167700*		*LOC_Os02g33600*	*OBART02G19570*	*BGIOSGA008380*	*OPUNC02G17470*		*ONIVA03G19910*	*OGLUM02G19860*
	8		*ORGLA02G0283900**	*OB02G39830*	*LOC_Os02g51740**	*OBART02G32830**	*BGIOSGA009072**	*OPUNC02G30310*	*OMERI02G31460*	*ONIVA02G35000**	*OGLUM02G33440*
Chr3	9		*ORGLA03G0063500*		*LOC_Os03g09045*		*BGIOSGA012021*	*OPUNC03G06290*			*OGLUM03G06610*
	10	*ORUFI03G15910*	*ORGLA03G0146500*		*LOC_Os03g20330*	*OBART03G15300*	*BGIOSGA012511*	*OPUNC03G14780*	*OMERI03G13940*	*ONIVA03G16300*	*OGLUM03G15480*
	11	*ORUFI03G16000*	*ORGLA03G0147500*		*LOC_Os03g20440*		*BGIOSGA010844*	*OPUNC03G14930*		*ONIVA03G16390*	*OGLUM03G15570*
	12	*ORUFI03G20440*		*OB03G29000*	*LOC_Os03g26990*	*OBART03G19810*		*OPUNC03G18680*		*ONIVA03G21480*	*OGLUM03G20410*
	13	*ORUFI03G30520*	*ORGLA03G0272300*		*LOC_Os03g47280*	*OBART03G29370*	*BGIOSGA010063/BGIOSGA010064*	*OPUNC03G26820*	*OMERI03G25610*	*ONIVA03G30590*	*OGLUM03G29590*
	14	*ORUFI03G38330*	*ORGLA03G0337400*	*OB03G43740*	*LOC_Os03g57520*	*OBART03G36840*	*BGIOSGA013698*	*OPUNC03G33830*	*OMERI03G34000*	*ONIVA03G38070*	*OGLUM03G36520*
Chr4	15	*ORUFI04G13420*	*ORGLA04G0092300**		*LOC_Os04g34050*		*BGIOSGA016385**	*OPUNC04G10270*	*OMERI04G10970*	*ONIVA04G10200**	
	16	*ORUFI04G26110*				*OBART04G24390*				*ONIVA04G23580*	*OGLUM04G24340*
	17	*ORUFI04G28930*	*ORGLA04G0235300*	*OB04G34240*	*LOC_Os04g55240*	*OBART04G27290*	*BGIOSGA014233*	*OPUNC04G24760*	*OMERI04G22720*	*ONIVA04G25590*	*OGLUM04G27220*
	18	*ORUFI04G30380*	*ORGLA04G0250000*	*OB04G35710*	*LOC_Os04g57030*	*OBART04G28710*	*BGIOSGA014150*	*OPUNC04G26230*	*OMERI04G24280*	*ONIVA04G27240*	*OGLUM04G28580*
Chr5	19	*ORUFI05G07600*	*ORGLA05G0061400*		*LOC_Os05g12090*	*OBART05G07070*	*BGIOSGA018594*	*OPUNC05G06600*	*OMERI05G06220*	*ONIVA05G07900*	*OGLUM05G07430*
	20	*ORUFI05G16310*	*ORGLA05G0130500*		*LOC_Os05g32460*		*BGIOSGA018119**		*OMERI05G13530*	*ONIVA05G15610**	*OGLUM05G16090*
	21	*ORUFI05G22450*	*ORGLA05G0184000*	*OB05G28690*	*LOC_Os05g41250*	*OBART05G21210*	*BGIOSGA017809*	*OPUNC05G18900*	*OMERI05G19150*	*ONIVA05G21880*	*OGLUM05G22420*
	22	*ORUFI05G24370*	*ORGLA05G0201700*	*OB05G30560*	*LOC_Os05g44270*	*OBART05G22920*	*BGIOSGA020204*	*OPUNC05G20510*	*OMERI05G20710*		*OGLUM05G24270*
	23					*OBART05G26550*	*BGIOSGA020407*	*OPUNC05G24140*			
Chr6	24			*OB06G13460*			*BGIOSGA021871*				
	25	*ORUFI06G18150*	*ORGLA06G0142600*	*OB06G24870*	*LOC_Os06g33970*				*OMERI06G17990*	*ONIVA06G20420*	
	26	*ORUFI06G21740*			*LOC_Os06g40090*		*BGIOSGA020926*	*OPUNC06G18530*	*OMERI06G20940*	*ONIVA06G24100*	*OGLUM06G21380*
	27	*ORUFI06G22660*	*ORGLA06G0176100*		*LOC_Os06g41450*	*OBART06G21100*	*BGIOSGA020881/BGIOSGA020884*	*OPUNC06G19220*		*ONIVA06G25040*	*OGLUM06G22290*
	28	*ORUFI06G25800*	*ORGLA06G0201800*	*OB06G31760*	*LOC_Os06g45570*	*OBART06G24010*	*BGIOSGA023429*	*OPUNC06G21760*			*OGLUM06G25270*
Chr7	29		*ORGLA07G0032700*	*OB07G12890*	*LOC_Os07g06750*	*OBART07G03890**	*BGIOSGA025193*		*OMERI07G02440**	*ONIVA07G02430*	*OGLUM07G03240*
	29		*ORGLA07G0032800*		*LOC_Os07g06760*		*BGIOSGA025194**				*OGLUM07G03250**
	29	*ORUFI07G03590*	*ORGLA07G0032900*		*LOC_Os07g06790*		*BGIOSGA025195*			*ONIVA07G02460*	*OGLUM07G03270*
	30	*ORUFI07G23830*	*ORGLA07G0176400*	*OB07G28250*	*LOC_Os07g43140*	*OBART07G22770*	*BGIOSGA023883*	*OPUNC07G21370*	*OMERI07G19160*		*OGLUM07G22740*
	31	*ORUFI07G27790*	*ORGLA07G0212400*		*LOC_Os07g48710*	*OBART07G26840*	*BGIOSGA026421*	*OPUNC07G25220*	*OMERI07G23450*	*ONIVA07G26580*	*OGLUM07G26830*
	31	*ORUFI07G27860*	*ORGLA07G0213100*		*LOC_Os07g48800*	*OBART07G26920*	*BGIOSGA023688*		*OMERI07G23530*	*ONIVA07G26660*	*OGLUM07G26900*
Chr8	32	*ORUFI08G00260*		*OB08G10290*	*LOC_Os08g01260*	*OBART08G00230*	*BGIOSGA027834*		*OMERI08G00530*	*ONIVA08G00220*	*OGLUM08G00280*
	33	*ORUFI08G16220*	*ORGLA08G0125500*	*OB08G22000*	*LOC_Os08g31660*		*BGIOSGA028654*	*OPUNC08G13420*		*ONIVA08G15230*	*OGLUM08G15090*
Chr9	34	*ORUFI09G08090*			*LOC_Os09g20020*		*BGIOSGA029897*		*OMERI09G06010*	*ONIVA09G06700*	
	35		*ORGLA09G0058000*		*LOC_Os09g20460*	*OBART09G07950*	*BGIOSGA030630*	*OPUNC09G06750/OPUNC09G06760*	*OMERI09G06280*		*OGLUM09G08320*
Chr10	36	*ORUFI10G00100*		*OB10G10130*	*LOC_Os10g01240*		*BGIOSGA032413**	*OPUNC10G00120**		*ONIVA10G00010**	*OGLUM10G00100**
	37	*ORUFI10G20720*	*ORGLA10G0146300*	*OB10G26590*	*OS10g0575401*	*OBART10G19430*	*BGIOSGA031344*	*OPUNC10G17880*		*ONIVA10G22050*	*OGLUM10G19530*
	38	*ORUFI10G20850*	*ORGLA10G0147800*		*LOC_Os10g42650*		*BGIOSGA031335*	*OPUNC10G18050*	*OMERI10G15200*		*OGLUM10G19670*
Chr11	39	*ORUFI11G01790*	*ORGLA11G0015900*		*LOC_Os11g03660*	*OBART11G01850*	*BGIOSGA034725*	*OPUNC11G01750*	*OMERI11G06470*	*ONIVA11G07830*	*OGLUM11G01600*
	40	*ORUFI11G07930*	*ORGLA11G0068400*	*OB11G16060*	*LOC_Os11g12790*	*OBART11G11290*	*BGIOSGA034242*	*OPUNC11G07480*			*OGLUM11G07540*
Ch12	41	*ORUFI12G01880*	*ORGLA12G0014400*		*LOC_Os12g03420*		*BGIOSGA036930*			*ONIVA11G01890*	*OGLUM12G02200*
	42	*ORUFI12G22240*			*OS12g0635400*	*OBART12G19920*	*BGIOSGA035768*	*OPUNC12G18190*	*OMERI12G14750*		*OGLUM12G21660*
