# Supplementary material for: Valine-glutamine (VQ) motif coding genes are ancient and non-plant-specific with comprehensive expression regulation by various biotic and abiotic stresses
Source: BMC Genomics. 2018 May 9;19:342. doi: 10.1186/s12864-018-4733-7 (PMC5941492; doi:10.1186/s12864-018-4733-7)
Supplement: Supplementary file 9 — Figure S3. Expression profiling of rice VQs among various tissues and under abiotic and biotic stresses. (PDF 144 kb) [file 12864_2018_4733_MOESM9_ESM.pdf]

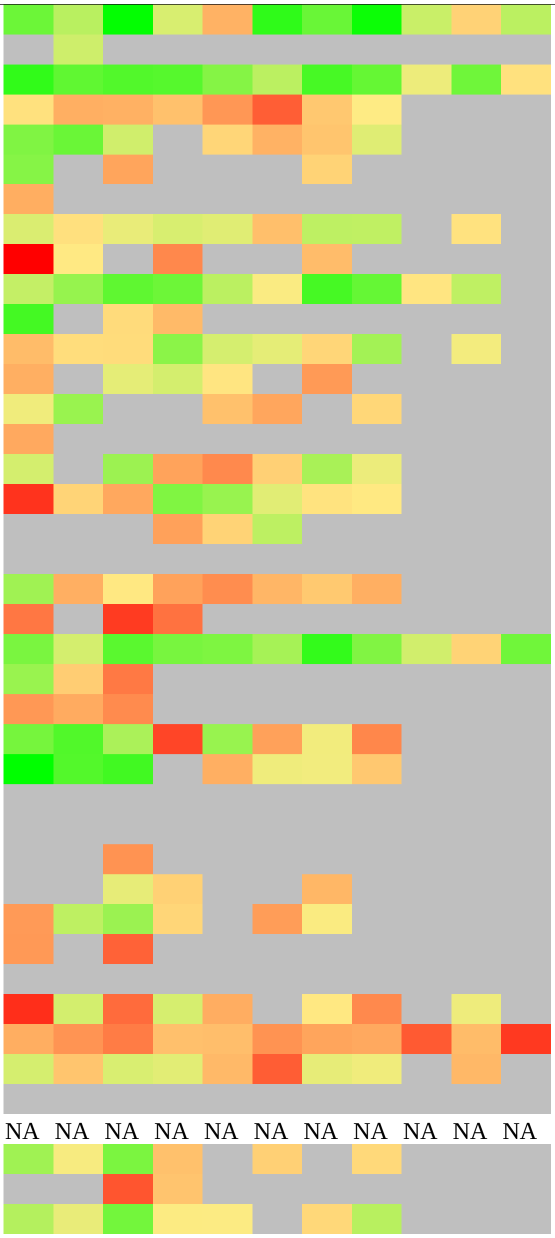

(a)												(b)		(c)			(d)	
VQ genes	Tissues											Hormone		Biotic stresses			Abiotic stresses	
	T1	T2	T3	T4	T5	T6	T7	T8	T9	T10	T11	ABA	JA	*Xoo*	*Xoc*	*Mo*	Drought	Salinity
01g17050												-	0	0	NA	+	-	0
01g46440												NA	NA	+	NA	NA	+	NA
01g54400												0	-	+	NA	0	0	0
01g59410												NA	NA	0	NA	-	+	NA
02g07690												+	+	0	+-	+-	+	+
02g15280												+	0	0	NA	NA	+	+
02g15290												+	+	+	NA	+	0	+
02g33600												0	+	+	+-	+-	+	NA
02g51740												0	0	+	NA	+	0	0
03g09045												NA	NA	NA	+-	+-	NA	NA
03g20330												+	+	+	NA	+	0	+
03g20440												-	0	+	-	+-	0	0
03g26990												-	+	+	NA	+	0	0
03g47280												+	+	+	+-	+-	+	+
03g57520												0	0	+	+	+-	+	0
04g34050												0	0	+	+	+-	0	0
04g55240												0	-	+	NA	0	+	+
04g57030												0	0	NA	NA	NA	+	0
05g12090												0	0	+	NA	NA	+	+
05g32460												0	-	+	NA	0	NA	0
05g41250												-	-	+	NA	-	+	0
05g44270												0	+	0	NA	+	0	0
06g33970												0	+	+	+-	+-	NA	+
06g40090												NA	NA	NA	NA	NA	NA	+
06g41450												-	-	0	-	+	-	-
06g45570												-	+	+	NA	+	+	+
07g06750												-	0	+	NA	NA	+	+
07g06760												0	-	+	NA	0	+	0
07g06790												0	0	+	NA	NA	0	0
07g43140												0	0	+	NA	NA	0	0
07g48710												+	+	+	+-	+-	+	+
07g48800												-	+	+	+-	+-	+	0
08g01260												0	0	+	+-	+-	+	0
08g31660												0	+	+	NA	-	0	0
09g20020												-	0	0	+-	+-	+	0
09g20460												NA	NA	0	NA	+	-	NA
10g01240												-	0	+	NA	NA	+	0
*10g0575401**	NA	NA	NA	NA	NA	NA	NA	NA	NA	NA	NA	NA	NA	0	NA	NA	NA	NA
11g03660												NA	NA	0	NA	+	+	NA
11g12790												0	0	+	NA	NA	+	0
12g03420												0	-	+	NA	-	+	NA
*12g0635400**	NA	NA	NA	NA	NA	NA	NA	NA	NA	NA	NA	+	-	+	NA	+-	NA	0
Percentage (%)												40.5	47.6	78.6	31.0	61.9	59.5	31.0

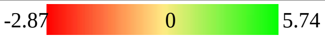

Additional file 9: Figure S3. Expression profiling of rice *VQs* among various tissues and under abiotic and biotic stresses. (a) Expression patterns of *VQs* among different tissues. The accession numbers of RNA-Seq data were listed in the Methods. The expression abundance was log2-transferred and was then used for heatmap drawing. A total of 5 VQs showed no expression and were labelled with red color. The *VQs* highlighted with blue color showed vegetative stage specific expression pattern. *VQs*, whose locus names were underlined, were preferentially expressed in vegetative stages. Two genes labelled by "*" were annotated only by the Rice Annotation Project (http://rapdb.dna.affrc.go.jp/index.html). T1, Leaf (20 days); T2, Shoot; T3, Seedling (four-leaf stage); T4, Pre-emergence inflorescence; T5, Post-emergence inflorescence; T6, Anther; T7, Pistil; T8, Seed (5 DAP); T9, Seed (10 DAP); T10, Embryo (25 DAP); T11, Endosperm (25 DAP). (b) Expression regulation of *VQs* under various abiotic and biotic stresses. The data were originally achieved from Kim et al., 2013 and Li et al., 2014 and were verified by our experiments. "NA" in (A) and (B), not available; "0", "-" and "+", no significantly regulated, down-regulated and up-regulated by a stress, respectively. "+-", up- or down-regulated at different time points of stress treatment for the same gene. *Xoo, Xanthomonas oryzae pv. Oryzae; Xoc, Xanthomonas oryzae pv. Oryzicola; Mo, Magnaporthe oryzae.*
