# Supplementary material for: Valine-glutamine (VQ) motif coding genes are ancient and non-plant-specific with comprehensive expression regulation by various biotic and abiotic stresses
Source: BMC Genomics. 2018 May 9;19:342. doi: 10.1186/s12864-018-4733-7 (PMC5941492; doi:10.1186/s12864-018-4733-7)
Supplement: Supplementary file 10 — Figure S4. Expression profiling of Arabidopsis VQs among different tissues and under various abiotic / biotic stresses and hormones. (PDF 75 kb) [file 12864_2018_4733_MOESM10_ESM.pdf]

(a)

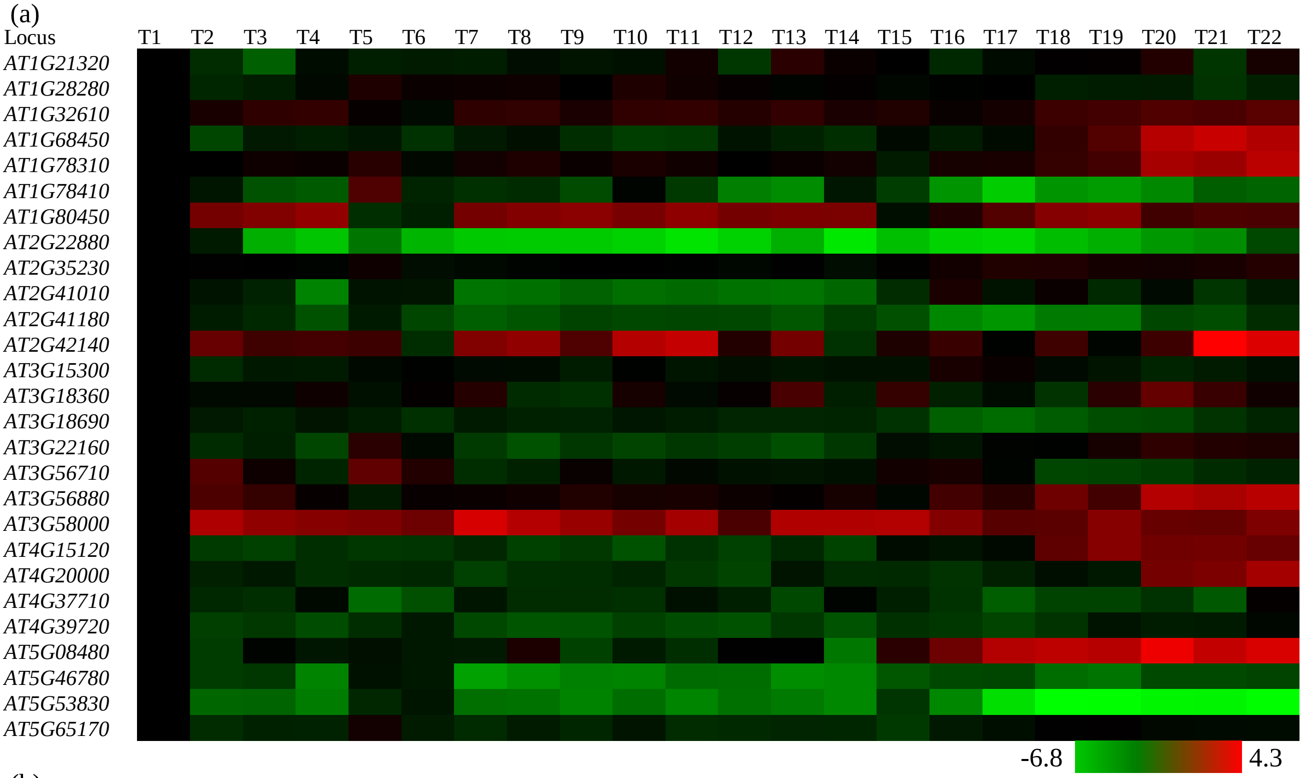

(b)

Locus	Abiotic stresses			Hormone		Biotic stresses	
	Cold	Drought	NaCl	ABA	JA	*Golovinomyces orontii*	*Ralstonia solanacearum*
AT1G21320	NOT	NOT	NOT	NOT	+	0	0
AT1G28280	NOT	NOT	NOT	NOT	0	0	0
AT1G32610	DIFF	NOT	NOT	NOT	0	0	0
AT1G68450	NOT	NOT	NOT	DIFF	-	0	-
AT1G78310	NOT	NOT	DIFF	NOT	-	0	0
AT1G78410	DIFF	DIFF	NOT	NOT	-	0	0
AT1G80450	NOT	NOT	DIFF	DIFF	0	0	+
AT2G22880	DIFF	DIFF	DIFF	DIFF	+	0	0
AT2G35230	DIFF	NOT	NOT	DIFF	0	0	0
AT2G41010	DIFF	DIFF	DIFF	NOT	-	0	+
AT2G41180	NOT	NOT	NOT	DIFF	+	-	-
AT2G42140	NOT	NOT	NOT	NOT	0	0	0
AT3G15300	NOT	NOT	NOT	NOT	-	0	-
AT3G18360	NOT	NOT	NOT	NOT	+	0	0
AT3G18690	NOT	NOT	NOT	NOT	+	0	+
AT3G22160	NOT	NOT	NOT	NOT	+	0	0
AT3G56710	NOT	NOT	NOT	NOT	-	-	0
AT3G56880	DIFF	DIFF	DIFF	DIFF	0	0	0
AT3G58000	NOT	NOT	NOT	NOT	-	0	0
AT4G15120	NOT	NOT	NOT	NOT	+	0	-
AT4G20000	DIFF	NOT	NOT	NOT	-	0	-
AT4G37710	NOT	NOT	DIFF	DIFF	0	0	0
AT4G39720	NOT	NOT	NOT	NOT	-	0	-
AT5G08480	NOT	NOT	NOT	NOT	-	0	-
AT5G46780	NOT	NOT	NOT	NOT	+	0	0
AT5G53830	NOT	DIFF	DIFF	NOT	-	0	0
AT5G65170	NOT	NOT	NOT	NOT	-	0	-
Total (%)	25.9	18.5	25.9	25.9	74.1	7.4	37.0

Additional file 10: Figure S4. Expression profiling of Arabidopsis *VQs* among different tissues and under various abiotic / biotic stresses and hormones. (a) Transcriptomic abundance of VQ genes among different developmental stages. T1, hypocotyl, ATGE_2; T2, shoot apex, vegetative + young leaves, ATGE_4; T3, shoot apex, vegetative, ATGE_8; T4, shoot apex, transition (before bolting, ATGE_8); T5, stem, 2nd internode, ATGE_27; T6, 1st node, ATGE_28; T7, shoot apex, inflorescence (after bolting, ATGE_29); T8, shoot apex, inflorescence (after bolting, ATGE_46); T9, shoot apex, inflorescence (after bolting, ATGE_47); T10, shoot apex, inflorescence (after bolting, ATGE_48); T11, shoot apex, inflorescence (after bolting, ATGE_49); T12, shoot apex, inflorescence (after bolting, ATGE_50); T13, shoot apex, inflorescence (after bolting, ATGE_51); T14, shoot apex, inflorescence (after bolting, ATGE_52); T15, siliques at Stage 3; T16, siliques at Stage 4; T17, siliques Stage 5; T18, Seeds at Stage 6; T19, Seeds at Stage 7; T20, Seeds at Stage 8; T21, Seeds at Stage 9; T22, Seeds at Stage 10. Data (T1-T14) were achieved from GSE5633 and data (T15-T22) were achieved from GSE5634. (b) Regulated expression of VQ genes under various abiotic/biotic stresses and hormones. Data (cold, drought, NaCl and ABA) were achieved from GSE61884. Expression data under JA, *Golovinomyces orontii* and *Ralstonia solanacearum* were achieved from GSE61884, GSE40973 and GSE92631, respectively.
