# Supplementary material for: Valine-glutamine (VQ) motif coding genes are ancient and non-plant-specific with comprehensive expression regulation by various biotic and abiotic stresses
Source: BMC Genomics. 2018 May 9;19:342. doi: 10.1186/s12864-018-4733-7 (PMC5941492; doi:10.1186/s12864-018-4733-7)
Supplement: Supplementary file 11 — Figure S5. Gene set enrichment analysis of co-expressed genes with VQs. (PDF 128 kb) [file 12864_2018_4733_MOESM11_ESM.pdf]

(a)

Ontology	GO term	Description	01g17050	02g15290	03g20330	03g47280	06g33970	06g40090	06g41450	08g31660
C	GO:0005576	extracellular region						+	+	
C	GO:0005618	cell wall								+
C	GO:0009536	plastid							+	
C	GO:0009579	thylakoid							+	
C	GO:0016020	membrane							+	
C	GO:0030312	external encapsulating structure						+	+	+
F	GO:0003677	DNA binding		+		+				
F	GO:0003700	transcription factor activity	+	+	+	+	+			
F	GO:0008289	lipid binding						+		+
F	GO:0019825	oxygen binding		+	+	+				
F	GO:0030528	transcription regulator activity	+	+	+	+	+			
P	GO:0006091	generation of metabolites and energy							+	
P	GO:0006950	response to stress	+	+	+	+	+	+	+	+
P	GO:0009607	response to biotic stimulus		+	+	+	+		+	
P	GO:0009628	response to abiotic stimulus	+			+			+	
P	GO:0009719	response to endogenous stimulus			+	+				
P	GO:0015979	photosynthesis							+	
P	GO:0019748	secondary metabolic process				+			+	
P	GO:0050896	response to stimulus	+	+	+	+		+	+	+

(b)

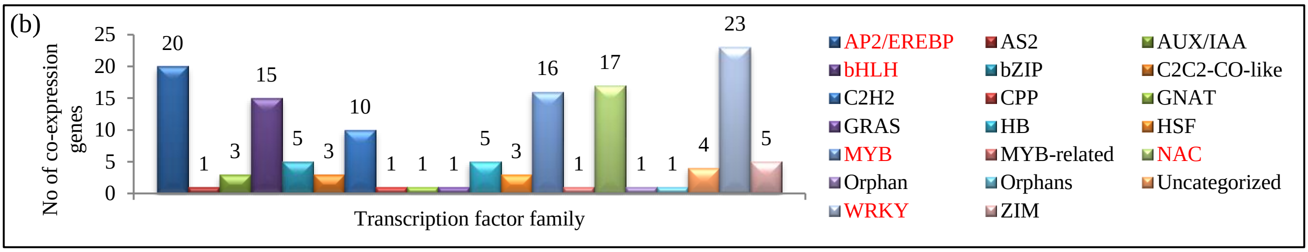

Additional file 11: Figure S5. Gene set enrichment analysis of co-expressed genes with VQs. (a) Over-representative (enrichment) GO terms among co-expressed genes with 8 *VQs*. The prefix "LOC_Os" in each locus name was omitted for convenience. (b) A general overview of co-expressed genes encoding transcription factors and their family names. The five family names were highlighted with red fonts, which contained 15 or more co-expressed members in each family.
