# Supplementary material for: Valine-glutamine (VQ) motif coding genes are ancient and non-plant-specific with comprehensive expression regulation by various biotic and abiotic stresses
Source: BMC Genomics. 2018 May 9;19:342. doi: 10.1186/s12864-018-4733-7 (PMC5941492; doi:10.1186/s12864-018-4733-7)
Supplement: Supplementary file 12 — Table S7. Primer sequences used in this study. (PDF 9 kb) [file 12864_2018_4733_MOESM12_ESM.pdf]

Additional file 12: Table S7. Primer sequences used in this study

Family Name/annotation	Primer Name	Locus name	Forward primer	Reverse primer	Length (BP)
VQ motif encoding gene from fungus	XM_007869238.1	XM_007869238.1	ATGGCTCCTCAGACACTCCCCGTCCTCATC	CTAAGCAAAGATCCGACTGAAGTACTCCTT	1671
Actin 1	XM_007864468.1	XM_007864468.1	AAGCCCAGGGTATCCACGACACGA	ACGCGAGCCGAAAACCACCATC	408
