# Supplementary material for: Valine-glutamine (VQ) motif coding genes are ancient and non-plant-specific with comprehensive expression regulation by various biotic and abiotic stresses
Source: BMC Genomics. 2018 May 9;19:342. doi: 10.1186/s12864-018-4733-7 (PMC5941492; doi:10.1186/s12864-018-4733-7)
Supplement: Supplementary file 13 — The alignment of VQ motif sequences from 12 plant species. (PDF 106 kb) [file 12864_2018_4733_MOESM13_ESM.pdf]

Additional file 13. The alignment of VQ motif sequences from 12 plant species

```
CLUSTAL 2.0.12 multiple sequence alignment


AC194056.3_FG008                    --KIIE--TVHVEADRYS-FKSIVQRLTGRDAVVGG---
Si003551m.g                         --KIIE--TVHVEADRSS-FKSVVQRLTGRDAVASD---
LOC_Os01g46440                      --KIIE--TVHIEADSAE-FKSIVQRLTGKDAVAGG---
ORGLA01G0202500                     --KIIE--TVHIEADSAE-FKSIVQRLTGKDAVAGG---
BGIOSGA001106                       --KIIE--TVHIEADSAE-FKSIVQRLTGKDAVAGG---
OB01G34080                          --KIIE--TVHIEADSAE-FKSVVQRLTGKDAVAGG---
GRMZM5G814101                       --KIIE--TVYVEAGTADDFKSVVQRFTGKDAAAEL---
Si024902m.g                         --KIIE--TVYVEADTADDFKSVVQRLTGKDAIAEP---
BGIOSGA020407                       --KIIE--TVYVEADTADDFKSVVQRLTGKDAVAGD---
Sb09g029150                         --KIIE--TVYVEADTADDFKSVVQRLTGKDAAVAA---
BGIOSGA006776                       --TFIE--TQFVTSDAAG-FKSLVQRLTGNDAAVPA---
LOC_Os02g15290                      --TFIE--TQFVTSDAAG-FKSLVQRLTGNDAAVPA---
ORGLA02G0097400                     --TFIE--TQFVTSDAAG-FKSLVQRLTGNDAAVPA---
LOC_Os02g15280                      --TFIE--TQFVTSDAAG-FKSLVQRLTGNDAAVAT---
ORGLA02G0097300                     --TFIE--TQFVTSDAAG-FKSLVQRLTGNDAAVAT---
BGIOSGA006777                       --TFIE--TQFVTSDAAG-FKSLVQRLTGNDAAVAT---
OB02G19620                          --TFIE--TQFVTSDAAG-FKSLVQRLTGNDATAAH---
GRMZM2G153597                       --TYIE--TQFVTSDAAG-FKDLVQRLTGRSATPPV---
Sb04g009150                         --TYIE--TQFVTSDEAG-FKDLVQRLTGRSAAPAP---
Si018158m.g                         --TYIE--TRFVTSDAAG-FKDLVQRLTGRSPTAAG---
Si019438m.g                         --TYIE--TQFVTSDAAG-FKDLVQRLTGRSPTAAS---
BRADI3G09510                        --TYIE--TQFVTSDAAS-FKSVVQSLTGKSAEPAR---
LOC_Os06g33970                      --KFIE--TQFVSSDAAS-FKAVVQRLTGQSAPSPS---
ORGLA06G0142600                     --KFIE--TQFVSSDAAS-FKAVVQRLTGQSAPSPS---
BRADI1G38740                        --KFIE--TQFVSSDAAS-FKSVVQRLTGKHSQMPP---
GRMZM2G138370                       --KFIE--TQFISSDAAS-FKAVVQRLTGKSPAASS---
Sb10g020870                         --KFIE--TQFISSDAAS-FKAVVQRLTGKSPAPAS---
Si007480m.g                         --KFIE--TRFISSDAAS-FKAVVQRLTGKSSSLAA---
BGIOSGA018594                       --KYIV--TRFVEADAAE-FKSVVQSLTGKDSTAAT---
LOC_Os05g12090                      --KYIV--TRFVEADAAE-FKSVVQSLTGKDSTAAT---
ORGLA05G0061400                     --KYIV--TRFVEADAAE-FKSVVQSLTGKDSTAAT---
Si024489m.g                         --KFIV--TKYVDADAAH-FKSVVQSLTGKNSTAAA---
GRMZM5G800535                       --KHIV--TREVSTDQAN-FKDVVQWLTGKDSAAAR---
GRMZM5G873101                       --KHIV--TREVSTDQAN-FKDVVQWLTGKDS-------
OB06G13460                          --THVV--TAEVSADEAS-FKDVVQRLTGKDSAAAR---
AT1G17147                           --VFIN--TQYVQTDARS-FKTIVQELTGKNAVVAD---
fgenesh2_kg.1__1870__AT1G17147      --VFIN--TQYVQTDARS-FKTVVQELTGKNAVVAD---
Bra025998                           --VFIN--TQYVQTDARS-FKTVVQELTGKNAIVAD---
AT1G78410                           --VFIN--TQYVETDARS-FKTVVQELTGKNAIVAA---
fgenesh2_kg.2__2136__AT1G78410      --VFIN--TQYVETDARS-FKTVVQELTGKDAIVAA---
Bra008359                           --VFIN--TQYVETDARS-FKNVVQELTGKDAIVAA---
Bra035035                           --VFIN--TQYVETDARS-FKNVVQELTGKDAIVAA---
Bra003642                           --VFIN--TQYVETDTRS-FKTVVQELTGKDAIVAP---
Bra016616                           --VFIN--TRYIQTDARS-FKSIVQELTGKNAVVAE---
GLYMA08G18820                       --VIIN--TQYVETDATS-FKSVVQKLTGKDSDNLD---
GLYMA15G40000                       --VIIN--TQYVETDATS-FKSVVQKLTGKDSDILD---
POPTR_0011s09810                    --VIIN--TEYVQTDASS-FKSVVQKLTGKDSAPSG---
POPTR_0001s38750                    --VIIN--TQYVETDVRS-FKSVVQEFTGKDSAPPG---
GLYMA05G27220                       --VHIE--TRYVETDAIH-FRDVVQHLTGKNSSTTN---
BGIOSGA021871                       --THIV--TARVSADEAS-FRDVVQRLTGGAAGNGL---
Al_scaffold_0006_776                --CKPV--TTFVQTDTNT-FREIVQRLTGPSENNAA---
Al_scaffold_0487_2                  --CKPV--TTFVQTDTNT-FREIVQRLTGPSENNAA---
scaffold_600878.1                   --CKPV--TTFVQTDTNT-FREIVQRLTGPSENNAA---
AT5G08480                           --CKPV--TTFVQTDTNT-FREIVQRLTGPTENNAA---
Bra005995                           --CKPV--TTFVHTDTDT-FREVVQRLTGPSESNAA---
Bra006328                           --CKPV--TTFVHTDTDT-FREVVQHLTGPSESNAA---
```

GLYMA05G33250	--CKPL--TTFVQTNSDA-FREVVQRLTGPSEASAA---
GLYMA08G00850	--CKPL--TTFVQTNSDA-FREVVQRLTGPSEASAA---
POPTR_0004s14180	--GKPI--TTFVQTDTKA-FRDVVQRLTGSSEGDAA---
AT1G80450	--TEPN--TMFVQADPSN-FRNIVQKLTGA-PPDISS--
fgenesh2_kg.2__2337__AT1G80450	--TDPN--TMFVQADPSN-FRNIVQKLTGA-PPDISS--
Bra008473	--TDPN--TMFVQADPSN-FRNIVQELTGA-PPELSP--
Bra035182	--TDPN--TMFVQADPSN-FRNIVQKLTGA-PPELST--
Bra003566	--TDPN--TMFVQADPSN-FRNIVQKLTGASPPELS---
GLYMA07G03240	--TTPN--TTFVQANPSN-FRAVVQKLTGA-SDDPSA--
GLYMA08G22860	--TTPN--TTFVQADPSN-FRAVVQKLTGA-SDDPSA--
Vv09s0002g07540	--SSNN--TTFVQADPSN-FRAVVQHLTGA-SPDSAS--
BGIOSGA014233	--VDTN--TTFVQADPAT-FRALVQKLTGA-PGSGGS--
LOC_Os04g55240	--VDTN--TTFVQADPAT-FRALVQKLTGA-PGSGGS--
ORGLA04G0235300	--VDTN--TTFVQADPAT-FRALVQKLTGA-PGSGGS--
OB04G34240	--VDTN--TTFVQADPTT-FRALVQKLTGA-PGSGKT--
GRMZM2G023921	--VDAN--TTFVQADPAT-FRALVQKLTGA-APAAGG--
GRMZM2G064903	--VDAN--TTFVQADPAT-FRALVQKLTGA-ATDDAA--
Si011801m.g	--VDAN--TTFVQADPAT-FRALVQKLTGA-PAEKKP--
SELMODRAFT_414696	--HCTA--TTFVQADASS-FRDLVQRLTGA-SDDSIK--
SELMODRAFT_448206	--HCTA--TTFVQADASS-FRDLVQRLTGA-SDDSIK--
Vv04s0008g06930	--PPTS--TTFVQADATT-FRDLVQKLTGA-AVDSPE--
POPTR_0006s28240	--LTSP--TTFVQADINT-FRDLVQKLTGL-ASDTQR--
POPTR_0018s02810	--PTSP--TTFVQADTNS-FRDLVQKLTGL-ASDTQQ--
PP1S138_111V6	--FAST--TTFVQVDTSS-FRELVQKLTGA-SDSDVE--
PP1S42_199V6	--FASI--TTFVQVDTSS-FRELVQKLTGA-SDSDVE--
SELMODRAFT_415509	--QLAA--TTFIQADVLT-FREVVQKHTGA-AEDAQD--
SELMODRAFT_438797	--QLAA--TTFIQADVLT-FREVVQKHTGA-AEDAQD--
AT1G28280	--NPYP--TTFVQADTSS-FKQVVQMLTGSAERPKH---
scaffold_103160.1	--NPYP--TTFVQADTSS-FKQVVQMLTGSAERPKH---
Bra030082	--NPYP--TTFVQADTSS-FKQVVQMLTGSSDRPKQ---
GLYMA07G31780	--NPYP--TTFVQADTTS-FKQVVQMLTGSTQTAKQ---
GLYMA13G24700	--NPYP--TTFVQADTNS-FKQVVQMLTGSTQTAKQ---
POPTR_0001s40990	--NPYP--TTFVQADTST-FKQVVQMLTGSTETAKQ---
POPTR_0011s11910	--NPYP--TTFVQADTST-FKQVVQMLTGSTETAKQ---
GLYMA13G31180	--NSYP--TTFVQADTTS-FKQVVQMLTGSSETAKQ---
GLYMA15G08160	--NPYP--TTFVQADTSS-FKQVVQMLTGSSETAKQ---
AT5G53830	--NPYP--TTFVQADTST-FKQVVQMLTGSSTDTTT---
fgenesh2_kg.8__1275__AT5G53830	--NPYP--TTFVQADTST-FKQVVQMLTGSSTDNTT---
Bra022675	--NPYP--TTFVQADTST-FKQVVQMLTGSSTDTKT---
GLYMA06G36640	--NPYP--TTFVQADTST-FKHVVQMLTGSSETTNP---
GLYMA12G24250	--NPYP--TTFVQADTST-FKQVVQMLTGSSETTKP---
GLYMA12G35380	--NPYP--TTFVQADTST-FKQVVQMLTGSSDTTKQ---
GLYMA13G35130	--NPYP--TTFVQADTST-FKQVVQMLTGSSDTTKQ---
Bra003032	--NPYP--TTFVQADTST-FKQVVQMLTGSSSTDTN---
POPTR_0004s04460	--NPYP--TTFVQADSSS-FKQVVQMLTGSPKPKPT---
POPTR_0011s05350	--NPYP--TTFVQADTSS-FKQVVQMLTGSPKPAST---
Vv19s0014g02490	--NPYP--TTFVQADANS-FKQVVQRLTGSSKPTQD---
Bra021096	--DHYP--TTFVQADTSS-FKQVVQMLTGSSSPRSP---
fgenesh2_kg.3__1663__AT3G15300	--DHYP--TTFVQADTSS-FKQVVQMLTGSSSPRSP---
AT3G15300	--DHYP--TTFVQADSSS-FKQVVQMLTGSSSPRSP---
Bra027262	--DHYP--TTFVQADTST-FKQVVQMLTGTSSPRSP---
BGIOSGA020204	--TPFP--TTFVQADTAS-FKQVVQMLTGSDTTPPS---
LOC_Os05g44270	--TPFP--TTFVQADTAS-FKQVVQMLTGSDTTPPS---
ORGLA05G0201700	--TPFP--TTFVQADTAS-FKQVVQMLTGSDTTPPS---
OB05G30560	--TPFP--TTFVQADTAS-FKQVVQMLTGSDTPSST---
GRMZM2G174558	--TPFP--TTFVQADTAN-FKQVVQRLTGSDTPPPA---
Si023066m.g	--TPFP--TTFVQADTAN-FKQVVQRLTGSDTPSSA---
GRMZM2G180668	--TPFP--TTFVQADTAS-FKQVVQRLTGSDTPPPP---
Sb09g025820	--TPFP--TTFVQADTAN-FKQVVQRLTGSDTPPTP---
Sb03g034490	--TPFP--TTFVQADTTS-FKQVVQMLTGAEQPAKN---
Si002727m.g	--TPFP--TTFVQADTTS-FKQVVQMLTGAEQPAKN---

```
GRMZM2G314520                       --TPFP--TTFVQADTTS-FKQVVQMLTGAEQPANN---
LOC_Os01g54400                      --TPFP--TTFVQADTAS-FKQVVQMLTGAEQPSKN---
OB01G39640                          --TPFP--TTFVQADTAS-FKQVVQMLTGAEQPSKN---
ORGLA01G0251600                     --TPFP--TTFVQADTAS-FKQVVQMLTGAEQPSKN---
BRADI2G49740                        --TPFP--TTFVQADTNS-FKQVVQMLTGSEQSSKN---
BRADI2G19600                        --TPFP--TTFVQADTAS-FKQIVQMLTGAADMPPP---
GRMZM2G055404                       --NPFP--TTFVQADTTS-FKQVVQILTGTPETAAA---
Sb03g002570                         --NPFP--TTFVQADTTS-FKQVVQILTGTPETAAA---
Si004264m.g                         --NPFP--TTFVQADTTS-FKQVVQILTGTPETAAA---
GRMZM2G151909                       --NTFP--TTFVQADTNS-FKQVVQILTGTPETAEA---
AT2G33780                           --DMYE--TTFIRTDPSS-FKQVVQLLTGIPKNPTH---
fgenesh2_kg.4__1390__AT2G33780      --NMID--TTFIRTDPSS-FKQVVQLLTGISKTPPH---
LOC_Os08g31660                      --RRAP--TTVLTTDTSN-FRAMVQEFTG-IPSPPFA--
ORGLA08G0125500                     --RRAP--TTVLTTDTSN-FRAMVQEFTG-IPSPPFA--
BGIOSGA028654                       --RRAP--TTVLTTDTSN-FRAMVQEFTG-IPSPPFA--
GRMZM2G126413                       --RRAP--TTVLTTDTSN-FRAMVQEFTG-IPSPPFA--
AT5G65170                           --RRAP--TTVLTTDTSN-FRAMVQEFTG-NPSTPFT--
Bra024362                           --RRAP--TTVLTTDTSN-FRAMVQEFTG-NPSTPFT--
fgenesh2_kg.8__2558__AT5G65170      --RRAP--TTVLTTDTSN-FRAMVQEFTG-NPSTPFT--
Bra037806                           --RRAP--TTVLTTDTSN-FRAMVQEFTG-NPSNPFS--
Bra031876                           --RRAP--TTILTTDTSN-FRAMVQEFTG-SPSNPFA--
BRADI3G35600                        --RRAP--TTVLTTDTSN-FRAMVQEFTG-VPSPPFA--
BGIOSGA030630                       --RRAP--TTVLTTDTSN-FRAMVQEFTG-IPSPPFV--
LOC_Os09g20460                      --RRAP--TTVLTTDTSN-FRAMVQEFTG-IPSPPFV--
ORGLA09G0058000                     --RRAP--TTVLTTDTSN-FRAMVQEFTG-IPSPPFV--
Sb02g023320                         --RRAP--TTVLTTDTSN-FRAMVQEFTG-IPSPPFG--
BRADI4G29080                        --RRAP--TTVLTTDTSN-FRAMVQEFTG-IPNPPFA--
GRMZM2G066599                       --RRAP--TTVLTTDTSN-FRAMVQEFTG-IPAPPFA--
Si013900m.g                         --RRAP--TTVLTTDTSN-FRAMVQEFTG-IPAPPFA--
AT1G35830                           --RRAP--TTVLTTDTSN-FRAMVQEFTG-VPASPFS--
fgenesh2_kg.1__3657__AT1G35830      --RRAP--TTVLTTDTSN-FRAMVQEFTG-VPASPFS--
GLYMA14G34680                       --RRAP--TTVLTTDTSN-FRAMVQEFTG-IPAPPFS--
Vv18s0001g05400                     --RRAP--TTVLTTDTSN-FRAMVQEFTG-IPAPPFS--
GLYMA04G11200                       --RRAP--TTVLTTDTNN-FRSMVQEFTG-IPAPPFS--
GLYMA06G10960                       --RRAP--TTVLTTDTNN-FRSMVQEFTG-ISAPPFS--
GLYMA06G10950                       --------RRAPTTDTNN-FRSMVQEFTG-IPAPPFS--
LOC_Os09g20020                      --TRAS--WRTVTTDPSN-FRAMVQEFTG-IPSPPFV--
POPTR_0002s09550                    --RRAP--TTVLTTDTSN-FRQMVQEFTG-IPAPPFS--
POPTR_0005s16970                    --RRAP--TTVLTTDTSN-FRQMVQEFTG-IPAPPFT--
GLYMA11G38180                       --RRAP--TTVLTTDTTN-FRAMVQEFTG-IPAPPFT--
GLYMA18G02140                       --RRAP--TTVLTTDTTN-FRAMVQEFTG-IPAPPFT--
GLYMA05G31340                       --RRAP--TTVLTTDTTN-FRAMVQEFTG-IPAPPFT--
POPTR_0007s05600                    --RRAP--TTVLTTDTTN-FRAMVQEFTG-IPAPPFT--
POPTR_0005s07820                    --RRAP--TTVLSTDTTN-FRAMVQEFTG-IPAPPFT--
GLYMA08G14580                       --RRAP--TTVLTTDTTN-FRAMVQEFTG-IPAQPFT--
Vv00s0304g00030                     --RRAP--TTVLTTDTTN-FRAMVQEFTG-IPAQPFT--
AT4G39720                           --RRAP--TTVLTTDTSN-FRAMVQEFTG-IPAPPLF--
fgenesh2_kg.7__187__AT4G39720.      --RRAP--TTVLTTDTSN-FRAMVQEFTG-IPAPPLF--
Bra010666                           --RRAP--TTVLMTETSN-FRAMVQEFTG-IPSPPLF--
Bra011838                           --RRAP--TTVLKTDTSN-FRAMVQEFTG-IPSPPLF--
BGIOSGA023883                       --WRPP--TTVLTTDASN-FRAMVQEFTG-FPATPPF--
ORGLA07G0176400                     --RRPP--TTVLTTDASN-FRAMVQEFTG-FPATPPF--
LOC_Os07g43140                      --RRPP--TTVLTTDASN-FRAMVQEFTG-FPATPPL--
OB07G28250                          --RRPP--TTVLTTDASN-FRAMVQEFTG-FPAPPPL--
GRMZM2G316033                       --RRPP--TTVVTTDTSN-FRAMVQEFTG-IPAPPPP--
Sb02g039780                         --RRPP--TTVVTTDTSN-FRAMVQEFTG-IPAPPPF--
Si033394m.g                         --RRPP--TTVLTTDTSN-FRAMVQEFTG-IPAPPPF--
BGIOSGA026896                       --RRPP--TTVLTTDTSN-FRAMVQEFTG-FPAPPFA--
GRMZM2G035531                       --RRPP--TTVLTTDTSN-FRAMVQEFTG-FPAPPFA--
BRADI1G60860                        --RRPP--TTVLTTDTSN-FRAMVQEFTG-FPAPPFA--
LOC_Os03g26990                      --RRPP--TTVLTTDTSN-FRAMVQEFTG-FPAPPFA--
```

```
Si036960m.g                           --RRPP--TTVLTTDTSN-FRAMVQEFTG-FPAPPFA--
OB03G29000                            --RRPP--TTVLTTDTSN-FRAMVQEFTG-FPAPPFV--
AC207043.3_FG002                      --RRAP--VTLLNTDTSN-FRAMVQQFTG-VPPGPYG--
GRMZM2G010333                         --RRAP--VTLLNTDTAN-FRAMVQQLTG-VPPGPCG--
BRADI3G05410                          --RRAP--VTLLNTDTSN-FRAMVQQFTG-IPSGPYG--
Sb04g004860                           --RRAP--VTLLNTDTSN-FRAMVQQFTG-IPSGPYG--
BGIOSGA023429                         --RRAP--VTLLNTDTTN-FRAMVQQFTG-IPAPPAG--
LOC_Os06g45570                        --RRAP--VTLLNTDTTN-FRAMVQQFTG-IPAPPAG--
ORGLA06G0201800                       --RRAP--VTLLNTDTTN-FRAMVQQFTG-IPAPPAG--
Sb10g026640                           --RRAP--VTLLNTDTAN-FRAMVQQFTG-IPAPPAG--
Si008810m.g                           --RRAP--VTLLNTDTAN-FRAMVQQFTG-IPAPPAG--
GRMZM2G333049                         --RRAP--VTLLNTDTAN-FRAMVQQFTG-IPAPPAG--
BRADI1G32060                          --RRAP--VTLLNTDAAN-FRAMVQQFTG-VPGPVVS--
GRMZM2G420715                         --RRAP--VTLLNTDAAN-FRAMVQQFTG-VPAPLAG--
LOC_Os02g07690                        --RRAP--VTLLNTDTSN-FRAMVQQFTG-IPTPPYA--
AT3G22160                             --RRTP--TTLLNTDTSN-FRAMVQQYTG-GPSAMAF--
scaffold_302646.1                     --RRTP--TTLLNTDTSN-FRAMVQQYTG-GPSAMAF--
Bra023849                             --RRTP--TTLLNTDTSN-FRAMVQQYTG-GPSAMAF--
AT4G15120                             --RRTP--TTLFNTDTAN-FRAMVQQFTG-GPSAVAF--
fgenesh2_kg.7__2809__AT4G15120        --RRTP--TTLFNTDTAN-FRAMVQQFTG-GPSAVAF--
Bra039565                             --RRTP--TTLLNTDTAN-FRAMVQQFTG-GPSAMAF--
GLYMA13G10840                         --RRTP--TTLLNTDTTN-FRAMVQQFTG-GPSAPFA--
POPTR_0006s00810                      --RRTP--TTLLNTDTTN-FRAMVQQFTG-GPSAPFA--
POPTR_0016s01940                      --RRTP--TTLLNTDTTN-FRAMVQQFTG-GPSAPFA--
GLYMA20G15230                         --RRTP--TTLLNTDTTN-FRAMVQQFTG-GPSAPYA--
GLYMA14G00570                         --RRTP--TTLLNTDTSN-FRAMVQQFTG-APSAPDM--
BGIOSGA009072                         --RRSS--TTVVATDVSN-FRSMVQELTG-FPAAAIF--
LOC_Os02g51740                        --RRSS--TTVVATDVSS-FRSMVQELTG-FPAAAIF--
ORGLA02G0283900                       --RRSS--TTVVATDVSN-FRAMVQELTG-FPAAAIF--
BRADI3G58880                          --RRSS--TTVVATDVGN-FRAMVQELTG-FPPAAIF--
BRADI3G58890                          --RRSS--TTVVATDVGN-FRAMVQELTG-FPPAAIF--
Sb04g027600                           --RRSS--TTVVATDVNN-FRAMVQELTG-FPPAAIF--
GRMZM2G061941                         --RRSS--TTVVATDVAN-FRAMVQELTG-FPPAAIF--
GRMZM2G322950                         --RRSS--TTVVATDVSN-FRAMVQELTGFFPPPAA---
SELMODRAFT_407942                     --RRPP--TTVLEADSAN-FRSMVQHLTG-IPAPPPM--
SELMODRAFT_417817                     --RRPP--TTVLEADSAN-FRSMVQHLTG-IPAPPPM--
SELMODRAFT_419311                     --RKPP--TTVLEADSSN-FRAMVQQLTG-IPSVPFL--
SELMODRAFT_446251                     --RKPP--TTVLEADSSN-FRAMVQQLTG-IPSVPFL--
AT4G20000                             --RAIP--TTLLNANPSN-FRALVQKFTG-RSAGGES--
fgenesh1_pm.C_scaffold_7001790        --RAIP--TTLLNANPSN-FRALVQKFTG-RSAGGGS--
Bra013438                             --RAVP--TTLLNANPSN-FRALVQKFTG-RSSGGES--
GLYMA05G26340                         --KSTP--ITLLKANTSN-FRALVQQFTG-CPTTTTM--
GLYMA08G09250                         --KSTP--ITLLKANTSN-FRALVQQFTG-CPTTTAM--
GLYMA09G05700                         --KRTP--TTLLNANPTN-FRALVQQFTG-CPRTTMS--
GLYMA15G16990                         --KKTP--TTLLNANTTN-FRALVQQFTG-CHSTTMP--
POPTR_0003s07340                      --KKTP--ATLINASTAN-FRALVQQFTG-CPPSTSI--
POPTR_0001s15890                      --KKTP--STLLTATTAN-FRALVQQFTG-CPSNPIS--
Vv01s0011g01350                       --RRIP--ATLLTANTTN-FRALVQQFTG-RPTTPFS--
AT2G41010                             --KKSQ--TTFITADPSN-FRQMVQQVTG-AKYIDDS--
fgenesh2_kg.4__2228__AT2G41010        --KKSQ--TTFITADPSN-FRQMVQQVTG-SRYIDDS--
Bra016956                             --KKPQ--TTFITADPSN-FRQMVQQVTG-AKCINND--
AT3G56880                             --KKSQ--TTFITADAAN-FRQMVQQVTG-AKFLGSS--
fgenesh2_kg.5__2121__AT3G56880        --KKSQ--TTFITADAAN-FRQMVQQVTG-AKFLGSS--
Bra014665                             --KKSQ--TTFITADAAN-FRQMVQQVTG-AKFIGSS--
Bra007279                             --KKSQ--TTFITADAAN-FRQMVQQVTG-AKYNGSP--
POPTR_0006s03090                      --KRSQ--TTFITADPAN-FRQMVQQVTG-VRFGNSQ--
Vv08s0040g00540                       --KRSQ--TTFITADPAN-FRQMVQQVTG-VRFGNSQ--
POPTR_0016s02940                      --KRSQ--TTFITADPAN-FRQMVQQVTG-VRFNNSQ--
GLYMA03G27560                         --KRSQ--TTFITADPAN-FRQMVQQVTG-VRFGGAG--
BGIOSGA001894                         --RRAH--TTYITADPAD-FRRMVQEITG--FPVPGAH-
LOC_Os01g17050                        --RRAH--TTYITADPAD-FRRMVQEITG--FPVPGAH-
```

GRMZM2G378442	--RRPQ--TTYITADPAN-FQRMVQEITG--LPAPGPS-
Sb03g011200	--RRPQ--TTYITADPAN-FRRMVQEITG--LPVPVSV-
BGIOSGA034725	--RRLP--TTYISADPAN-FRRMVHQVTG-ADDLPPP--
BGIOSGA036930	--RRLP--TTYISADPAN-FRRMVHQVTG-ADDLPPP--
LOC_Os11g03660	--RRLP--TTYISADPAN-FRRMVHQVTG-ADDLPPP--
LOC_Os12g03420	--RRLP--TTYISADPAN-FRRMVHQVTG-ADDLPPP--
ORGLA11G0015900	--RRLP--TTYISADPAN-FRRMVHQVTG-ADDLPPP--
ORGLA12G0014400	--RRLP--TTYISADPAN-FRRMVHQVTG-ADDLPPP--
Si012398m.g	--RKLP--TTYIAANPAS-FRRMVHQVTG-ADDLPMP--
GRMZM2G180262	--RKLP--TTYISADAAS-FRRMVHQVTG-AG-VGADV-
Sb08g001595	--RKLP--TTYISADAAS-FRRMVHQVTG-ADDVVAA--
BRADI4G43770	--RRTP--TTYIRAGPAE-FRRMVHQVTG-ADDIDLL--
GRMZM2G147443	--RRLP--TTYINADPAS-FRRMVHQATGAAEDLPP---
GRMZM2G325208	--RRLP--TTYINADPAS-FRRMVHQATGAAEDLPP---
BRADI2G10700	--RRAP--TTYISAAPAD-FRRMVQEVTGLAVPAGA---
BGIOSGA010844	--KRAP--TTYISTDAAT-FRIMVQQVTG-AQVEPQD--
LOC_Os03g20440	--KRAP--TTYISTDAAT-FRIMVQQVTG-AQVEPQD--
GRMZM2G128644	--KRAP--TTYISTDAAT-FRAMVQRVTG-ADEADLQ--
Sb01g036940	--KRVP--TTYISTDAAT-FRAMVQRVTG-ADEADLQ--
GRMZM2G374336	--KRAP--TTYISTDAAT-FRAMVQRVTG-ADEANLQ--
GRMZM5G864059	--KRAP--TTYISTDAAT-FRAMVQRVTG-ADEANLQ--
GRMZM5G849527	--KRAP--TMYISTDAAT-FRAMVQRVTG-ADEANLQ--
GRMZM2G014839	--KRAP--TTYISADAAT-FRAMVQRVTG-ADEDDLQ--
BRADI1G63980	--KRAP--TTYISTDAAT-FRLMVQHVTG-ADADTLQ--
BRADI1G17290	--KRAP--TTYISTDTAN-FRFMVQHVTG-ALHHDVD--
BGIOSGA026421	--KRAP--TTYISTDPAN-FRLMVQHVTG-VQADPAS--
LOC_Os07g48710	--KRAP--TTYISTDPAN-FRLMVQHVTG-VQADPAS--
ORGLA07G0212400	--KRAP--TTYISTDPAN-FRLMVQHVTG-VQADPAS--
Sb02g043570	--KRAP--TTYISTDPAN-FRLMVQQITG-VQAGVDD--
Si031131m.g	--KRAP--TTYISTDPAN-FRIMVQQITG-VQADIEM--
GRMZM2G101409	---RAPT-TTYISTDTAN-FRLMVQQITG-AQEADGG--
PP1S152_19V6	--RRTP--TTVLETNPSE-FRDTVQRLTG-IPSMPNQ--
PP1S49_252V6	--RRTP--VTVLETNPWE-FRDTVQRLTG-LRAAPNP--
PP1S134_155V6	--RRVP--TTILETSCQD-FRDAVQKLTG-FHRPKPA--
PP1S49_255V6	--RRTP--TTILKTSCQE-FREAVQKLTG-FHRPTMT--
PP1S206_20V6	--KKVP--TTVLDSSSEN-FMTLVQKLTG-SADEVVA--
PP1S81_209V6	--KKVP--TTILEASSTH-FMELVQKLTG-STEEKRA--
Sb03g037575	-YISNPM---RVKTS-AAGFRALVQELTGRD-ADPSK--
Si004625m.g	-YISNPM---RVKTS-AAGFRALVQELTGRD-ADPSK--
BRADI2G52877	-YISNPM---RVKTS-AAGFRALVQELTGRD-ADPSK--
AC203294.3_FG012	-YISNPV---RVKTS-AAGFRALVQELTGRD-ADPSK--
BGIOSGA004652	-YISNPM---RVKTS-AAGFRALVQELTGRN-ADPSK--
LOC_Os01g59410	-YISNPM---RVKTS-AAGFRALVQELTGRN-ADPSK--
LOC_Os05g32460	-YISNPM---RVRTS-AAGFRALVQELTGRN-ADPSK--
ORGLA05G0130500	-YISNPM---RVRTS-AAGFRALVQELTGRN-ADPSK--
BGIOSGA018119	-YISNPM---RVRTS-AAGFRALVQELTGRN-ADPSK--
Sb01g046040	-YIDNPM---RVKTS-AAGFRALVQELTGRH-ADPSK--
GRMZM2G036980	----NPV---RVKTS-VAGFRALVQELTGRH-ADPYK--
BGIOSGA017809	-YIANPM---RVTTS-EEGFRALVQELTGRH-ADPSK--
LOC_Os05g41250	-YIANPM---RVTTS-EEGFRALVQELTGRH-ADPSK--
ORGLA05G0184000	-YIANPM---RVTTS-EEGFRALVQELTGRH-ADPSK--
GRMZM2G417835	-YIANPM---RVQTS-AEGFRALVQELTGQH-ADPSK--
BRADI5G03090	-YISNPV---RVKTT-AAGFRALVQELTGLH-AGPSK--
BRADI2G21400	-YVNNPM---RVTTD-AAGFRALVQELTGRH-ADPAK--
OB05G28690	-YISNPM---RVTTS-EEGFRALVQELTGRHAADPS---
AT2G41180	-YISNPM---RVETC-PSKFRELVQELTGQDAADLP---
fgenesh2_kg.4__2247__AT2G41180	-YISNPM---RVETC-PSKFRELVQELTGQDAADLP---
Bra004604	-YISNPM---RVETC-PSKFRELVQELTGQDAAELP---
Bra000216	-YISNPM---RVETC-ASKFRELVQELTGQNAADLP---
AT3G56710	-YISNPM---RVQTC-ASKFRELVQELTGQDAVDLQ---
fgenesh2_kg.5__2103__AT3G56710	-YISNPM---RVQTC-ASKFRELVQELTGQDAVDLQ---

Bra014674	-YISNPM---RVKTC-ASKFRELVQELTGQDAVDLE---
Bra014675	-YISNPM---RVKTC-ASKFRELVQELTGQDAVDLE---
Bra007265	-YISNPM---KVKTC-ASKFRELVQELTGQDAVDQP---
GLYMA03G28360	-YISNPM---KIKTS-ASEFRALVQELTGQDAESPP---
GLYMA19G31080	-YISNPM---KIKTS-ASEFRALVQELTGQDAESPP---
POPTR_0016s03600	-YISNPM---KFKIS-ASGFRALVQELTGQDSELPD---
Vv08s0007g05180	-YIGNPR---MVKVK-ESEFRALVQELTGQDADISD---
BGIOSGA012511	-YISSPM---KLTAS-AEEFRAVVQELTGRD-SNVAD--
BRADI1G64050	-YISSPM---KLTAS-AEEFRAVVQELTGRD-SNVAD--
GRMZM2G129815	-YISSPM---KLTAS-AEEFRAVVQELTGRD-SNVAD--
LOC_Os03g20330	-YISSPM---KLTAS-AEEFRAVVQELTGRD-SNVAD--
Si037859m.g	-YISSPM---KLTAS-AEEFRAVVQELTGRD-SNVAD--
GRMZM2G318652	-YISSPM---KLTAS-AEEFRAIVQELTGRD-SNVAD--
Sb01g037040	-YISSPM---KLTAS-AEEFRAIVQELTGRD-SNVAD--
BGIOSGA023688	-YIASPM---KLTAS-PEEFRAVVQELTGRH-SNIAD--
LOC_Os07g48800	-YIASPM---KLTAS-PEEFRAVVQELTGRH-SNIAD--
ORGLA07G0213100	-YIASPM---KLTAS-PEEFRAVVQELTGRH-SNIAD--
GLYMA13G26290	-YISSPV---KVKTS-ASNFRALVQELTGQY-SNVAE--
GLYMA15G37230	-YISSPV---KVKTS-ASNFRALVQELTGQY-SNVAE--
GLYMA04G16880	-YISSPM---KVKTS-ASNFRALVQELTGQA-SNVAE--
POPTR_0003s19420	-YISSPM---KVKTS-ASKFRALVQELTGKD-SDAER--
Vv13s0084g00670	-YISSPM---KVKTS-ASKFRALVQELTGRD-SDVER--
POPTR_0001s06690	-YISSPM---KVKTS-ASEFRALVQELTGKD-SDAAR--
Vv14s0081g00190	-YISSPM---KVKASSASEFRAIVQELTGCN-SNPES--
POPTR_0004s06890	-YISSPT---MVKATNASEFRAIVQELTGKD-SKVED--
POPTR_0011s08310	-YISSPT---MVKATNASEFRAIVQELTGKD-SKVED--
POPTR_0013s04100	-YISSPT---MVKATNASEFRAIVQELTGKD-SKVED--
POPTR_0016s09710	-YISSPT---MVKATNASEFRAIVQELTGKD-SKVED--
POPTR_0019s02290	-YISSPT---MVKATNATEFRAIVQELTGKD-SKVED--
POPTR_0019s02310	-YISSPT---MVKATNATEFRAIVQELTGKD-SKVED--
POPTR_0001s01240	-YISSPM---MVKATNASEFRVIVQELTGKD-SKVED--
GLYMA03G40940	-YISNPV---LVRACDASEFRSVVQQLTGKD-TNKKV--
GLYMA19G43590	-YISSPV---LVRAYDASEFRSVVQQLTGND-SNSNK--
LOC_Os04g57030	-HVLAPE---VIKTD-ARHFRDLVQRLTG-KPAADGP--
ORGLA04G0250000	-HVLAPE---VIKTD-ARHFRDLVQRLTG-KPAADGP--
BGIOSGA014150	-HVLAPE---VIKTD-ARHFRDLVQRLTG-KPAADGP--
BRADI5G25360	-HVLAPE---VIKTD-ARHFRELVQRLTG-MPKGGGG--
Sb06g032020	-HVLAPH---IIKTE-ARHFRELVQRLTG-KPPQKGS--
Si024180m.g	-HVLAPE---IIKTE-ARHFRELVQRLTG-KPSPNGS--
OB04G35710	-HVLAPE---VIKTD-ARNFRELVQRLTG-KPATASS--
AC206638.3_FG007	-HVLAPE---VIKTE-ARDFRKVVQRLTG-MPSSQKG--
AT2G42140	-HIFAPE---IIKTD-VANFREIVQNLTG-KQDHHHH--
scaffold_403063.1	-HIFAPE---IIKTD-VSNFRKIVQNLTG-KQDHDHD--
AT3G58000	-HIFAPE---IIKTD-VANFRELVQSLTG-KPDDQRT--
scaffold_502974.1	-HIFAPE---IIKTD-VANFRELVQSLTG-KPDDHRT--
Bra007373	-HIYAPE---IIKTD-VANFRELVQSLTG-KPEDHGV--
Bra014594	-HIYAPE---IIKTD-VANFRELVQSLTG-KPEDHGV--
Bra003400	-HIFAPE---IINTD-VKNFRTLVQSLTG-KPEITKT--
scaffold_503234.1	-HIFAPE---IINTD-VKNFRTLVQSLTG-KPEITKT--
AT3G60090	-HIFAPE---IINTD-VKNFRTLVQSLTG-KPEITKT--
Bra007505	-HIFAPE---IINTD-VKNFRTLVQSLTG-KTEITKT--
Bra014514	-HIFAPE---IINTD-VKNFRTLVQSLTG-KPDITKT--
GLYMA02G29270	-HIFAPE---IIKTD-VENFRELVQKLTG-RPSGENL--
GLYMA09G17140	-HIFAPE---IIKTD-VENFRELVQKLTG-KPSGENL--
Vv08s0007g06470	-HIFAPE---IIKTD-VENFRELVQRLTG-KPSAADK--
POPTR_0006s20650	-HIFAPE---IIKTD-VANFRELVQRLTG-KPTVQKG--
POPTR_0016s04530	-HIFAPE---IIKTD-AANFRELVQRLTG-KPSDQKG--
POPTR_0009s02910	-HIFAPE---IIKTD-VADFRELVQRLTG-QPCESKG--
POPTR_0650s00200	-HIFAPE---IIKTD-VADFRELVQRLTG-QPCESKG--
AT2G44340	-HIFAPE---VIKTD-VKNFRSLVQSLTG-KPAPGEA--
fgenesh2_kg.4__2619__AT2G44340	-HIFAPE---VIKTD-VKNFRSLVQSLTG-KPAPGEA--

```
Bra004825                             -HIFAPE---VIKTD-VKNFRSLVQSLTG-KPTAGEV--
Bra037658                             -HIFAPE---VIKPD-VKNFRSLVQSLTG-KPAAVEV--
POPTR_0001s23790                      -HVFAPK---VIKTD-VANFRELVQRLTG-QPCGSEG--
ORGLA06G0176100                       -HIIAPE---IIKTD-VANFRDLVQRLTG-KQQQQQQ--
ORGLA07G0230800                       -HIIAPE---IIKTD-VANFRDLVQRLTG-KQQQQQQ--
LOC_Os06g41450                        -HIIAPE---IIKTD-VANFRDLVQRLTG-KQQQQQQ--
BGIOSGA020884                         -HIIAPE---IIKTD-VANFRDLVQRLTG-KQQQQQQ--
BGIOSGA020881                         -HIIAPE---IIKTD-VANFRDLVQRLTG-KQQQQQQ--
Si007126m.g                           -HIIAPE---IIKTD-VANFRDLVQRLTG-KPVPSAS--
GRMZM2G082118                         -HIIAPE---IIKTD-VAHFRDLVQRLTG-KAAFCAA--
GRMZM2G122447                         -HIIAPE---IIKTD-VAHFRDLVQRLTG-KPASCAA--
Sb10g024370                           -HIIAPE---IIKTD-VAHFRDLVQQLTG-KPAACAI--
BRADI1G35890                          -HIIAPE---IIKTD-AANFRDLVQRLTG-RDAADDD--
Vv13s0156g00160                       -HVFAPE---IIQTD-AANFRDLVQRLTG-KPAANFR--
GLYMA08G16790                         -HVYTPE---IIKTD-AANFRELVQRLTG-KPKEEGT--
GLYMA15G42280                         -HVYAPE---IIKTD-AANFKELVQRLTG-KPKEEEG--
GRMZM2G038622                         -HIITPE---IMKTD-VAHFRAFMQRLTG-KPACCSD--
PP1S104_35V6                          -HIFAPK---VIKTD-VANFRSTVQKLTG-RNTRKSQ--
PP1S89_5V6                            -HIFAPK---IIKTD-VANFRSTVQKLTG-KSTRKSQ--
PP1S326_18V6                          -HIFSPK---IIQTD-VANFRSTVQKLTG-KSRKKSG--
PP1S81_191V6                          -HIFAPK---VIQTD-VANFKSTVQKLTG-KSRKKSG--
AT1G21320                             -YTVTPR---IIHTH-PNNFMTLVQRLTG-QTSTSTT--
AT1G21326                             -YTVSPR---IIHTH-PNNFMTLVQRLTG-KTSTSTT--
Bra025892                             -YTVSPK---IIHTH-PNNFMTLVQRLTG-KTSTPTI--
fgenesh2_kg.1__2319__AT1G21320        -YTVSPK---IIHTH-PNNFMTLVQRLTG-KTSTSTN--
Bra012276                             -YTVSPK---IIHTH-PNNFMGLVQRLTG-NTSASTA--
GLYMA04G41820                         -YTVSPK---VIHTT-PSDFMNLVQRLTG-SSSSSSV--
GLYMA14G29580                         -YTVSPK---VIHTT-PSDFMSLVQRLTG-SSSSSSS--
Vv18s0166g00090                       -YTVSPK---IIHTQ-PSEFMTLVQRLTG-LSSSSSS--
POPTR_0002s07140                      -YTVSPK---VIHTN-PNDFMTLVQRLTG-SSSTSTS--
POPTR_0005s21160                      -YTLSPK---VIHTN-PNDFMTLVQRLTG-SSSTSTC--
AT3G18360                             -YTHTPR---IIHTN-PKDFMALVQKLTG-MTHSDED--
fgenesh2_kg.3__2037__AT3G18360        -YTHTPR---IIHTN-PKDFMALVQKLTG-MSRSDED--
Bra037588                             -YTNTPK---VIHTN-PKDFMALVQKLTG-MSHSEED--
GLYMA07G10290                         -YTHSPK---VIHTQ-PKDFMSLVQKLTG-LSRSDEK--
GLYMA09G31600                         -YTHSPK---VIHTQ-PKDFMSLVQKLTG-LSRSGEE--
POPTR_0012s05230                      -YTHSPK---IIHTN-PKDFMSLVQKLTG-LSRSEED--
POPTR_0015s03730                      -YTHSPK---IIHTN-PKDFMALVQKLTG-LSQSEDI--
POPTR_0010s13360                      -YTHSPK---VIHTQ-AKDFMALVQNLTG-LSRSNNQ--
Vv01s0011g03650                       -YTHSPK---IIHTQ-ARDFMALVQKLTG-LSSSSDS--
GLYMA10G41970                         -YTESPK---IIHTK-AKDFMALVQRLTG-RSSSTND--
GLYMA20G25070                         -YTESPK---VIHTK-AKDFMALVQRLTG-RSSTNDN--
BGIOSGA031344                         -YTHSPK---VIRTS-PRDFMSIVQRLTG-LDSARTA--
ORGLA10G0146300                       -YTHSPK---VIRTS-PRDFMSIVQRLTG-LDSARTA--
BRADI3G34440                          -YTHSPK---VIRTS-PRDFMSIVQKLTG-LDSASAN--
GRMZM2G174650                         -YTHSPK---VIRTN-PRDFMSIVQKLTG-LDGPGHH--
Si039726m.g                           -YTHSPK---VIRTN-PRDFMSIVQKLTG-LDSPGPA--
Sb01g028215                           -YTHSPK---VIRTN-PRDFMSIVQKLTG-LDSRHGR--
OB10G26590                            -YTHSPK---VIRTN-PRDFMSIVQKLTG-LETNKHG--
GLYMA08G04650                         -YTHPPK---VIHTH-ARNFMELVQKLTG-LYRTDPE--
SELMODRAFT_409496                     -HTYSPK---VIETD-ATNFMWLVQRLTG-SSDTRRR--
SELMODRAFT_416074                     -HTYSPK---VIETD-ATNFMWLVQRLTG-SSDTRMR--
SELMODRAFT_447774                     -HTYSPK---VIQTS-TQDFMELVQRLTG-SSDTRLR--
SELMODRAFT_448561                     -HTYSPK---VIQTS-TQDFMELVQRLTG-SSDTRLR--
PP1S23_240V6                          -HTYSPK---VIHTQ-PDDFMSLVQKLTG-SSDTRLR--
PP1S310_23V6                          -HTYSPK---VIHTQ-PDDFMSLVQKLTG-SSDTRLR--
PP1S4_213V6                           -HTYSPK---VIHTE-PDDFMSLVQKLTG-SSDTRLR--
PP1S23_248V6                          -HTYSPK---VIHTD-PNGFMSLVQKLTG-SSDTRLR--
PP1S57_98V6                           -HTYSPK---VIYAE-PDEFMSLVQKLTG-SSDTRSR--
AT1G68450                             -YAHSPK---VIHTR-AEDFMALVQRLTG-LDEIIRR--
scaffold_201685.1                     -YAHSPK---VIHTR-AEDFMALVQRLTG-LDEIIRR--
```

Bra033934	-YAHSPK---VIHTR-AENFMALVQRLTG-LEGIRRR--
BGIOSGA020926	-YMVSPK---IIHVE-AHEFMSLVQRLTG-PGAAAAG--
LOC_Os06g40090	-YMVSPK---IIHVE-AHEFMSLVQRLTG-PGAAAAG--
GRMZM2G099691	-YMVSPK---VIHVE-AHEFLPLVQRLTG-PEAGRGD--
Si007257m.g	-YMVSPK---VIHVE-AHEFMPLVQRLTG-PDAGRGD--
GRMZM2G069169	-YMVSPK---VIHVD-AHEFMPLVQRLTG-PEAGRGD--
Sb10g023570	-YMVSPK---VIHVE-AHEFLPLVQRLTG-PEAAAGR--
BRADI1G36630	-YMVSPK---VIHVE-AHEFMSLVQRLTG-PDGGDDQ--
GLYMA03G36330	-HLKSPK---VIHVR-PEEFMSLVQQLTR-NPVSAAF--
GLYMA19G38980	-HLKSPK---VIHVR-PEEFMSLVQQLTG-NPVSAAA--
LOC_Os02g33600	-YDASPK---VIHTR-PSEFMALVQRLTG-PGSAAVA--
ORGLA02G0167700	-YDASPK---VIHTR-PSEFMALVQRLTG-PGSAAVA--
BGIOSGA008380	-YDASPK---VIHTR-PSEFMALVQRLTG-PGSAAVA--
BRADI3G45220	-YDASPK---VIHTQ-PSEFMALVQRLTG-PGLPAAH--
LOC_Os04g34050	-YDASPK---IIHAK-PNEFMALVQRLTG-PGSGPPA--
ORGLA04G0092300	-YDASPK---IIHAK-PNEFMALVQRLTG-PGSGPPA--
BGIOSGA016385	-YDASPK---IIHAK-PNEFMALVQRLTG-PGSGPPA--
Si010684m.g	-YDASPK---VIHVK-PSEFMALVQSLTG-PGSGAPQ--
GRMZM2G369742	-YDASPK---VIHAK-PSEFMALVQRLTG-PGAQAQH--
Sb06g016290	-YDASPK---VIHAK-PSEFMALVQRLTG-PGAGGSG--
GRMZM2G346837	-YDASPK---VIHTK-PGDFMALVQRLTG-PGSTSQA--
Sb04g022090	-YDASPK---VIHTK-PGDFMALVQRLTG-PGSISQA--
Si019357m.g	-YDASPK---VIHTK-PGDFMALVQRLTG-PGSSSQA--
GRMZM2G060720	-YDASPK---VIHVK-PSDFMALVQHLTG-PGGSGTP--
BRADI5G09730	-YDASPK---IIHTK-PSEFLALVQRLTG-PSSSAGP--
scaffold_302208.1	-YAVSPK---VVHAT-ASEFMNVVQRLTG-ISSGVFL--
scaffold_302210.1	-YAVSPK---VVHAT-ASEFMNVVQRLTG-ISSGVFL--
AT3G18690	-YAVSPK---VVHAT-ASEFMNVVQRLTG-ISSGVFL--
Bra001716	-YAVSPK---VVHTT-TSDFMNVVQRLTG-ISSEVFL--
Bra037569	-YAVSPK---VVHTT-ASDFMNVVQRLTG-ISSAVFL--
Bra022345	-YAVSPK---VVHTT-TSDFMNVVQRLTG-ISAGVFH--
GLYMA04G39270	-YSVSPK---VIHVT-PGDFMDVVQRLTG-ASSGEYP--
GLYMA06G15650	-YSVSPK---VIHVT-PGDFMDVVQRLTG-ASIGEEP--
GLYMA05G32350	-YTVSPK---VLHVT-VSDFMNVVQRLTG-PSSGAEP--
GLYMA08G15620	-YAVSPK---VLHVP-AGDFMNVVQRLTG-PSSGDVS--
POPTR_0005s05910	-YAVSPK---IIHAE-ESNFMAVVQRLTG-LSSADFS--
POPTR_0007s03770	-YAVSPK---TIHTE-ESNFMAVVQRLTG-LSSGDFF--
LOC_Os03g57520	-YEHTPK---VVHAR-PQEFMTVVQRLTG-KPPAAPP--
ORGLA03G0337400	-YEHTPK---VVHAR-PQEFMTVVQRLTG-KPPAAPP--
ORGLA07G0259400	-YEHTPK---VVHAR-PQEFMTVVQRLTG-KPPAAPP--
BGIOSGA013698	-YEHTPK---VVHAR-PQEFMTVVQRLTG-KPPAAPP--
Sb01g005740	-YEHTPK---VIHAR-PQEFMTVVQRLTG-KPPVAPA--
GRMZM2G174210	-YEHTPK---VIHAR-PQEFMTVVQRLTG-KPPATTT--
BRADI1G06260	-YEHTPK---VVHAR-PQEFMTVVQRLTG-RQPTPSP--
Si037845m.g	-YEHTPK---VVHAR-PQEFMTVVQRLTG-KQPAPAA--
GRMZM2G124290	-YEHTPR---VIHAS-PQEFMTVVQRLTG-KRPRAAP--
LOC_Os07g06750	-YELTPR---VVHVE-QEEFMAVVQKLTG-GKQQPAA--
ORGLA07G0032700	-YELTPR---VVHVE-QEEFMAVVQKLTG-GKQQPAA--
BGIOSGA025193	-YELTPR---VVHVE-QEEFMAVVQKLTG-GKQQPAA--
BGIOSGA025194	-YELTPR---VVHVE-PEEFMAVVQKLTG-NRKLSTA--
LOC_Os07g06760	-YELTPR---VVHVE-PEEFMAVVQKLTG-NRKLSTA--
ORGLA07G0032800	-YELTPR---VVHAE-PEEFMAVVQKLTG-NRKLSTA--
BRADI1G56950	-YERTPR---VVHAR-PEEFMAVVQRLTG-KQQQPTT--
BGIOSGA025195	-YELTPR---VVHAQ-PEEFRAIVQKLTG-KPSTATA--
LOC_Os07g06790	-YELTPR---VVHAQ-PEEFRAIVQKLTG-KPSTATA--
ORGLA07G0032900	-YELTPR---VVHAQ-PEEFRAIVQKLTG-KPLTATA--
OB07G12890	-YELTPR---VVHAR-PEEFRAVVQKLTG-KQSAAPP--
Sb02g003505	-YEHTPR---VIHAR-PDEFKALVQRLTG-RPQPTAG--
Si033098m.g	-YEHTPK---VIHAR-PDEFKALVQRLTG-REQPVVQ--
GRMZM2G354123	-YEHTPK---VIHAR-PDEFKALVQRLTG-RRAGGGG--
GRMZM2G355499	-YEHTPK---VIHVH-ADEFKALVQRLTG-RQLTPPG--

BGIOSGA027834	-YTMSPK---VVHAN-AADFMSVVQRLTG-APPTAPP--
LOC_Os08g01260	-YTMSPK---VVHAN-AADFMSVVQRLTG-APPTAPP--
OB08G10290	-YTMSPK---VVHAN-PADFMSVVQRLTG-APRTTAP--
GRMZM2G003669	-YTVSPK---VVHAK-PSEFMSVVQRLTG-ARGHGAT--
Sb07g000390	-YTVSPK---VVHAE-PGEFMSVVQRLTG-ARGATAS--
Si015774m.g	-YTLSPK---VVHAD-ASEFMSVVQRLTG-ATASSSS--
GRMZM2G475276	-YVESPR---VVHAH-PAEFKSVVQRLTGTAPALPLP--
Sb06g033670	-YVESPR---VVHAH-PAEFKSVVQRLTG-APAAPAP--
Si025350m.g	-YVESPK---VVHAH-PGEFKSVVQRLTG-APPPPSA--
Bra029432	-VVVSPK---VVHTT-LSDFRSVVHRLTG-VNNSVSQ--
POPTR_0005s22570	-KVLRPK---VYITD-SSSFKRLVQELTG-NGKTIPS--
Vv12s0028g00500	-KVLRPK---VYITD-SSSFKKLVQELTG-NGTSVAP--
POPTR_0014s13800	-RALRPR---VYITD-TSKFKTLVQELTG-NGKGSSS--
SELMODRAFT_138089	-QIFNPT---IIRTD-AASFRELVQELTG-MNYQPAI--
SELMODRAFT_443192	---LPPTTRPVIRTD-AASFRELVQELTG-MNYQPAI--
AT1G78310	-HQHQPP---VYN-INKNDFRDVVQKLT-GSPAHERI--
Bra035028	-HQHQPP---VYN-INKNDFRDVVQKLT-GSPAHERI--
fgenesh2_kg.2__2129__AT1G78310	-HQHQPP---VYN-INKNDFRDVVQKLT-GSPAHERI--
POPTR_0002s10010	-HQHQPP---VYN-INKNDFRDVVQKLT-GSPAHERF--
Vv18s0001g06350	-QQHQPP---VYN-INKNDFRDVVQKLT-GSPAHERF--
GLYMA04G10780	-LQHQPP---VYN-INKNDFRDVVQKLT-GSPAHDRI--
GLYMA06G10630	-VQHQPP---VYN-INKNDFRDVVQKLT-GSPAHDRI--
Bra008356	-HQHQPP---VYN-ISKSDFRDVVQKLT-GSPAHERI--
POPTR_0005s17320	-GQQQPP---VYN-INKSDFRDLVQKLT-GSPAHERF--
BGIOSGA012021	-PPPQPP---VYN-IDKSDFRDVVQKLT-GSPWHLLP--
LOC_Os03g09045	-PPPQPP---VYN-IDKSDFRDVVQKLT-GSPCHLLP--
GRMZM2G420357	-PPPQPP---VYN-IDKSDFRDVVQKLT-GSPSHLLP--
GRMZM5G864133	-PPPQPP---VYN-IDKSDFRDVVQKLT-GSPSHLLP--
BRADI1G71870	-PPPQPP---VYN-IDKSNFRDVVQKLT-GSPSHLLP--
Sb01g044570	-PPPQPP---VYN-IDKSNFRDVVQKLT-GSPSHLLP--
GRMZM2G158976	-PQPQPQ---VYN-ISKNQFRDIVQQLTAGTPSPPPP--
Sb01g027990	-PQPQPQ---VYN-ISKNQFRDIVQQLTAGTPSPPPP--
BGIOSGA031335	-PQPQPQ---VYN-ISKNQFRDIVQQLTAGTPSPPPP--
ORGLA10G0147800	-LQPQPQ---VYN-ISKNQFRDIVQQLTAGTPSPPPP--
POPTR_0001s02480	-QQPQPQ---VYN-ISKNDFRNIVQQLT-GSPSQEPL--
Vv02s0012g01280	-QQPQPQ---VYN-ISKNDFRNIVQQLT-GSPSQEPL--
GLYMA01G02280	-QQPQPQ---VYN-ISKNDFRDIVQQLT-GSPSQDPP--
GLYMA08G36730	-QQPQPQ---VYN-ISKNDFRDIVQQLT-GSPSQSQD--
GLYMA08G36750	-QQPQPQ---VYN-ISKNDFRDIVQQLT-GSPSQSQD--
GLYMA08G36740	-PQPQPQ---VYN-ISKNDFRDIVQQLT-GSPSQSQD--
GLYMA01G12940	-QQPQPQ---VYN-ISKNEFRDIVQKLT-GSPSQDPQ--
GLYMA02G37130	-SESQPQ---VYN-ISKNDFRDMVQKLT-GSPGHTPQ--
AT2G35230	-LQTQPQ---VYN-ISKNDFRSIVQQLT-GSPSRESL--
fgenesh2_kg.4__1549__AT2G35230	-PQTQPQ---VYN-ISKNDFRSIVQQLT-GSPSRECL--
Bra005358	-PQGQPQ---VYN-ISKTDFRSMVQQLT-GSPARESL--
Bra017329	-PQAQPQ---VYS-VSKNDFRSVVQQLT-GSPSRESL--
Bra023004	-PQAQPQ---VYN-INRSDFRSIVQQLT-GSPSRESL--
Bra035492	-PQLKTQ---VYI-IDKEDFKSIVQQLT-SNQSCEFL--
AT5G46780	-QNPQAL---VYN-INKTDFRSIVQQLT-GLGSTSSV--
scaffold_800150.1	-QNPQAL---VYN-INKTDFRSIVQQLT-GLGSTSSV--
Bra024996	-QNPQAL---VYN-INKTDFRSIVQQLT-GLGSASSV--
Bra022063	-QNPQAL---FFN-INKTDFRSIVQQLT-VLGSASSV--
SELMODRAFT_410083	KPLPTSK---VYN-TDPMSFRELVQQLT-GNSSTGTS--
SELMODRAFT_442289	KPLPTSK---VYN-TDPMSFRELVQQLT-GNSSTGTS--
PP1S1_173V6	-PRLAPQ---VYK-TEPSDFRSLVQQLT-GTSQPANP--
PP1S38_21V6	-PRLAPQ---VYK-TEPSDFRSLVQQLT-GTSLPANPPP
PP1S75_36V6	-PRPAPQ---VYK-TEPAEFRSLVQQLT-GTSSPE-PS-
PP1S23_345V6	-HTYSPK---VYK-IKPEEFLDLVQKLT-GRPTTEES--
PP1S252_83V6	-HTYSPK---VYK-IKPEEFLDLVQKLT-GRPAADEV--
PP1S252_73V6	-HTKSPK---VYK-IQPEEFLELVQKLT-GRSVPELP--
PP1S50_57V6	-HAHSPI---VYK-IEPEKFLSLVQKLT-GRPTESLA--

```
PP1S154_1V6                         -HTYSPM---IYK-VQPEEFLEFVQKLT-GQKVRKPR--
PP1S106_95V6                        -RKSSPQ---VYK-IRPEEFLDVVQRLT-GQSLPHPP--
fgenesh2_kg.3__3175__AT2G22880      -QRMHPK---VYR-VKPVNFKELVQRLT-GA-HDHEQE-
fgenesh2_kg.4__210__AT2G22880.      -QRMHPK---VYR-VEPVNFKELVQRLT-GA-HDHEQE-
AT2G22880                           -QRMHPK---VYR-VEPVNFKELVQRLT-GA-EDVEQE-
Bra039937                           -QRMHPR---VYR-VEPVNFKELVQRLT-GAPQDHER--
Bra010608                           -NPMHPH---VYR-VEPVNFKELVQRLT-GA-PEHEPV-
fgenesh2_kg.7__277__AT4G37710.      -NPMHPH---VYR-VEPVNFKELVQRLT-GA-PEHEPV-
AT4G37710                           -NPMHPH---VYR-VEPVNFKVLVQRLT-GA-PEHETV-
Bra017849                           -KSMHPQ---VYR-VEPANFKELVQRLT-GA-PEHDDV-
BGIOSGA010063                       -GAAPPK---VYR-VAPRDFRELVQRLT-GAGTAAPA--
BGIOSGA010064                       -GAAPPK---VYR-VAPRDFRELVQRLT-GAGTAAPA--
LOC_Os03g47280                      -GAAPPK---VYR-VAPRDFRELVQRLT-GAGTAAPA--
ORGLA03G0272300                     -GAAPPK---VYR-VAPRDFRELVQRLT-GAGTAAPA--
GRMZM2G118172                       -GPPPPK---VYR-VEPRDFRELVQRLT-GAGTPAAA--
Sb01g012100                         -GPPPPK---VYR-VEPRDFRELVQRLT-GAGTPSAA--
GRMZM2G129140                       -PPPPPK---VYR-VEPRDFRELVQRLT-GAGTPAAA--
Si038017m.g                         -PPPPPK---VYR-VEPRDFRALVQRLT-GAGGGSEA--
BRADI1G12580                        -PPAPPK---VYR-VEPRGFRELVQRLT-GSGTAPSM--
BGIOSGA035768                       -GPAPPK---VYR-VEPREFRDLVQRLT-GAPAPPPP--
BRADI4G00780                        -APAPPR---VYR-VEPREFRDLVQRLT-GAPRHPQA--
Sb08g022890                         -PPPPPK---VYR-VEPREFRDLVQRLT-GAPPTALR--
Si023655m.g                         -PPPPPK---VYR-VEPREFRDLVQRLT-GAPPAAAA--
GRMZM2G059064                       -AATPPK---VYR-VEPREFRDLVQRLT-GAPPPTAL--
GLYMA01G40320                       -APTPIR---VYK-VDAINFRDLVQQLT-GAPEFKPD--
GLYMA11G04970                       -APTPIR---VYK-VDAINFRDLVQQLT-GAPEFKPA--
GLYMA05G22760                       -PPTPIK---VYK-VDAINFRDVVQQLT-GAPEHESQ--
GLYMA17G17210                       -PPTPVK---VYK-VDAINFREVVQQLT-GAPKHKPQ--
POPTR_0007s14810                    -PPTPPR---VYK-VDPINFRDLVQKLT-GAPEPEPV--
POPTR_0014s00870                    -PPTPPR---VYK-VDPINFRDLVQKLT-GAPKKEPE--
Vv00s0471g00020                     -PPTPPR---VYK-VESANFREVVQQLT-GSPEFQSR--
LOC_Os11g12790                      ----TTK---MYV-ADKSTFKELVQRLT-GQPPAEAA--
ORGLA11G0068400                     ----TTK---MYV-ADKSTFKELVQRLT-GQPPAEAA--
BGIOSGA034242                       ----TTK---MYV-ADKSTFKELVQRLT-GQPPAEAT--
OB11G16060                          TEPSPTR---IYVTANPSTFKELVQRLT-GQPTGEAV--
BGIOSGA032413                       -EPSDGE---VYV-VHPTHFRTVVQQLT-GAPPPVAN--
LOC_Os10g01240                      -EPSDGE---VYV-VHPTHFRTVVQQLT-GAPPPVAN--
OB10G10130                          -DPLDAE---VYV-VHPTQFRSVVQQLT-GAPPGAAH--
Si038923m.g                         -DPADTS---VYV-VHPTQFRAVVQQLT-GAGAASSP--
GRMZM2G420630                       -DPADTA---VHV-VRPDQFRAVVQQLT-GAASSQPP--
Sb01g027490                         -DPADTA---VYV-VQPDQFRAVVQQLT-GAASSPPP--
Si038033m.g                         -DPTATA---VYV-VHPTQFRTIVQQLT-GAASSPPP--
Sb01g027480                         -EHAATT---VYV-VHPTQFRTVVQQLT-GAASPPPL--
GRMZM2G083285                       -RLKTTE---VIE-VQLADFKAAVQRLT-GLGAAVAA--
                                                       *   ::  *
```
